# Switchable Site-Selective Benzanilide C(sp^2^)-H Bromination via Promoter Regulation

**DOI:** 10.3390/molecules29122861

**Published:** 2024-06-16

**Authors:** Yonghui Sun, Qiyu He, Xucheng Lv, Naizhen Zhang, Wei Yan, Jianghui Sun, Lili Zhuang

**Affiliations:** State Key Laboratory of Bioactive Substance and Function of Natural Medicines, Beijing Key Laboratory of Active Substances Discovery and Druggability Evaluation, Institute of Materia Medica, Chinese Academy of Medical Sciences and Peking Union Medical College, Beijing 100050, China; heqiyu@imm.ac.cn (Q.H.); ytxuchenglv@163.com (X.L.); zhangnaizhen@imm.ac.cn (N.Z.); yanwei46@alu.sxu.edu.cn (W.Y.); sunjianghui@imm.ac.cn (J.S.); zhuanglili@imm.ac.cn (L.Z.)

**Keywords:** switchable regioselectivity, benzanilide, C(sp^2^)-H bromination, promoter regulation

## Abstract

Regioselective benzanilide bromination that generates either regioisomer from the same starting material is desirable. Herein, we develop switchable site-selective C(sp^2^)-H bromination by promoter regulation. This protocol leads to regiodivergent brominated benzanilide starting from the single substrate via selection of promoters. The protocol demonstrates excellent regioselectivity and good tolerance of functional groups with high yields. The utility effectiveness of this method has been well exemplified in the late-stage modification of biologically important molecules.

## 1. Introduction

**Figure 1:**
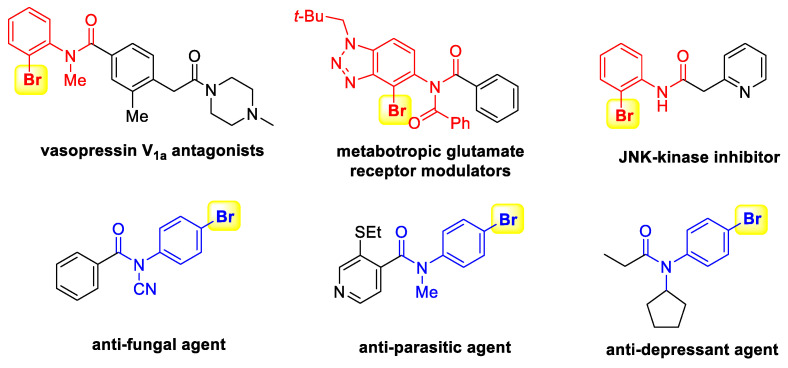
Biologically important molecules containing brominated benzanilides.

**Scheme 1:**
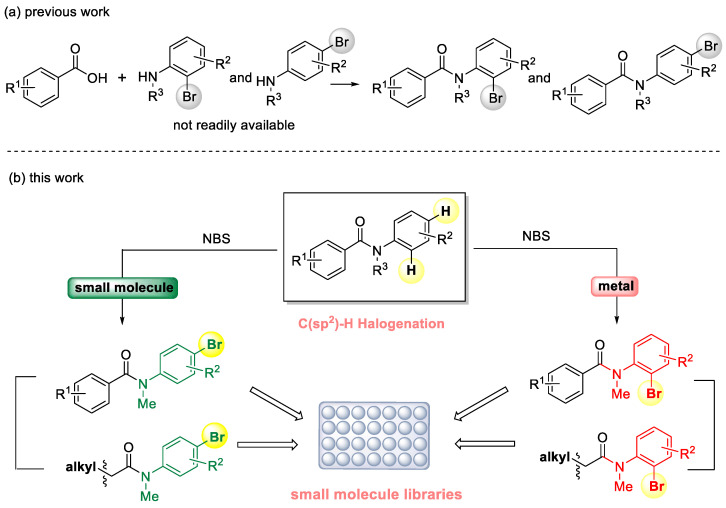
Region-selective synthesis for accessing brominated benzanilides by C(sp^2^)-H functionalization.

Brominated benzanilides are an important class of structures essential for the function of almost all modern pharmaceuticals and agrochemicals [1,2,3,4]. Because of the steric hindrance of -Br, the bromo group located in the *ortho* or *para* position of the benzanilide molecule produces different biological activities [5,6]. Indeed, in anti-hypertensive compounds and anti-cancer agents, the bromo group is in the *ortho* position, while it is in the *para* position in anti-depressant drugs or anti-microbials agents (Figure 1). Therefore, the acquisition and rapid screening of small molecule libraries containing diverse brominated benzanilides is an essential aspect of hit compounds discovery. More importantly, based on the diversity-oriented synthesis (DOS) strategy, C-C or C-N bond formation via cross coupling starting from brominated benzanilides will efficiently generate structurally diverse compound libraries, such as benzidine, phenylenediamine, etc. [7,8,9,10,11,12]. To date, various important small molecule modulators have been identified for “undruggable” targets from unbiased phenotypic screening of DOS-derived compound libraries [13,14,15,16]. However, the classic approach to the generation of brominated benzanilides involves a two-step process: bromination of aniline followed by the benzoylation of the resulting brominated product, which suffers from limitations such as poor selectivity, low yields, complex purification method, etc. (Scheme 1a) [17,18,19].

Because of the challenge of selectivity and efficiency, the practical application of regioselective halogenation of C-H activation in a substrate containing two or more arenes is still uncommon [20,21]. Over the past decade, direct functionalization of C(sp^2^)-H bonds has emerged as a powerful tool for generating new C_Ar_-halogen bonds [22,23,24,25,26]. Among these, direct C-H cleavage, transition-metal-catalyzed functionalization of anilids [27,28,29,30], and hexafluoroisopropanol [31,32,33,34] (HFIP) promoted C-H activation of anilines are the most similar. Although these impressive approaches can be employed to construct halogenated anilids, to the best of our knowledge, directed C-H halogenation in terms of regioselectivity has not been previously described. For instance, knowledge about regioselective C(sp^2^)-H bromination of *N*-methyl-benzanilides and its corresponding application in synthesis of biologically important chemicals is still surprisingly underdeveloped. Stimulated by the previous diversification of bioactive agents by a controlled catalyst or photo-induced construction of C-halogen bonds reported by Yu [35,36,37] and MacMillan [38,39,40,41], we envisioned that the regioselective C-H functionalization of benzanilides could be achieved via utility of different catalysts (Scheme 1b). Herein, we report the use of HFIP and Pd(II) as promoters for regioselective C-H bromination of benzanilides to provide a convenient route for synthesis of diverse brominated benzanilide derivatives. When Pd(II) is employed as promoter, bromination occurs on the *ortho* position of aniline, while a reversed regioselective product is obtained with an HFIP promoter. Preliminary studies of regioselective bromination on other acylanilide substrates are also described.

## 2. Results and Discussion

Since different promoters generate various types of transition states based on the detailed mechanisms, regioselective bromination of benzanilides may occur with different promoters by activating distinct protons. To test our hypothesis, we conducted a model study using benzanilide (**1**) in the presence of NBS in the TFA/TFAA solvent system with Na_2_S_2_O_8_ as the terminal oxidant (Table 1). After the screening of palladium catalysts, we discovered that only Pd(OAc)_2_ and Pd(TFA)_2_ could promote C-H activation, and bromination occurred on the *ortho* position of the amino group to provide the brominated product **2a** (entry 3–4). In contrast, PdCl_2_ and Pd/C failed to give the brominated benzanilides. Next, we turned our attention to other transition metal catalysts. However, cobalt, copper or nickel failed to promote the transformations (entry 7–8, 10–11). Delightfully, after a comprehensive screening of additives, we found that brominated product **2b** could also be readily prepared under the condition of hexafluoroisopropanol (HFIP), and the bromination reaction occurred on the *para* position of the amino group (entry 9). The reactions typically proceeded to completion within 3 h at 70 °C. This initial result showed that Pd(II)- or HFIP-promoted regioselective bromination, with anilid as an effective directing group, could occur under optimized conditions (entry 6, 12).

**Figure 2:**
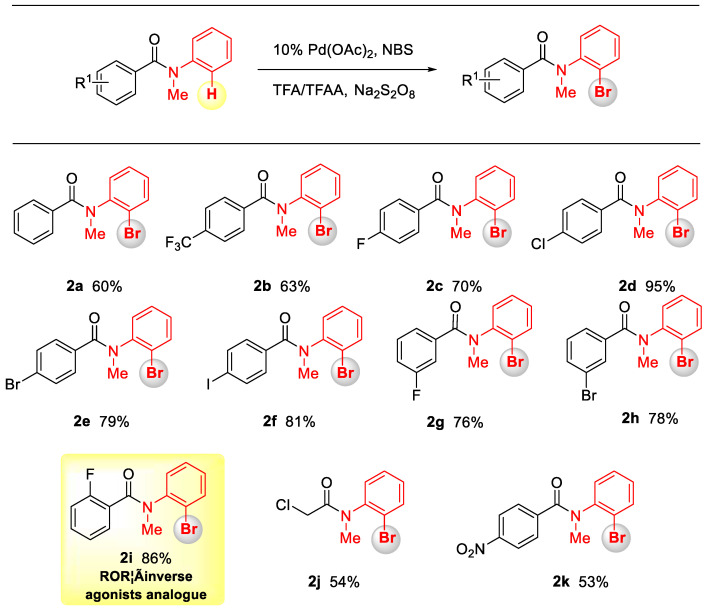
Substrate scope of Pd(II) in C(sp^2^)-H bromination (isolated yield).

**Figure 3:**
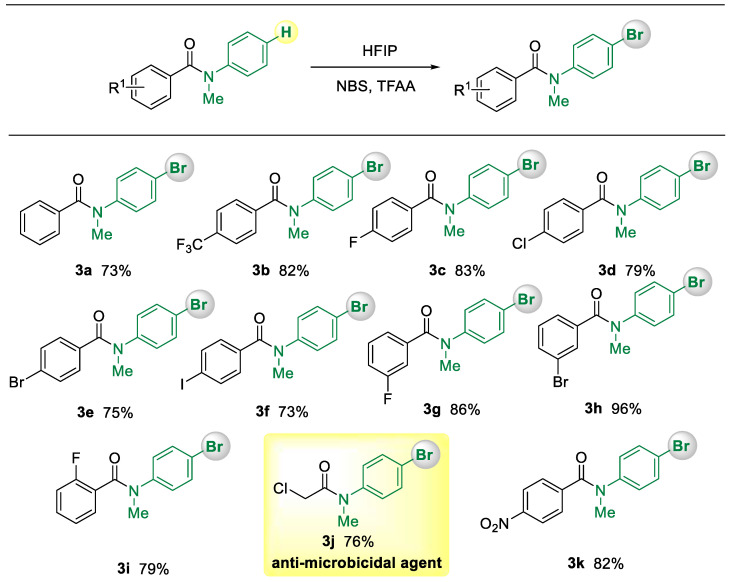
Substrate scope of HFIP in C(sp^2^)-H bromination (isolated yield).

With these results of the optimized conditions of Pd(II)- or HFIP-promoted regioselectivity in hand, we set out to explore the scope and robustness of this new method. As illustrated in Figure 2 and Figure 3, a variety of benzanilides were smoothly transformed into the corresponding brominated anilide products (**2a**–**2j**, **3a**–**3j**) in excellent yields. The *para*-, *meta*-, and *ortho*-substituted benzoyl groups were well tolerated; only specific regioselective brominated benzanilides were observed. It is worth mentioning that different substrates use different reaction temperatures. Some substrates with electron-withdrawing groups (CF_3_- and NO_2_-) may require a higher reaction temperature because they have lower electron cloud density. Therefore, in these reactions, conditions should be tough. With all substrates, the corresponding regioselective bromination products could be consistently obtained. When Pd(OAc)_2_ was used as a catalyst, the bromination reaction occurred on the *ortho* position of the amino group, and *para* bromobenzanilides were obtained when HFIP was involved. Notably, a special example was 2-chloro-*N*-methyl-*N*-phenylacetamide. We were delighted to observe that anilide, containing an aliphatic acyl group rather than the benzoyl motif, could also be employed successfully to provide regioselective bromination products (**2j**, **3j**). This phenomenon expands the application scope of this novel method for regioselective modification of anilides and benefits the diversity of small molecule libraries available for further bioactivity screening.

**Scheme 2:**
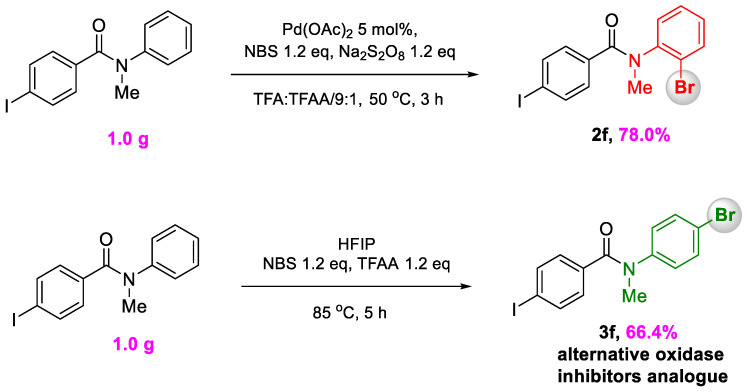
Gram-scale synthesis application of *ortho*-brominated benzanilides.

Since brominated iodobenzanilides are widely utilized in bioactive agents [42,43,44,45], to prove both the synthetic utility effectiveness of this method for large-scale synthesis, we prepared **2f** and **3f** on a gram scale under the optimized reaction conditions with only a 5 mol% catalyst (Scheme 2). Compared with conventional approaches requiring brominated alkylanilines, which are generally expensive, this method with operational simplicity is advantageous for rapid access to various kinds of *ortho* and *para* brominated benzanilides. We further showed the feasibility of this new reaction by performing the synthesis of biologically important molecules (Figure 2 and Figure 3). With Pd(II) as a catalyst, 2-fluoro-*N*-methyl-*N*-phenylbenzamide was readily converted to brominated compound **2i** as an RORγ inverse agonists analogue in a good yield [46]. RORγ is mainly expressed in immune cells, promoting the differentiation of Th17 cells and producing the key factor IL-17. RORγ plays the corresponding biological functions in a variety of important inflammatory pathways. Recent studies have found that an RORγ inverse agonist is a new therapy for the treatment of castration-resistant prostate cancer (CRPC). This type of brominated compounds can be conveniently synthesized by our method with excellent chemoselectivity, which provides an opportunity to generate novel anti-malignancy agents, such as a the DUBTAC ligand for stabilizing RORγ from degradation. Accordingly, HFIP-promoted bromination could conveniently transform 2-chloro-*N*-methyl-*N*-phenylacetamide into anti-microbicidal agent **3j [47]**, while its *ortho*-brominated analogue was readily synthesized via a Pd(II) catalyst with good regioselectivity.

**Scheme 3:**
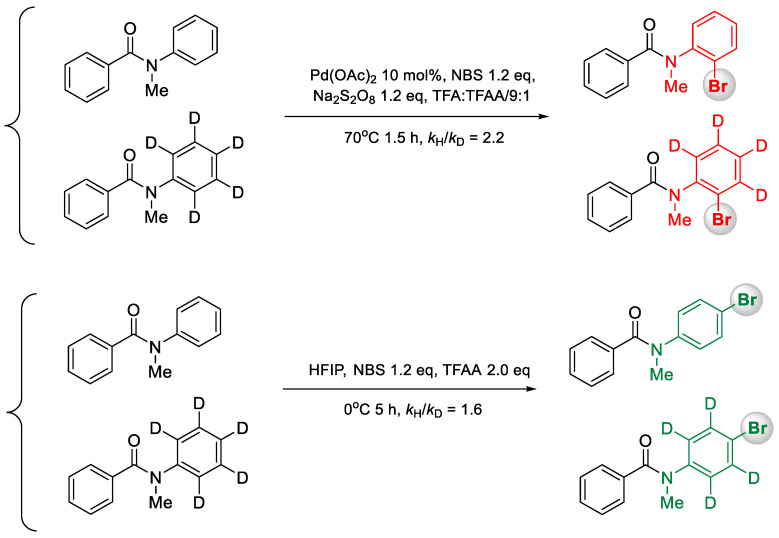
Mechanism study of the bromination reaction via kinetic isotope effect experiments.

Further studies were performed to gain insight into the mechanisms, which are shown in Scheme 3. A consistent and significant kinetic isotopic effect value of 2.2 was observed with Pd(II) as a catalyst, indicating that C–H cleavage might be involved in the rate-limiting step of the *ortho*-bromination reaction. In parallel, with HFIP as the promoter, a KIE value of 1.6 was obtained, suggesting that the C–H activation step in *para*-bromination was partially turnover limiting. Taken together, during the whole transformation, other elementary steps (such as the ligand exchange step) either before or after C–H activation were also critical to the catalytic turnover rate.

**Scheme 4:**
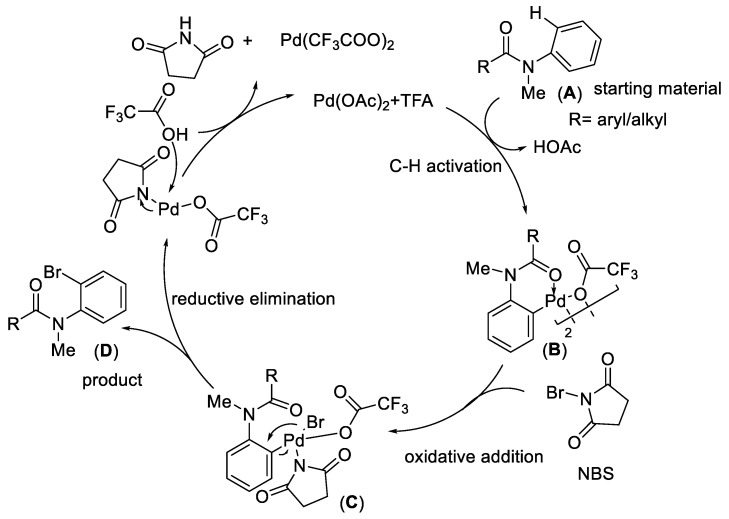
Proposed mechanism of *ortho*-bromination of *N*-methyl-benzanilides.

**Scheme 5:**
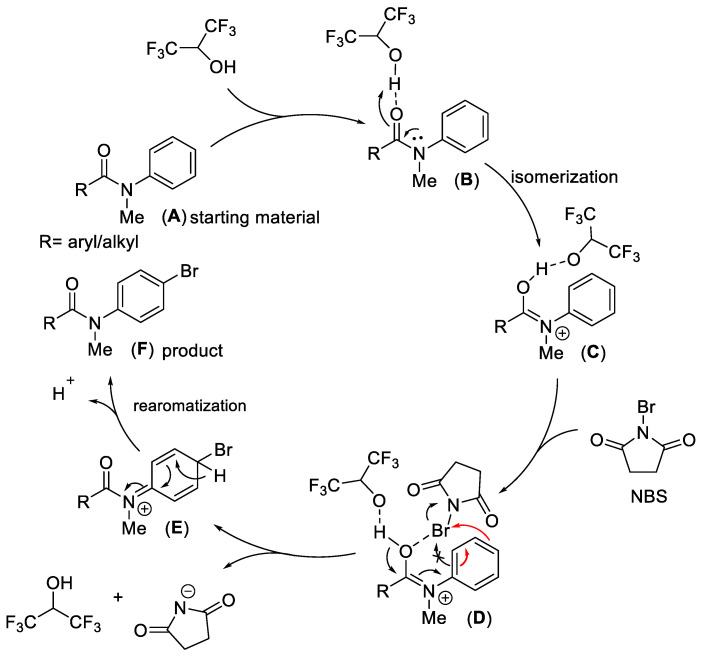
Proposed mechanism of *para*-bromination of *N*-methyl-benzanilides.

Although detailed mechanisms remain to be ascertained, with all these results in hand, the proposed mechanisms of *ortho* and *para* bromination are shown in Scheme 4 and Scheme 5, respectively. With Pd(II) as the promoter, the catalytic cycle is proposed to involve four steps: (i) the ligand was transfered to form Pd(TFA)_2_, and palladacycle **B** was generated, (ii) oxidation by NBS to provide Pd(IV) intermediate **C**, (iii) a C–Br bond was formed via reductive elimination, (iv) Pd(II) was released to coordinate a new substrate. On the other hand, HFIP-promoted bromination also required an amide bond as the directing group. However, due to the steric repulsion from HFIP, intermediate **D** was more crowded, so the C–Br bond formation occurred on the *para* position of the amino group.

## 3. Materials and Methods

### 3.1. Materials

All commercial materials (Bidepharm (Shanghai, China), J&K Chemical Ltd. (Beijing, China), Energy Chemical (Shanghai, China), Alfa Aesar (Shanghai, China), Konoscience (Beijing, China), Mreda (Beijing, China) and Aladdin (Shanghai, China) were utilized without further purification. All benzoic acids, *N*-methylanilines, and anilines were purchased. All solvents were of analytical grade. The NMR spectra were generated on a Bruker AVANCE NEO 700 MHz (Bruker, Karlsruhe, Germany), JEOL JNM-ECZL600G 600 MHz (JEOL, Tokyo, Japan), JEOL-JNM-ECZ500R 500 MHz (JEOL, Tokyo, Japan), Q.ONE INSTRUMENTS-Quantum-1 400 MHz (Q.ONE INSTRUMENTS, Wuhan, China), Bruker AVANCEIII400 MHz (Bruker, Karlsruhe, Germany) spectrometer in CDCl_3_ using a solvent peak as the standard. Mass spectral analyses were performed with Thermo Scientific Q Exactive™ Plus (Thermo Scientific, Waltham, MA, USA). Flash column chromatography was performed on Qingdao Haiyang Chemical Co., Ltd. (Yantai, China) silica gel 60 (200–300 mesh). Analytical TLC was performed on Yantai Chemical Industry Research Institute (Yantai, China) silica gel 60 F254 plates, and the rotavapor was EYELA Rotavapor N-1300D-WB (EYELA, Tokyo, Japan).

### 3.2. General Procedure for N-Methyl-Benzanilides Preparation

A mixture of corresponding benzoic acid (5 mmol), EDCI (5.5 mmol), HOBT (5.5 mmol), and DMAP (0.5 mmol) in DMF (30 mL) was stirred for 2 min at room temperature in a round bottom flask. Then, Et3N (15 mmol) was added. After 5 minutes, *N*-methylaniline (4.5 mmol) was slowly added. The reaction mixture was stirred at room temperature for 12 h. The reaction was quenched with water, and the mixture was washed once with saturated aqueous NaCl and extracted with EtOAc. The organic layer was dried with anhydrous Na_2_SO_4_ and evaporated in vacuum to give the crude product, which was then purified by silica gel column chromatography to give *N*-methylbenzanilide.

### 3.3. General Procedure for Palladium-Catalyzed Bromination of Benzanilides

To a 15 mL sealed-tube were added benzanilide (0.10 mmol, 1.0 equiv), Na_2_S_2_O_8_ (0.12 mmol, 1.2 equiv), NBS (1.2 mmol, 1.2 equiv), Pd(OAc)_2_ (0.01 mmol, 0.1 equiv), and TFAA (0.1 mL). The reaction system was stirred at room temperature for 2 min. Then, TFA (0.9 mL) was added. The tube was sealed and heated. After completion of the reaction as determined by TLC (DCM:MeOH = 300:1), the reaction solution was allowed to cool to room temperature. Saturated aqueous NaHCO_3_ was added to the reaction solution and washed with dichloromethane. Then the organic phase was separated and dried over anhydrous Na_2_SO_4_. After removing the solvent in vacuo, the residue was purified by silica gel column chromatography to give the desired product.

### 3.4. General Procedure for HFIP-Catalyzed Bromination of Benzanilides

To a 15 mL sealed-tube were added benzanilide (0.10 mmol, 1.0 equiv), NBS (0.12 mmol, 1.2 equiv), TFAA (0.12 mmol, 1.2 equiv), and HFIP (1 mL). The tube was sealed and heated. After completion of the reaction as determined by TLC (DCM:MeOH = 300:1), the reaction solution was allowed to cool to room temperature. Saturated aqueous NaHCO_3_ was added to the reaction solution and washed with dichloromethane. Then the organic layer phase was separated and was dried over anhydrous Na_2_SO_4_. After removing the solvent in vacuo, the residue was purified by silica gel column chromatography to give the desired product.

### 3.5. Procedure for Gram Scale Reaction

#### 3.5.1. *N*-(2-Bromophenyl)-4-Iodo-*N*-Methylbenzamide

To a 100 mL round bottom flask, following the general procedure *3.3*, 4-iodo-*N*-methyl-*N*-phenylbenzamide (1.0 g, 2.97 mmol), Na_2_S_2_O_8_ (858 mg, 3.56 mmol), NBS (634 mg, 3.56 mmol), Pd(OAc)_2_ (33 mg, 0.15 mmol), TFAA (3 mL), and TFA(27 mL) were added. The reaction mixture was stirred at 50 °C for 3 h. After completion of the reaction as determined by TLC, the residue was purified by silica gel column chromatography with a mixture DCM and MeOH as the eluent. (DCM:MeOH = 900:1).

#### 3.5.2. *N*-(4-Bromophenyl)-4-Iodo-*N*-Methylbenzamide

To a 75 mL sealed tube, following the general procedure *3.4*, 4-iodo-*N*-methyl-*N*-phenylbenzamide (1.0 g, 2.97 mmol) NBS (633.5 mg, 3.56 mmol), and TFAA (504.5 μL, 3.56 mmol) were used, with HFIP (25 mL) as the solvent. The reaction mixture was stirred at 85 °C in the sealed tube for 5 h. After completion of the reaction as determined by TLC, the residue was purified by silica gel column chromatography with a mixture of DCM and MeOH as the eluent. (DCM:MeOH = 900:1).

### 3.6. Characterization of Representative Substrates in Details

**3-bromo-*N*-methyl-*N*-phenylbenzamide**. Following the general procedure *3.2*, 3-bromo-*N*-methyl-*N*-phenylbenzamide was obtained by column chromatography on silica gel (eluent: petroleum ether/ethyl acetate = 20:1). ^1^H NMR (500 MHz, CDCl_3_) δ (ppm) 6.77 (t, *J* = 1.8 Hz, 1H), 6.62 (ddd, *J* = 8.0, 1.9, 1.0 Hz, 1H), 6.52 (td, *J* = 7.0, 1.6 Hz, 2H), 6.46–6.42 (m, 1H), 6.40 (d, *J* = 7.8 Hz, 1H), 6.32–6.24 (m, 3H), 2.75 (s, 1H). ^13^C NMR (125 MHz, CDCl_3_) δ (ppm) 168.3, 143.7, 137.2, 132.0, 131.2, 128.7, 128.6, 126.5, 126.3, 126.3, 121.3, 37.8. ^13^C NMR data were derived from a JEOL-JNM-ECZ500R 500 MHz. HRMS (ESI) calcd for C_14_H_12_BrNO [M+H]^+^: 290.0102, found 290,0206.

**3-fluoro-*N*-methyl-*N*-phenylbenzamide**. Following the general procedure *3.2*, 3-fluoro-*N*-methyl-*N*-phenylbenzamide was obtained by column chromatography on silica gel(eluent: petroleum ether/ethyl acetate = 20:1). ^1^H NMR (500 MHz, CDCl_3_) δ (ppm) 7.26–7.22 (m, 2H), 7.17 (dt, *J* = 8.3, 1.6 Hz, 1H), 7.14–7.10 (m, 1H), 7.03 (dt, *J* = 6.7, 3.4 Hz, 4H), 6.95–6.90 (m, 1H), 3.49 (s, 3H). ^13^C NMR (125 MHz, CDCl_3_) δ (ppm) 167.8, 160.6 (d, *J*_C-F_ = 246.9 Hz), 143.0, 136.6 (d, *J*_C-F_ = 7.1 Hz), 127.9 (d, *J*_C-F_ = 7.9 Hz), 127.9, 125.5, 125.4, 123.0 (d, *J*_C-F_ = 3.6 Hz), 115.3 (d, *J*_C-F_ = 21.2 Hz), 114.3 (d, *J*_C-F_ = 23.2 Hz), 37.0. ^13^C NMR data were derived from a JEOL-JNM-ECZ500R 500 MHz. HRMS (ESI) calcd for C_14_H_12_FNO [M+H]^+^: 230.0981, found 230.0998.

**2-fluoro-*N*-methyl-*N*-phenylbenzamide**. Following the general procedure *3.2*, 2-fluoro-*N*-methyl-*N*-phenylbenzamide was obtained by column chromatography on silica gel (eluent: petroleum ether/ethyl acetate = 20:1). ^1^H NMR (500 MHz, CDCl_3_) δ (ppm) 7.33–7.27 (m, 1H), 7.23–7.14 (m, 3H), 7.15–7.09 (m, 1H), 7.08–7.02 (m, 2H), 6.98 (t, *J* = 7.7 Hz, 1H), 6.79 (t, *J* = 9.1 Hz, 1H), 3.48 (s, 3H). ^13^C NMR (125 MHz, CDCl_3_) δ (ppm) 165.7, 157.0 (d, *J*_C-F_ = 248.9 Hz), 142.5, 130.1 (d, *J*_C-F_ = 9.3 Hz), 128.4 (d, *J*_C-F_ = 4.6 Hz), 127.9, 126.1, 125.9, 124.3 (d, *J*_C-F_ = 18.3 Hz), 122.9, 114.5 (d, *J*_C-F_ = 22.7 Hz), 36.5. ^13^C NMR data were derived from a JEOL-JNM-ECZ500R 500 MHz. HRMS (ESI) calcd for C_14_H_12_FNO [M+H]^+^: 230.0981, found 230.0997.

**4-iodo-*N*-methyl-*N*-phenylbenzamide**. Following the general procedure *3.2*, 4-iodo-*N*-methyl-*N*-phenylbenzamide was obtained by column chromatography on silica gel (eluent: petroleum ether/ethyl acetate = 15:1). ^1^H NMR (500 MHz, CDCl_3_) δ (ppm) 7.49 (dt, *J* = 8.8, 2.3, 1.8 Hz, 2H), 7.26–7.21 (m, 2H), 7.18–7.13 (m, 1H), 7.00 (d, *J* = 8.1 Hz, 4H), 3.47(s, 3H). ^13^C NMR (125 MHz, CDCl_3_) δ (ppm) 169.7, 144.7, 137.0, 135.4, 130.5, 129.5, 126.9, 126.9, 96.4, 38.6. ^13^C NMR data were derived from a JEOL-JNM-ECZ500R 500 MHz. HRMS (ESI) calcd for C_14_H_12_INO [M+H]^+^: 338.0042, found 338.0038.

**4-chloro-*N*-methyl-*N*-phenylbenzamide**. Following the general procedure *3.2*, 4-chloro-*N*-methyl-*N*-phenylbenzamide was obtained by column chromatography on silica gel (eluent: petroleum ether/ethyl acetate = 20:1). ^1^H NMR (500 MHz, CDCl_3_) δ (ppm) δ7.26–7.19 (m, 4H), 7.16 (d, *J* = 7.0 Hz, 1H), 7.12 (d, *J* = 8.4 Hz, 2H), 7.01 (d, *J* = 7.9 Hz, 2H), 3.47(s, 3H). ^13^C NMR (125 MHz, CDCl_3_) δ (ppm) 169.6, 144.8, 135.8, 134.4, 130.3, 129.4, 128.1, 127.0, 126.9, 38.6. ^13^C NMR data were derived from a JEOL-JNM-ECZ500R 500 MHz. HRMS (ESI) calcd for C_14_H_12_ClNO [M+H]^+^: 246.0686, found 246.0680.

**4-fluoro-*N*-methyl-*N*-phenylbenzamide**. Following the general procedure *3.2*, 4-fluoro-*N*-methyl-*N*-phenylbenzamide was obtained by column chromatography on silica gel (eluent: petroleum ether/ethyl acetate = 20:1). ^1^H NMR (500 MHz, CDCl_3_) δ (ppm) 7.95–7.04 (m, 6H), 7.11–7.07 (m, 1H), 6.96 (d, *J* = 7.4 Hz, 1H), 6.89–6.62 (m, 1H), 3.40 (s, 3H). ^13^C NMR (125 MHz, CDCl_3_) δ (ppm) 168.3, 161.9 (d, *J*_C-F_ = 248.4 Hz), 143.6, 130.7, 129.8 (d, *J*_C-F_ = 10.8 Hz), 128.0, 125.6, 125.4, 113.5 (d, *J*_C-F_ = 23.1 Hz), 37.2. ^13^C NMR data were derived from a JEOL-JNM-ECZ500R 500 MHz. HRMS (ESI) calcd for C_14_H_12_FNO [M+H]^+^: 230.0981, found 230.0973.

***N*-methyl-*N*-phenyl-4-(trifluoromethyl)benzamide**. Following the general procedure *3.2*, 4-fluoro-*N*-methyl-*N*-phenylbenzamide was obtained by column chromatography on silica gel (eluent: petroleum ether/ethyl acetate = 10:1). ^1^H NMR (500 MHz, CDCl_3_) δ (ppm) δ 7.66–7.31 (m, 5H), 7.30–7.09 (m, 3H), 7.07–6.93 (m, 1H), 3.45(s, 3H) ^13^C NMR (125 MHz, CDCl_3_) δ (ppm) 167.9, 142.9, 138.1, 130.1 (q, *J*_C-F_ = 32.9 Hz), 128.1, 127.7, 125.7, 125.6, 123.5 (q, *J*_C-F_ = 3.9 Hz), 116.7, 37.1. ^13^C NMR data were derived from a JEOL-JNM-ECZ500R 500 MHz. HRMS (ESI) calcd for C_15_H_12_F_3_NO [M+H]^+^: 280.0949, found 280.0940.

***N*-methyl-4-nitro-*N*-phenylbenzamide**. Following the general procedure *3.2*, 4-fluoro-*N*-methyl-*N*-phenylbenzamide was obtained by column chromatography on silica gel (eluent: petroleum ether/ethyl acetate = 5:1). ^1^H NMR (500 MHz, CDCl_3_) δ (ppm) 7.99 (d, *J* = 8.7 Hz, 2H), 7.41 (d, *J* = 8.7 Hz, 2H), 7.21 (d, *J* = 7.9 Hz, 2H), 7.17–7.13 (m, 1H), 6.99 (d, *J* = 7.6 Hz, 2H), 3.49 (s, 3H). ^13^C NMR (125 MHz, CDCl_3_) δ (ppm) 167.7, 147.3, 143.1, 141.4, 128.9, 128.9, 126.7, 126.3, 122.3, 37.6. ^13^C NMR data were derived from a JEOL-JNM-ECZ500R 500 MHz. HRMS (ESI) calcd for C_14_H_12_N_2_O_3_ [M+H]^+^: 257.0926, found 257.0918.

### 3.7. Characterization of Products in Details

***N*-(2-Bromophenyl)-*N*-methylbenzamide (2a)**. Following the general procedure *3.3*, *N*-methyl-*N*-phenylbenzamide (21.1 mg, 0.1 mmol), NBS (21.4 mg, 0.12 mmol), Na_2_S_2_O_8_ (28.6 mg, 0.12 mmol), and Pd(OAc)_2_ (10 mol%, 0.01 mmol) were used with TFA:TFAA = 0.9 mL:0.1 mL as solvent. The reaction system was stirred at 70 °C in a sealed tube for 1.5 h. After the reaction was completed, the residue was purified by silica gel column chromatography to give **2a** in 60% yield. ^1^H NMR (500 MHz, CDCl_3_) δ (ppm) 7.53 (d, *J* = 7.9 Hz, 1H), 7.34 (d, *J* = 7.3 Hz, 2H), 7.21 (d, *J* = 7.4 Hz, 1H), 7.14 (d, *J* = 7.2 Hz, 3H), 7.09–7.05 (m, 2H), 3.39 (s, 3H). ^13^C NMR (125 MHz, CDCl_3_) δ (ppm) 171.1, 143.8, 133.8, 130.8, 129.9, 129.1, 128.5, 128.2, 127.7, 127.0, 122.8, 37.2. ^13^C NMR data were derived from a JEOL-JNM-ECZ500R 500 MHz. HRMS (ESI) calcd for C_14_H_12_BrNO [M+H]^+^: 290.0102, found 290,0176.

***N*-(2-Bromophenyl)-*N*-methyl-4-(trifluoromethyl)benzamide (2b)**. Following the general procedure *3.3*, *N*-methyl-*N*-phenyl-4-(trifluoromethyl)benzamide (28 mg, 0.1 mmol), NBS (21 mg, 0.12 mmol), Na_2_S_2_O_8_ (28.6 mg, 0.12 mmol), and Pd(OAc)_2_ (10 mol%, 0.01 mmol) were used with TFA:TFAA = 0.9 mL:0.1 mL as the solvent. The reaction system was stirred at room temperature in a sealed tube for 12 h. After the reaction was completed, the residue was purified by silica gel column chromatography to give **2b** in 63% yield.^1^H NMR (500 MHz, CDCl_3_) δ (ppm) 7.56–7.52 (m, 1H), 7.46 (d, *J* = 8.2 Hz, 2H), 7.42 (d, *J* = 8.3 Hz, 2H), 7.22–7.16 (m, 1H), 7.13–7.07 (m, 2H), 3.40 (s, 3H). ^13^C NMR (125 MHz, CDCl_3_) δ (ppm) 169.6, 143.0, 139.3, 134.0, 132.7, 131.7 (q, *J*_C-F_ = 37.5 Hz), 130.6, 129.6, 128.8, 128.4, 124.8 (q, *J*_C-F_ = 3.9 Hz), 122.8, 37.2. ^13^C NMR data were derived from a JEOL-JNM-ECZ500R 500 MHz. HRMS (ESI) calcd for C_15_H_11_BrF_3_NO [M+H]^+^: 357.9976, found 358.0049.

***N*-(2-bromophenyl)-4-fluoro-*N*-methylbenzamide (2c)**. Following the general procedure *3.3*, 4-fluoro-*N*-methyl-*N*-phenylbenzamide (22.9 mg, 0.1 mmol), NBS (21 mg, 0.12 mmol), Na_2_S_2_O_8_ (28.6 mg, 0.12 mmol), and Pd(OAc)_2_ (10 mol%, 0.01 mmol) were used with TFA:TFAA = 0.9 mL:0.1 mL as the solvent. The reaction system was stirred at room temperature in the sealed tube for 12 h. After the reaction was completed, the residue was purified by silica gel column chromatography to give **2c** in 70% yield. ^1^H-NMR (500 MHz, CDCl_3_) δ (ppm) 7.59–7.50 (m, 1H), 7.35 (dd, *J* = 8.6, 5.4 Hz, 2H), 7.18 (d, *J* = 6.8 Hz, 1H), 7.09 (dd, *J* = 6.7, 4.1 Hz, 2H), 6.83 (t, *J* = 8.5 Hz, 2H), 3.37 (s, 3H). ^13^C NMR (125 MHz, CDCl_3_) δ (ppm) 170.0, 164.4 (d, *J*_C-F_ = 251.0 Hz), 143.7, 133.9, 131.8 (d, *J*_C-F_ = 3.6 Hz), 130.6 (d, *J*_C-F_ = 5.2 Hz), 130.5, 129.3, 128.7, 122.8, 114.9 (d, *J*_C-F_ = 22.0 Hz), 37.3. ^13^C NMR data were derived from a JEOL-JNM-ECZ500R 500 MHz. HRMS (ESI) calcd for C_14_H_11_BrFNO [M+H]^+^: 308.0008, found 308.0081.

***N*-(2-Bromophenyl)-4-chloro-*N*-methylbenzamide(2d)**. Following the general procedure *3.3*, 4-chloro-*N*-methyl-*N*-phenylbenzamide (24.6 mg, 0.1 mmol), NBS (21 mg, 0.12 mmol), Na_2_S_2_O_8_ (28.6 mg, 0.12 mmol) and Pd(OAc)_2_ (10 mol%, 0.01 mmol) were used with TFA:TFAA = 0.9 mL:0.1 mL as solvent. The reaction system was stirred at 50 °C in the sealed tube for 3 h. After the reaction was completed, the residue was purified by silica gel column chromatography to give **2d** in 95% yield. ^1^H-NMR (400 MHz, CDCl_3_) δ (ppm) 7.59 (d, *J* = 7.9 Hz, 1H), 7.36–7.28 (m, 2H), 7.23 (d, *J* = 6.3 Hz, 1H), 7.20–7.06 (m, 4H), 3.42 (s, 3H). ^13^C NMR (150 MHz, CDCl_3_) δ (ppm) 169. 9, 143.5, 133.9, 132.6, 130.6, 129.7, 129.3, 128.7, 128.3, 128.0, 122.8, 37.2. ^13^C NMR data were derived from a JEOL-JNM-ECZ500R 500 MHz. HRMS (ESI) calcd for C_14_H_11_BrClNO [M+H]^+^: 323.9713, found 323.9796.

**4-Bromo-*N*-(2-bromophenyl)-*N*-methylbenzamide (2e)**. Following the general procedure *3.3*, 4-bromo-*N*-methyl-*N*-phenylbenzamide (29.0 mg, 0.1 mmol), NBS (21 mg, 0.12 mmol), Na_2_S_2_O_8_ (28.6 mg, 0.12 mmol) and Pd(OAc)_2_ (10 mol%, 0.01 mmol) were used with TFA:TFAA = 0.9 mL:0.1 mL as solvent. The reaction system was stirred at 60 °C in the sealed tube for 2 h. After the reaction was completed, the residue was purified by silica gel column chromatography to give **2e** in 79% yield. ^1^H-NMR (500 MHz, CDCl_3_) δ (ppm) 7.57–7.49 (m, 1H), 7.28 (d, *J* = 8.4 Hz, 2H), 7.23–7.15 (m, 3H), 7.09 (t, *J* = 7.3 Hz, 2H), 3.36 (s, 3H). ^13^C NMR (125 MHz, CDCl_3_) δ (ppm) 170.0, 143.4, 135.2, 134.6, 134.0, 131.0, 130.6, 129.9, 129.4, 128.7, 124.4, 37.3. ^13^C NMR data were derived from a JEOL-JNM-ECZ500R 500 MHz. HRMS (ESI) calcd for C_14_H_11_Br_2_NO [M+H]^+^: 367.9207, found 367.9289.

***N*-(2-Bromophenyl)-4-iodo-*N*-methylbenzamide (2f)**. Following the general procedure *3.3*, 4-iodo-*N*-methyl-*N*-phenylbenzamide (33.7 mg, 0.1 mmol), NBS (21 mg, 0.12 mmol), Na_2_S_2_O_8_ (28.6 mg, 0.12 mmol) and Pd(OAc)_2_ (10 mol%, 0.01 mmol) were used with TFA:TFAA = 0.9 mL:0.1 mL as solvent. The reaction system was stirred at 50 °C in the sealed tube for 3 h. After the reaction was completed, the residue was purified by silica gel column chromatography to give **2f** in 81% yield. ^1^H-NMR (400 MHz, CDCl_3_) δ (ppm) 7.55 (d, *J* = 7.9 Hz, 1H), 7.50 (d, *J* = 8.0 Hz, 2H), 7.20 (t, *J* = 7.7 Hz, 1H), 7.15–7.03 (m, 4H), 3.37 (s, 3H). ^13^C NMR (150 MHz, CDCl_3_) δ (ppm) 170.1, 143.4, 136.9, 135.2, 133.9, 130.6, 129.8, 129.4, 128.7, 122.8, 96.5, 37.3. ^13^C NMR data were derived from JNM-ECZL600G 600 MHz. HRMS (ESI) calcd for C_14_H_11_BrINO [M+H]^+^: 415.9069, found 415.9142.

***N*-(2-Bromophenyl)-3-fluoro-*N*-methylbenzamide (2g)**. Following the general procedure *3.3*, 3-fluoro-*N*-methyl-*N*-phenylbenzamide (22.9 mg, 0.1 mmol), NBS (26.7 mg, 0.15 mmol), Na_2_S_2_O_8_ (35.7 mg, 0.15 mmol) and Pd(OAc)_2_ (10 mol%, 0.01 mmol) were used with TFA:TFAA = 0.9 mL:0.1 mL as solvent. The reaction system was stirred at 30 °C in the sealed tube for 12 h. After the reaction was completed, the residue was purified by silica gel column chromatography to give **2g** in 76% yield. ^1^H-NMR (400 MHz, CDCl_3_) δ (ppm) 7.55 (d, *J* = 8.1 Hz, 1H), 7.17 (t, *J* = 8.5 Hz, 2H), 7.14–7.08 (m, 4H), 6.93 (d, *J* = 8.8 Hz, 1H), 3.39 (s, 3H). ^13^C NMR (150 MHz, CDCl_3_) δ (ppm) 169.6, 162.0 (d, *J*_C-F_ = 246.0 Hz), 143.3, 137.9 (d, *J*_C-F_ = 7.3 Hz), 133.9, 130.6, 129.4 (d, *J*_C-F_ = 4.0 Hz), 128.6, 126.9, 123.8, 122.8, 117.0 (d, *J*_C-F_ = 21.4 Hz), 115.4 (d, *J*_C-F_ = 22.8 Hz), 37.2. ^13^C NMR data were derived from JNM-ECZL600G 600 MHz. HRMS (ESI) calcd for C_14_H_11_BrFNO [M+H]^+^: 308.0086, found 308.0085.

**3-Bromo-*N*-(2-bromophenyl)-*N*-methylbenzamide (2h)**. Following the general procedure *3.3*, 3-bromo-*N*-methyl-*N*-phenylbenzamide (29.0 mg, 0.1 mmol), NBS (21 mg, 0.12 mmol), Na_2_S_2_O_8_ (28.6 mg, 0.12 mmol) and Pd(OAc)_2_ (10 mol%, 0.01 mmol) were used with TFA:TFAA = 0.9 mL:0.1 mL as solvent. The reaction system was stirred at 50 °C in the sealed tube for 3 h. After the reaction was completed, the residue was purified by silica gel column chromatography to give **2h** in 78% yield. ^1^H-NMR (400 MHz, CDCl_3_) δ (ppm) 7.55 (d, *J* = 6.2 Hz, 2H), 7.34 (d, *J* = 8.1 Hz, 1H), 7.20 (t, *J* = 7.3 Hz, 2H), 7.11 (d, *J* = 7.7 Hz, 2H), 7.00 (t, *J* = 8.0 Hz, 1H), 3.37 (s, 3H). ^13^C NMR (150 MHz, CDCl_3_) δ (ppm) 169.3, 143.2, 137.6, 133.9, 132.9, 131.4, 130.6, 129.4, 129.2, 128.7, 126.5, 122.8, 121.8, 37.2. ^13^C NMR data were derived from JNM-ECZL600G 600 MHz. HRMS (ESI) calcd for C_14_H_11_Br_2_NO [M+H]^+^: 367.9207, found 367.9287.

***N*-(2-Bromophenyl)-2-fluoro-*N*-methylbenzamide (2i)**. Following the general procedure *3.3*, 2-fluoro-*N*-methyl-*N*-phenylbenzamide (22.9 mg, 0.1 mmol), NBS (21 mg, 0.12 mmol), Na_2_S_2_O_8_ (28.6 mg, 0.12 mmol) and Pd(OAc)_2_ (10 mol%, 0.01 mmol) were used with TFA:TFAA = 0.9 mL:0.1 mL as solvent. The reaction system was stirred at room temperature in the sealed tube for 12 h. After the reaction was completed, the residue was purified by silica gel column chromatography to give **2i** in 86% yield. ^1^H-NMR (400 MHz, CDCl_3_) δ (ppm) 7.49 (d, *J* = 8.1 Hz, 1H), 7.38 (t, *J* = 7.3 Hz, 1H), 7.29 (d, *J* = 8.0 Hz, 1H), 7.22–7.13 (m, 2H), 7.06 (t, *J* = 7.7 Hz, 1H), 6.96 (t, *J* = 7.5 Hz, 1H), 6.85 (t, *J* = 9.0 Hz, 1H), 3.41 (s, 3H). ^13^C NMR (150 MHz, CDCl_3_) δ (ppm) 166.7, 158.2 (d, *J*_C-F_ = 249.0 Hz), 157.3, 142.2, 133.5, 131.2 (d, *J*_C-F_ = 8.2 Hz), 130.2 (d, *J*_C-F_ = 2.6 Hz), 129.6, 128.5 (d, *J*_C-F_ = 3.6 Hz), 128.3, 125.0 (d, *J*_C-F_ = 17.1 Hz), 123.7 (d, *J*_C-F_ = 3.5 Hz), 123.0, 115.5 (d, *J*_C-F_ = 21.6 Hz), 36.4. ^13^C NMR data were derived from JNM-ECZL600G 600 MHz. HRMS (ESI) calcd for C_14_H_11_BrFNO [M+H]^+^: 308.0008, found 308.0085.

***N*-(2-Bromophenyl)-2-chloro-*N*-methylacetamide (2j)**. Following the general procedure *3.3*, 2-chloro-*N*-methyl-*N*-phenylacetamide (18.4 mg, 0.1 mmol), NBS (23.4 mg, 0.13 mmol), Na_2_S_2_O_8_ (31 mg, 0.13 mmol) and Pd(OAc)_2_ (10 mol%, 0.01 mmol) were used with TFA:TFAA = 0.9 mL:0.1 mL as solvent. The reaction system was stirred at 80 °C in the sealed tube for 2 h. After the reaction was completed, the residue was purified by silica gel column chromatography to give **2j** in 54% yield. ^1^H-NMR (400 MHz, CDCl_3_) δ (ppm) 7.80 (d, *J* = 8.0 Hz, 1H), 7.53 (t, *J* = 7.5 Hz, 1H), 7.46 (d, *J* = 7.6 Hz, 1H), 7.43–7.35 (m, 1H), 3.87 (q, *J* = 13.3 Hz, 2H), 3.34 (s, 3H). ^13^C NMR (150 MHz, CDCl_3_) δ(ppm) 166.2, 141.3, 134.2, 130.6, 130.0, 129.3, 123.2, 41.8, 36.7. ^13^C NMR data were derived from JNM-ECZL600G 600 MHz. HRMS (ESI) calcd for C_9_H_9_BrClNO [M+H]^+^: 261.9556, found 261.9637.

***N*-(2-bromophenyl)-*N*-methyl-4-nitrobenzamide (2k)** Following the general proce-dure *3.3*, *N*-methyl-4-nitro-*N*-phenylbenzamide (25.6 mg, 0.1 mmol), NBS (21.4 mg, 1.2 mmol), Na_2_S_2_O_8_ (28.6 mg, 1.2 mmol) and Pd(OAc)_2_ (10 mol%, 0.01 mmol) were used with TFA:TFAA = 0.9 mL:0.1 mL as solvent. The reaction system was stirred at 70 °C in the sealed tube for 3 h. After the reaction was completed, the residue was purified by silica gel column chromatography to give 2l in 53% yield. ^1^H-NMR (700 MHz, CDCl_3_) δ (ppm) 8.03 (d, *J* = 8.5 Hz, 2H), 7.55 (d, *J* = 8.0 Hz, 1H), 7.52 (d, *J* = 8.5 Hz, 2H), 7.22 (t, *J* = 7.6 Hz, 1H), 7.18–7.09 (m, 2H), 3.42 (s, 3H). ^13^C NMR (175 MHz, CDCl_3_) δ(ppm) 168.8, 148.2, 142.5, 141.8, 134.1, 130.5, 129.9, 129.0, 128.9, 123.0, 122.8, 37.1. ^13^C NMR data were derived from Bruker AVANCE NEO 700 MHz. HRMS (ESI) calcd for C_14_H_11_BrN_2_O_3_ [M+H]^+^:335.0031, found 335.0040.

***N*-(4-Bromophenyl)-*N*-methylbenzamide (3a)**. Following the general procedure *3.4*, *N*-methyl-*N*-phenylbenzamide (21.1 mg, 0.1 mmol), NBS (21.4 mg, 0.12 mmol), and TFAA (28 μL, 0.2 mmol) were used with HFIP (1 mL) as the solvent. The reaction system was stirred at 70 °C in the sealed tube for 2 h. After the reaction was completed, the residue was purified by silica gel column chromatography to give **3a** in 73% yield. ^1^H NMR (400 MHz, CDCl_3_) δ (ppm) 7.38 (d, *J* = 8.7 Hz, 2H), 7.35–7.28 (m, 3H), 7.24 (d, *J* = 7.4 Hz, 2H), 6.98–6.90 (m, 2H), 3.50 (s, 3H). ^13^C NMR (125 MHz, CDCl_3_) δ(ppm) 170.6, 144.1, 135.6, 132.4, 130.0, 128.7, 128.5, 128.0, 120.0, 38.4. ^13^C NMR data were derived from a JEOL-JNM-ECZ500R 500 MHz. HRMS (ESI) calcd for C_14_H_12_BrNO [M+H]^+^: 290.0102, found 290.0179.

***N*-(4-Bromophenyl)-*N*-methyl-4-(trifluoromethyl)benzamide (3b)**. Following the general procedure *3.4*, *N*-methyl-*N*-phenyl-4-(trifluoromethyl)benzamide (35.8 mg, 0.1 mmol), NBS (19.6 mg, 0.11 mmol), and TFAA (25 μL, 0.18 mmol) were used with HFIP (1 mL) as the solvent. The reaction system was stirred at 100 °C in the sealed tube for 2 h. After the reaction was completed, the residue was purified by silica gel column chromatography to give **3b** in 82% yield. ^1^H NMR (500 MHz, CDCl_3_) δ (ppm) 7.46 (d, *J* = 8.2 Hz, 2H), 7.40–7.34 (m, 4H), 6.90 (d, *J* = 8.5 Hz, 2H), 3.46 (s, 3H). ^13^C NMR (125MHz, CDCl_3_) δ(ppm) 169.1, 143.4, 132.7, 131.6 (q, *J*_C-F_ = 32.7 Hz), 129.6, 129.0, 128.5, 125.1 (q, *J*_C-F_ = 4.0 Hz), 122.6, 120.7, 38.5. ^13^C NMR data were derived from a JEOL-JNM-ECZ500R 500 MHz. HRMS (ESI) calcd for C_15_H_11_BrF_3_NO[M+H]^+^: 357.9976, found 358.0051.

***N*-(4-Bromophenyl)-4-fluoro-*N*-methylbenzamide (3c)**. Following the general procedure *3.4*, 4-fluoro-*N*-methyl-*N*-phenylbenzamide (22.9 mg, 0.1 mmol), NBS (21.4 mg, 0.12 mmol), and TFAA (28 μL, 0.2 mmol) were used with HFIP (1 mL) as the solvent. The reaction system was stirred at 90 °C in the sealed tube for 2 h. After the reaction was completed, the residue was purified by silica gel column chromatography to give **3c** in 83% yield. ^1^H NMR (400 MHz, CDCl_3_) δ (ppm) 7.40 (d, *J* = 8.5 Hz, 2H), 7.33 (d, *J* = 8.5, 2H), 6.95–6.89 (m, 4H), 3.50 (s, 3H). ^13^C NMR (125 MHz, CDCl_3_) δ(ppm) 169.5, 164.4 169.52, 163.43 (d, *J*_C-F_ = 251.1 Hz), 144.0, 132.6, 131.6 (d, *J*_C-F_ = 3.6 Hz), 131.1 (d, *J*_C-F_ = 8.6 Hz), 128.4, 120.2, 115.1 (d, *J*_C-F_ = 21.8 Hz), 38.5. ^13^C NMR data were derived from a JEOL-JNM-ECZ500R 500 MHz. HRMS (ESI) calcd for C_14_H_11_BrFNO [M+H]^+^: 308.0008, found 308.0088.

***N*-(4-Bromophenyl)-4-chloro-*N*-methylbenzamide (3d)**. Following the general procedure *3.4*, 4-chloro-*N*-methyl-*N*-phenylbenzamide (24.6 mg, 0.1 mmol), NBS (21.4 mg, 0.12 mmol), and TFAA (28 μL, 0.20 mmol) were used with HFIP (1 mL) as the solvent. The reaction system was stirred at 90 °C in the sealed tube for 2 h. After the reaction was completed, the residue was purified by silica gel column chromatography to give **3d** in 79% yield. ^1^H NMR (400 MHz, CDCl_3_) δ (ppm) 7.45–7.37 (m, 2H), 7.34–7.26 (m, 2H), 7.22 (d, *J* = 8.6 Hz, 2H), 6.99–6.88 (m, 2H), 3.50 (s, 3H). ^13^C NMR (150 MHz, CDCl_3_) δ(ppm) 169.4, 143.8, 136.1, 133.9, 132.6, 130.2, 128.4, 128.3, 120.3, 38.5. ^13^C NMR data were derived from JNM-ECZL600G 600 MHz. HRMS (ESI) calcd for C_14_H_11_BrClNO [M+H]^+^: 323.9713, found 323.9788.

**4-Bromo-*N*-(4-bromophenyl)-*N*-methylbenzamide (3e)**. Following the general procedure *3.4*, 4-bromo-*N*-methyl-*N*-phenylbenzamide (29 mg, 0.1 mmol), NBS (21.4 mg, 0.12 mmol), and TFAA (17 μL, 0.12 mmol) were used with HFIP (1 mL) as the solvent. The reaction system was stirred at 85 °C in the sealed tube for 5 h. After the reaction was completed, the residue was purified by silica gel column chromatography to give **3e** in 75% yield. ^1^H NMR (400 MHz, CDCl_3_) δ (ppm) 7.45–7.34 (m, 4H), 7.23–7.16 (m, 2H), 6.97–6.90 (m, 2H), 3.49 (s, 3H). ^13^C NMR (150 MHz, CDCl_3_) δ(ppm) 169.4, 143.8, 134.4, 132.6, 131.3, 130.4, 128.4, 128.4, 124.5, 120.4, 38.4. ^13^C NMR data were derived from JNM-ECZL600G 600 MHz. HRMS (ESI) calcd for C_14_H_11_Br_2_NO [M+H]^+^: 367.9207, found 367.9278.

***N*-(4-Bromophenyl)-4-iodo-*N*-methylbenzamide (3f)**. Following the general procedure *3.4*, 4-iodo-*N*-methyl-*N*-phenylbenzamide (33.7 mg, 0.1 mmol), NBS (21.4 mg, 0.12 mmol), and TFAA (17 μL, 0.12 mmol) were used with HFIP (1 mL) as the solvent. The reaction system was stirred at 85 °C in the sealed tube for 5 h. After the reaction was completed, the residue was purified by silica gel column chromatography to give **3f** in 73% yield. ^1^H NMR (400 MHz, CDCl_3_) δ (ppm) 7.61–7.51 (m, 2H), 7.37 (dd, *J* = 8.5, 1.6 Hz, 2H), 7.07–6.98 (m, 2H), 6.96–6.83 (m, 2H), 3.45 (s, 3H). ^13^C NMR (150 MHz, CDCl_3_) δ (ppm) 169.6, 143.7, 137.2, 135.0, 132.6, 130.4, 128.4, 120.4, 96.7, 38.5. ^13^C NMR data were derived from JNM-ECZL600G 600 MHz. HRMS (ESI) calcd for C_14_H_11_BrINO [M+H]^+^: 415.9069, found 415.9145.

***N*-(4-Bromophenyl)-3-fluoro-*N*-methylbenzamide (3g)**. Following the general procedure *3.4*, 3-fluoro-*N*-methyl-*N*-phenylbenzamide (22.9 mg, 0.1 mmol), NBS (21.4 mg, 0.12 mmol), and TFAA (17 μL, 0.12 mmol) were used with HFIP (1 mL) as the solvent. The reaction system was stirred at 85 °C in the sealed tube for 5 h. After the reaction was completed, the residue was purified by silica gel column chromatography to give **3g** in 86% yield. ^1^H NMR (400 MHz, CDCl_3_) δ (ppm) 7.40 (d, *J* = 8.2 Hz, 2H), 7.30 (s, 1H), 7.25–7.14 (m, 1H), 7.08–6.97 (m, 2H), 6.95 (d, *J* = 8.2 Hz, 2H), 3.49 (s, 3H). ^13^C NMR (150 MHz, CDCl_3_) δ (ppm) 169.1, 162.2 (d, *J*_C-F_ = 247.4 Hz), 143.6, 137.7 (d, *J*_C-F_ = 7.2 Hz), 132.5, 129.7 (d, *J*_C-F_ = 8.1 Hz), 128.4, 124.4 (d, *J*_C-F_ = 3.1 Hz), 120.4, 117.1 (d, *J*_C-F_ = 21.1 Hz), 115.9 (d, *J*_C-F_ = 23.1 Hz), 38.4. ^13^C NMR data were derived from JNM-ECZL600G 600 MHz. HRMS (ESI) calcd for C_14_H_11_BrFNO [M+H]^+^: 308.0086, found 308.0083.

**3-Bromo-*N*-(4-bromophenyl)-*N*-methylbenzamide (3h)**. Following the general procedure *3.4*, *N*-(4-bromophenyl)-*N*-methylbenzamide (29 mg, 0.1 mmol), NBS (21.4 mg, 0.12 mmol), and TFAA (17 μL, 0.12 mmol) were used with HFIP (1 mL) as the solvent. The reaction system was stirred at 85 °C in the sealed tube for 5 h. After the reaction was completed, the residue was purified by silica gel column chromatography to give **3h** in 96% yield. ^1^H NMR (400 MHz, CDCl_3_) δ (ppm) 7.53 (s, 1H), 7.45–7.32 (m, 3H), 7.11 (d, *J* = 7.1 Hz, 1H), 7.04 (t, *J* = 7.9 Hz, 1H), 6.92 (d, *J* = 7.4 Hz, 2H), 3.45 (s, 3H). ^13^C NMR (150 MHz, CDCl_3_) δ (ppm) 168.9, 143.5, 137.6, 133.0, 132.6, 131.8, 129.5, 128.4, 127.1, 122.2, 120.5, 38.4. ^13^C NMR data were derived from JNM-ECZL600G 600 MHz. HRMS (ESI) calcd for C_14_H_11_Br_2_NO [M+H]^+^: 367.9207, found 367.9292.

***N*-(4-Bromophenyl)-2-fluoro-*N*-methylbenzamide (3i)**. Following the general procedure *3.4*, 2-fluoro-*N*-methyl-*N*-phenylbenzamide (22.9 mg, 0.1 mmol), NBS (21.4 mg, 0.12 mmol), and TFAA (17 μL, 0.12 mmol) were used with HFIP (1 mL) as the solvent. The reaction system was stirred at 100 °C in the sealed tube for 5 h. After the reaction was completed, the residue was purified by silica gel column chromatography to give **3i** in 79% yield. ^1^H NMR (400 MHz, CDCl_3_) δ (ppm) 7.41–7.19 (m, 4H), 7.10–6.88 (m, 3H), 6.87–6.78 (m, 1H), 3.45 (s, 3H). ^13^C NMR (150 MHz, CDCl_3_) δ (ppm) 166.5, 157.93 (d, *J*_C-F_ = 249.0Hz), 142.6, 132.1, 131.5 (d, *J*_C-F_ = 8.1 Hz), 129.4, 128.5, 124.8 (d, *J*_C-F_ = 17.0 Hz), 124.2, 120.7, 115.8 (d, *J*_C-F_ = 21.6 Hz), 37.5. ^13^C NMR data were derived from JNM-ECZL600G 600 MHz. HRMS (ESI) calcd for C_14_H_11_BrFNO [M+H]^+^: 308.0008, found 308.0084.

***N*-(4-Bromophenyl)-2-chloro-*N*-methylacetamide (3j)**. Following the general procedure *3.4*, 2-chloro-*N*-methyl-*N*-phenylacetamide (18.4 mg, 0.1 mmol), NBS (19.6 mg, 0.11 mmol), and TFAA (17 μL, 0.12 mmol) were used with HFIP (1 mL) as the solvent. The reaction system was stirred at 70 °C in the sealed tube for 2 h. After the reaction was completed, the residue was purified by silica gel column chromatography to give **3j** in 76% yield. ^1^H NMR (700 MHz, CDCl_3_) δ (ppm) 7.59 (s, 1H), 7.58 (s, 1H), 7.15 (d, *J* = 8.6 Hz, 2H), 3.83 (s, 2H), 3.29 (s, 3H) ^13^C NMR (150 MHz, CDCl_3_) δ (ppm) 166.2, 141.8, 133.4, 128.9, 122.5, 41.3, 38.0. ^13^C NMR data were derived from JNM-ECZL600G 600 MHz. HRMS (ESI) calcd for C_9_H_9_BrClNO [M+H]^+^: 261.9556, found 261.9634.

***N*-(4-bromophenyl)-*N*-methyl-4-nitrobenzamide (3k)** Following the general proce-dure *3.3*, *N*-methyl-4-nitro-*N*-phenylbenzamide (25.6 mg, 0.1 mmol), NBS (26.7 mg, 0.15 mmol), and TFAA (21 μL, 0.15 mmol) were used with HFIP (1 mL) as the solvent. The reaction system was stirred at 70 °C in the sealed tube for 2 h. After the reaction was completed, the residue was purified by silica gel column chromatography to give 2l in 82% yield. ^1^H-NMR (700 MHz, CDCl_3_) δ (ppm) 8.07 (d, *J* = 7.9 Hz, 2H), 7.44 (d, *J* = 8.0 Hz, 2H), 7.38 (d, *J* = 7.7 Hz, 2H), 7.26 (d, *J* = 1.6 Hz, 2H), 6.91 (d, *J* = 6.9 Hz, 2H), 3.49 (s, 3H). ^13^C NMR (175 MHz, CDCl_3_) δ(ppm) 168.3, 148.2, 141.7, 132.8, 129.6, 129.0, 128.5, 123.3, 121.0, 38.4. ^13^C NMR data were derived from Bruker AVANCE NEO 700 MHz. HRMS (ESI) calcd for C_14_H_11_BrN_2_O_3_ [M+H]^+^:335.0031, found 335.0036.

## 4. Conclusions

In summary, a novel regioselective modification strategy was developed for the facile synthesis of a broad range of brominated benzanilides from easily accessible starting materials. When Pd(II) was utilized as a catalyst, bromination occured on the *ortho* position of the amino group. In contrast, a reversed site-selective product was obtained via an HFIP-promoted transformation. This method was found to be widely useful for the generation of a broad range of brominated anilides with excellent regioselectivity. These two types of small molecules are widely used in early-stage drug discovery. It was shown that C–H bromination could even be carried out at room temperature. The switchable reactions demonstrated excellent regioselectivity, good functional group tolerance, and high yields. Further studies into the mechanism and applications of this novel strategy are currently underway in our laboratory.

## Figures and Tables

**Table 1:** Optimization of conditions for selective bromination of benzanilide ^a^.

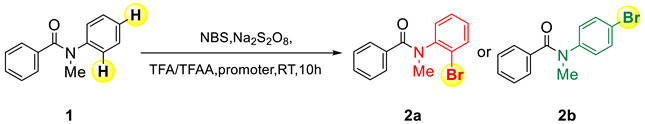			
entry	promoter	**2a**	**2b**
		yield ^b^ (%)	yield ^b^ (%)
1 ^c^	none	<5	<5
2	Pd/C	<5	<5
3	Pd(OAc)_2_	56	<5
4	Pd(TFA)_2_	42	<5
5	PdCl_2_	<5	<5
**6 ^d^**	**Pd(OAc)_2_**	**72**	**<5**
7	Co(acac)_2_	<5	<5
8	Cu(OAc)_2_	<5	<5
9 ^e^	HFIP	<5	68
10	Cp*Co(CO)I_2_	<5	<5
11	Ni(TFA)_2_	<5	<5
**12 ^f^**	**HFIP**	**<5**	**83**

^a^ Reaction conditions: benzanilide (0.1 mmol, 1.0 eq), NBS (1.2 eq), promoter (0.1 eq), Na_2_S_2_O_8_ (1.2 eq), in TFAA (0.1 mL)/TFA (0.9 mL) RT, 10 h. ^b^ Conversion ratio. ^c^ Reaction conditions: benzanilide (0.1 mmol, 1.0 eq), NBS (1.2 eq), Na_2_S_2_O_8_ (1.2 eq), in TFAA (0.1 mL)/TFA (0.9 mL), RT, 10 h. ^d^ Reaction conditions: benzanilide (0.1 mmol, 1.0 eq), NBS (1.2 eq), Na_2_S_2_O_8_ (1.2 eq), in TFAA (0.1 mL)/TFA (0.9 mL), 70 °C, 1 h. ^e^ Reaction conditions: benzanilide (0.1 mmol, 1.0 eq), NBS (1.2 eq), TFAA (2.0 eq) in HFIP (1 mL), RT, 10 h. ^f^ Reaction conditions: benzanilide (0.1 mmol, 1.0 eq), NBS (1.2 eq), TFAA (2.0 eq) in HFIP (1 mL) 70 °C 2 h.

## Data Availability

The data are contained within the article and Supplementary Materials.

## Supplementary Materials

The following supporting information can be downloaded at: https://www.mdpi.com/article/10.3390/molecules29122861/s1, ^1^H, ^13^C-NMR spectral data of all compounds are presented in the Supporting Information file.
