# Supplementary material for: Switchable Site-Selective Benzanilide C(sp2)-H Bromination via Promoter Regulation
Source: Molecules. 2024 Jun 16;29(12):2861. doi: 10.3390/molecules29122861 (PMC11206611; doi:10.3390/molecules29122861)
Supplement: Supplementary file 1 [file molecules-29-02861-s001.zip › molecules-3016801-supplementary.pdf]

Supplementary Information

Switchable Site-Selective Benzanilide C(sp^2)-H Bromination *via* Promoter Regulation

[a] *State Key Laboratory of Bioactive Substance and Function of Natural Medicines, Institute of Materia Medica, Chinese Academy of Medical Sciences and Peking Union Medical College, Beijing 100050, China*

*E-mail addresses: sunyonghui@imm.ac.cn

NMR data of compound *N*-(2-bromophenyl)-*N*-methylbenzamide

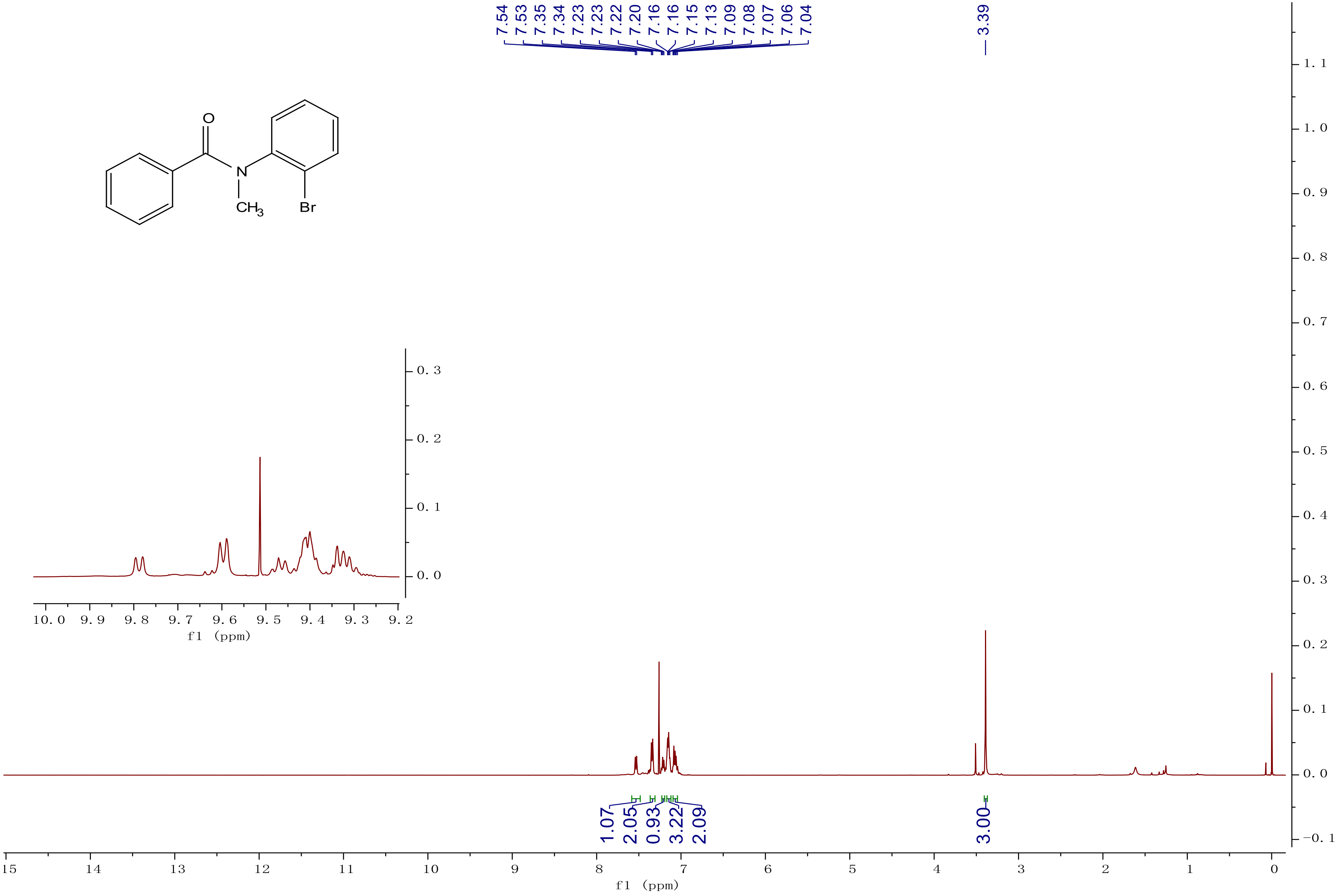

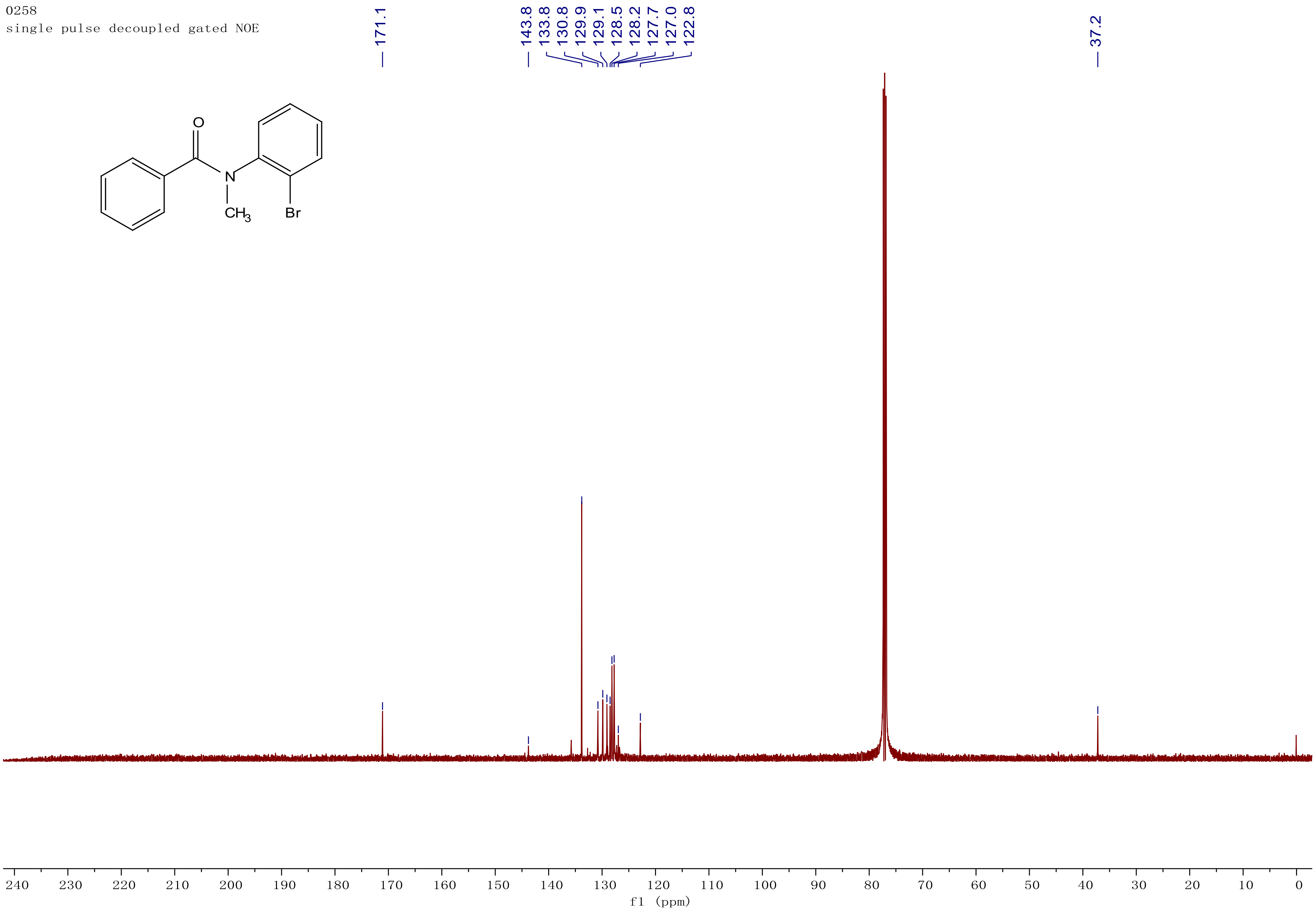
0258
single pulse decoupled gated NOE
171.1
143.8
133.8
130.8
129.9
129.1
128.5
128.2
127.7
127.0
122.8
37.2
O
N
CH3
Br
240 230 220 210 200 190 180 170 160 150 140 130 120 110 100 90 80 70 60 50 40 30 20 10 0
f1 (ppm)

NMR data of compound *N*-(2-bromophenyl)-4-fluoro-*N*-methylbenzamide

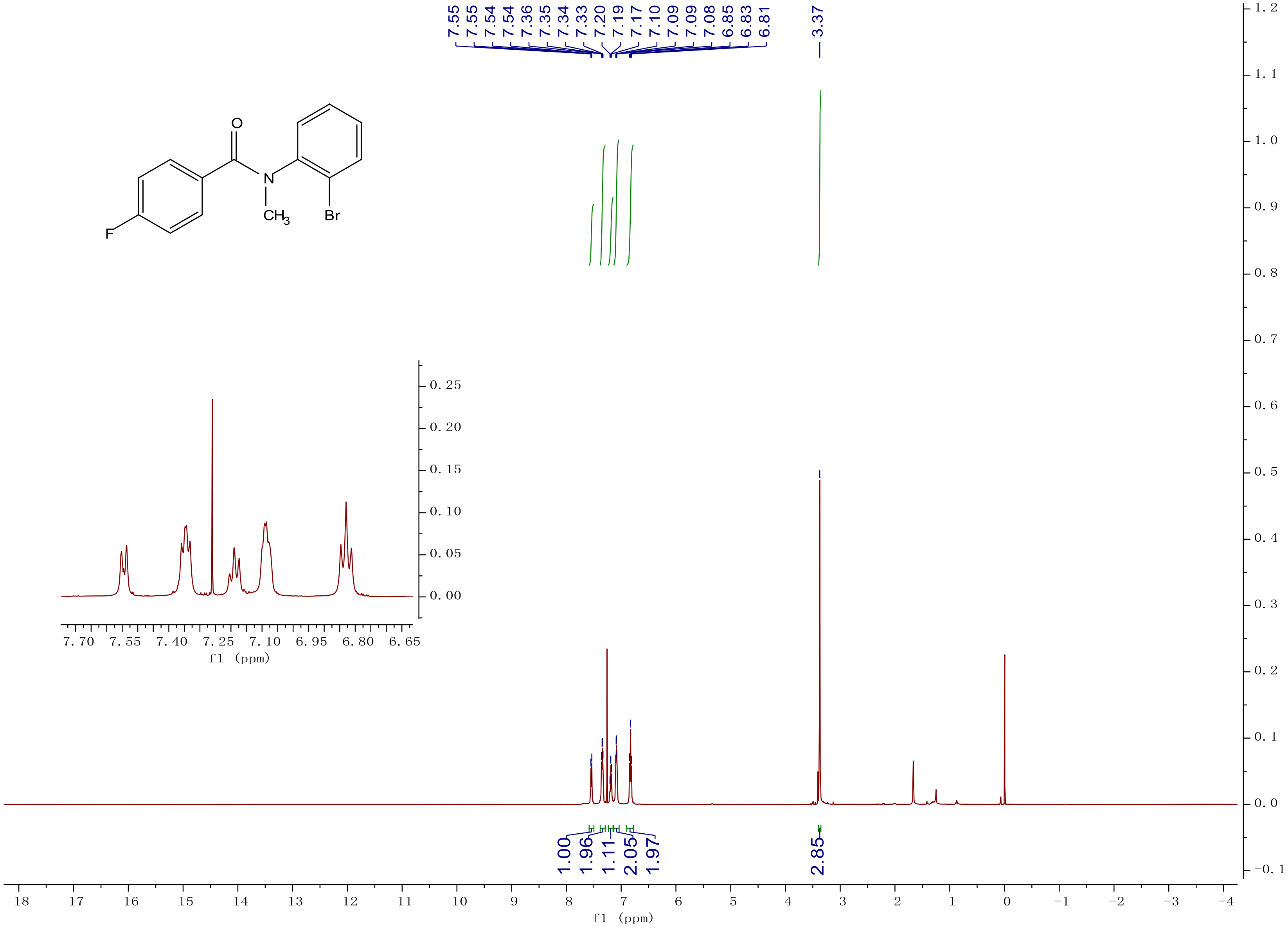

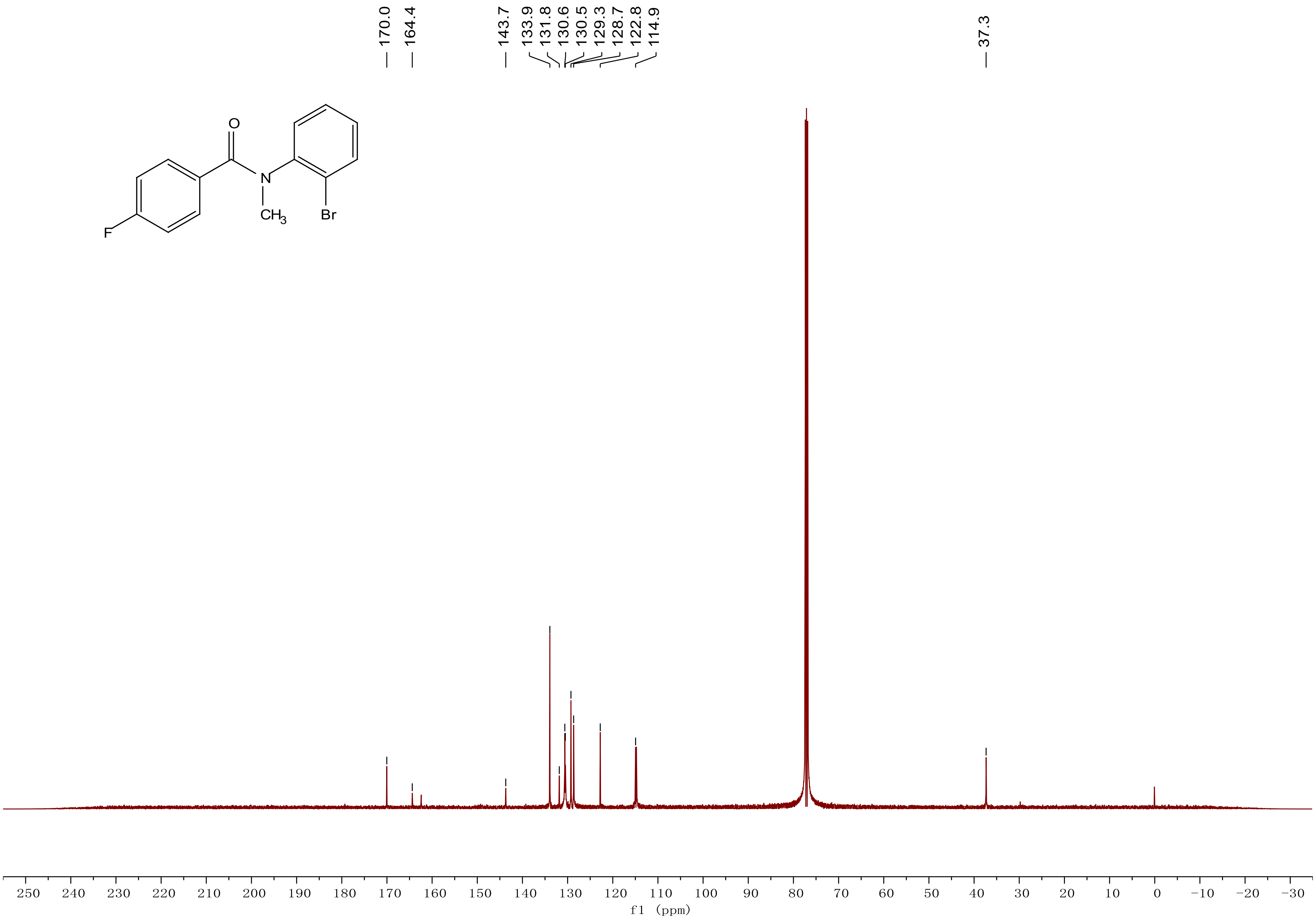
170.0
164.4
143.7
133.9
131.8
130.6
130.5
129.3
128.7
122.8
114.9
37.3
O
N
CH3
Br
F
250 240 230 220 210 200 190 180 170 160 150 140 130 120 110 100 90 80 70 60 50 40 30 20 10 0 −10 −20 −30
f1 (ppm)

NMR data of *N*-(2-bromophenyl)-*N*-methyl-4-(trifluoromethyl)benzamide

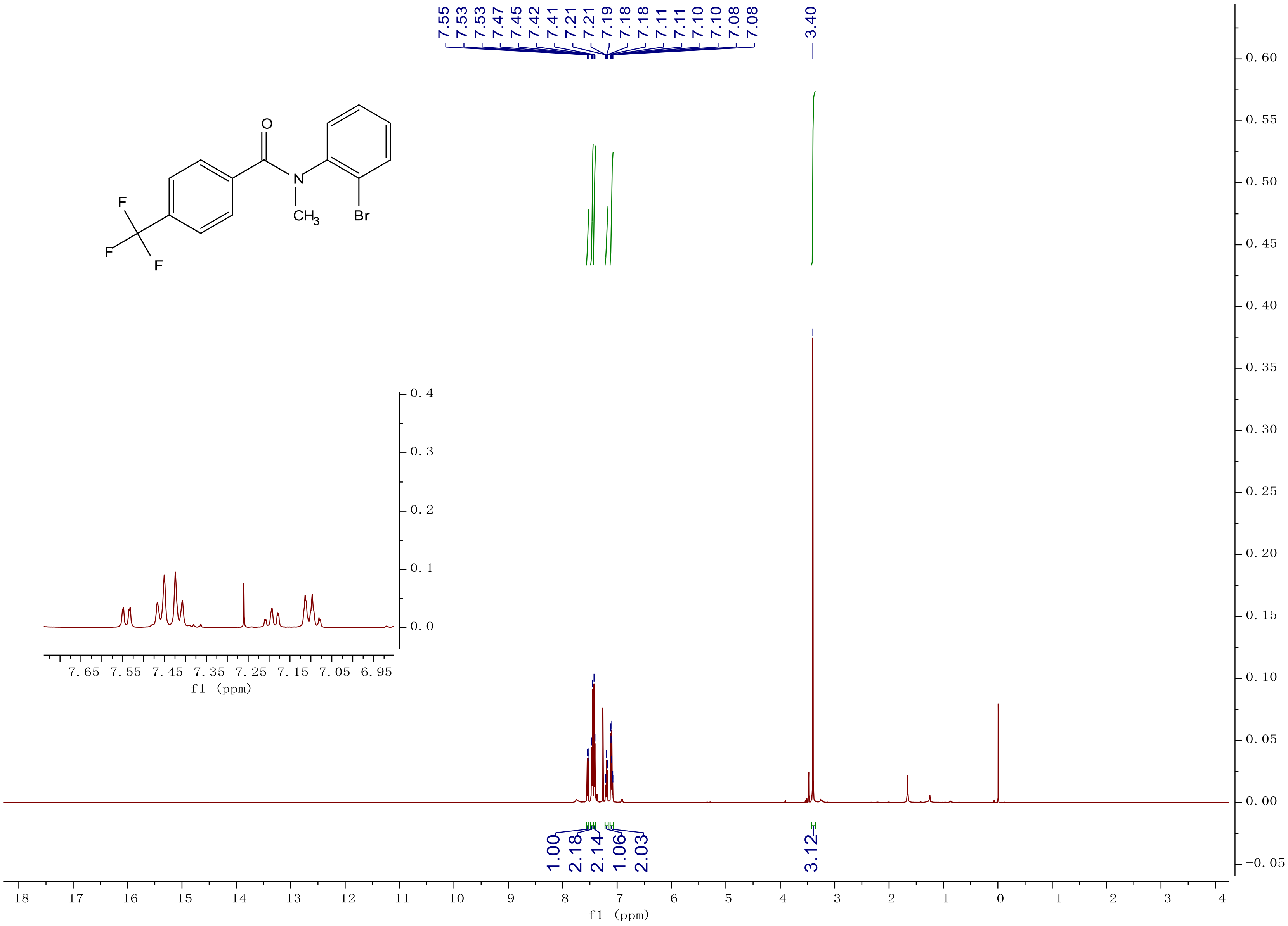

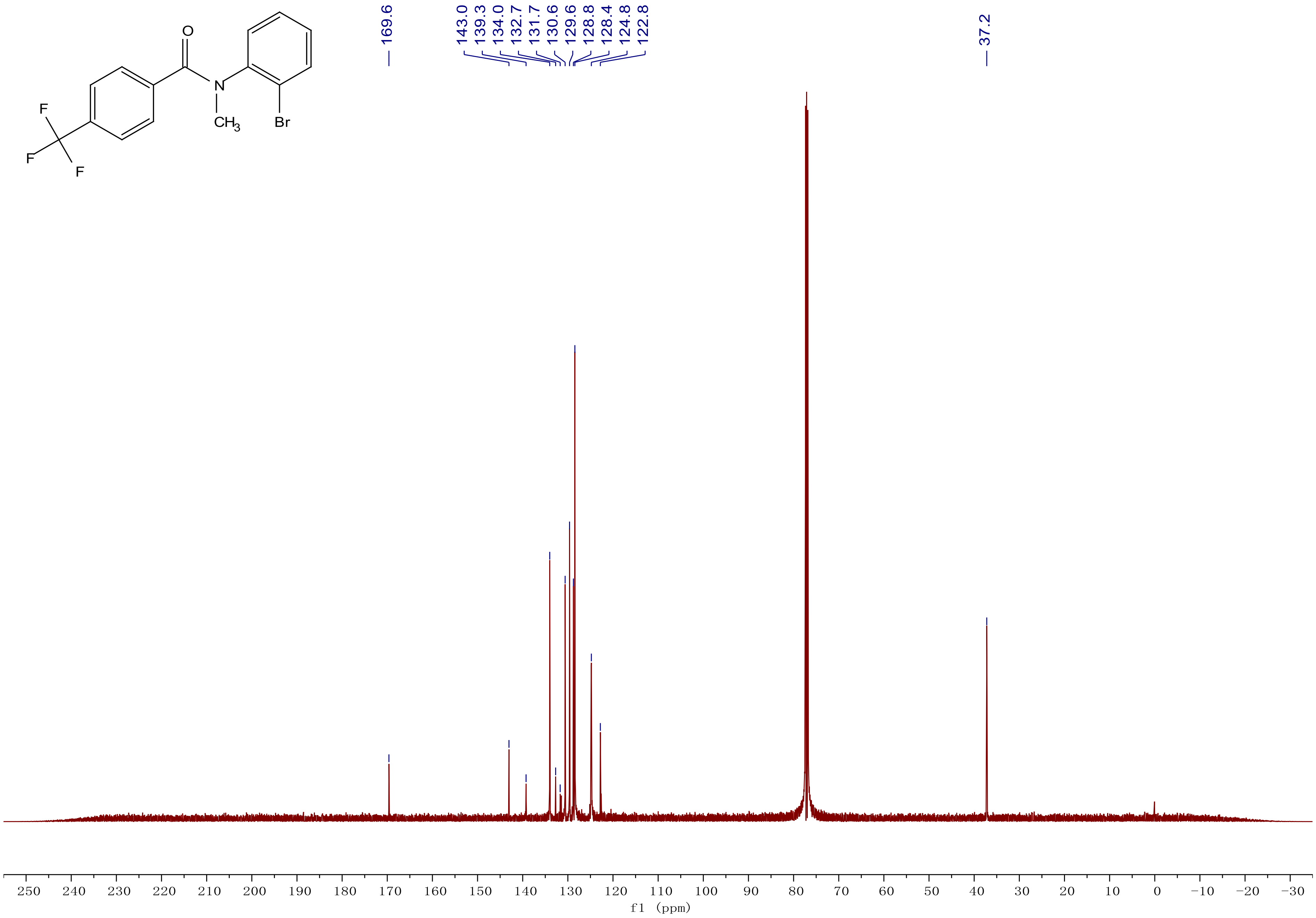

O
N
CH3
Br
F
F
F
169.6
143.0
139.3
134.0
132.7
131.7
130.6
129.6
128.8
128.4
124.8
122.8
37.2
250 240 230 220 210 200 190 180 170 160 150 140 130 120 110 100 90 80 70 60 50 40 30 20 10 0 −10 −20 −30
f1 (ppm)

NMR data of compound 4-bromo-*N*-(2-bromophenyl)-*N*-methylbenzamide

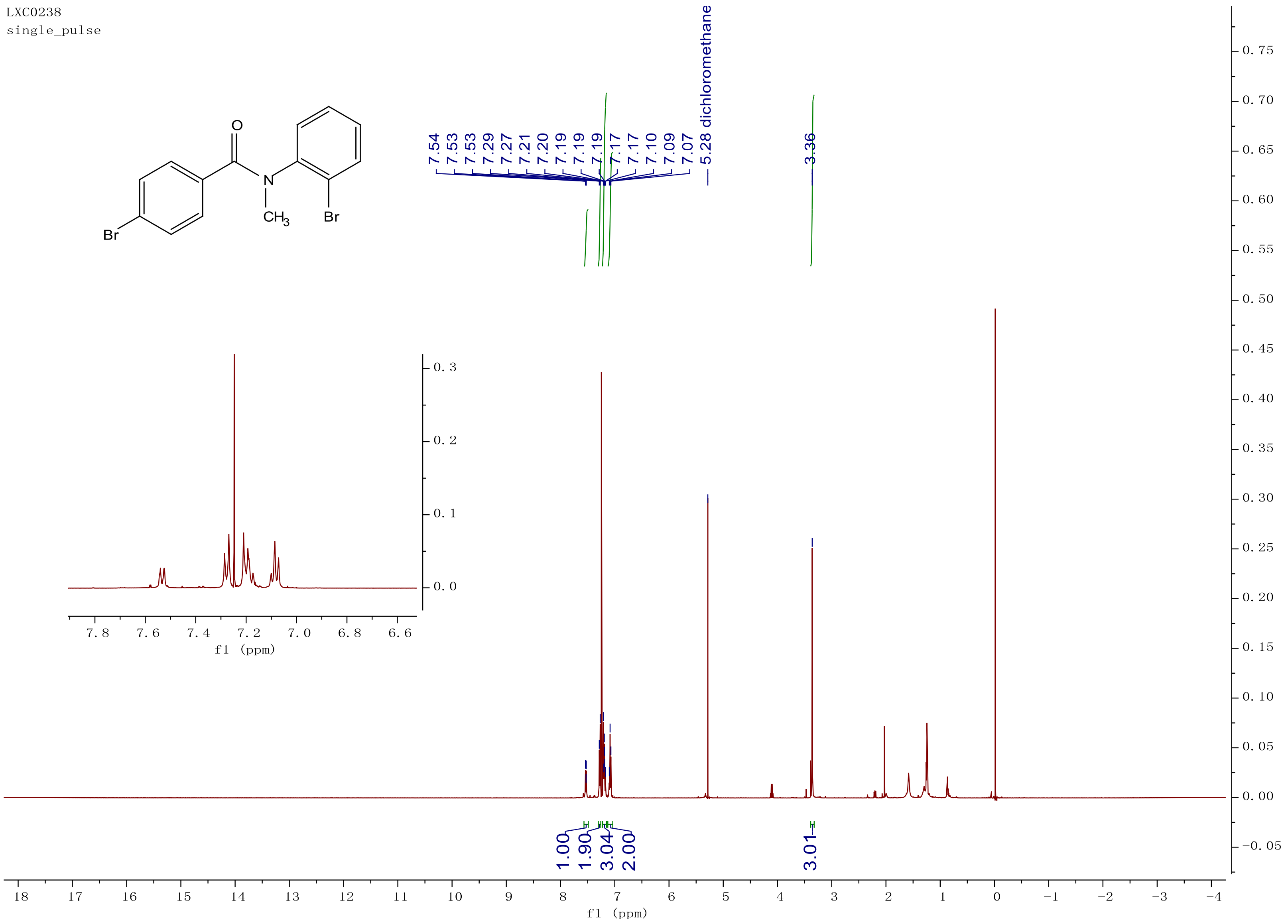

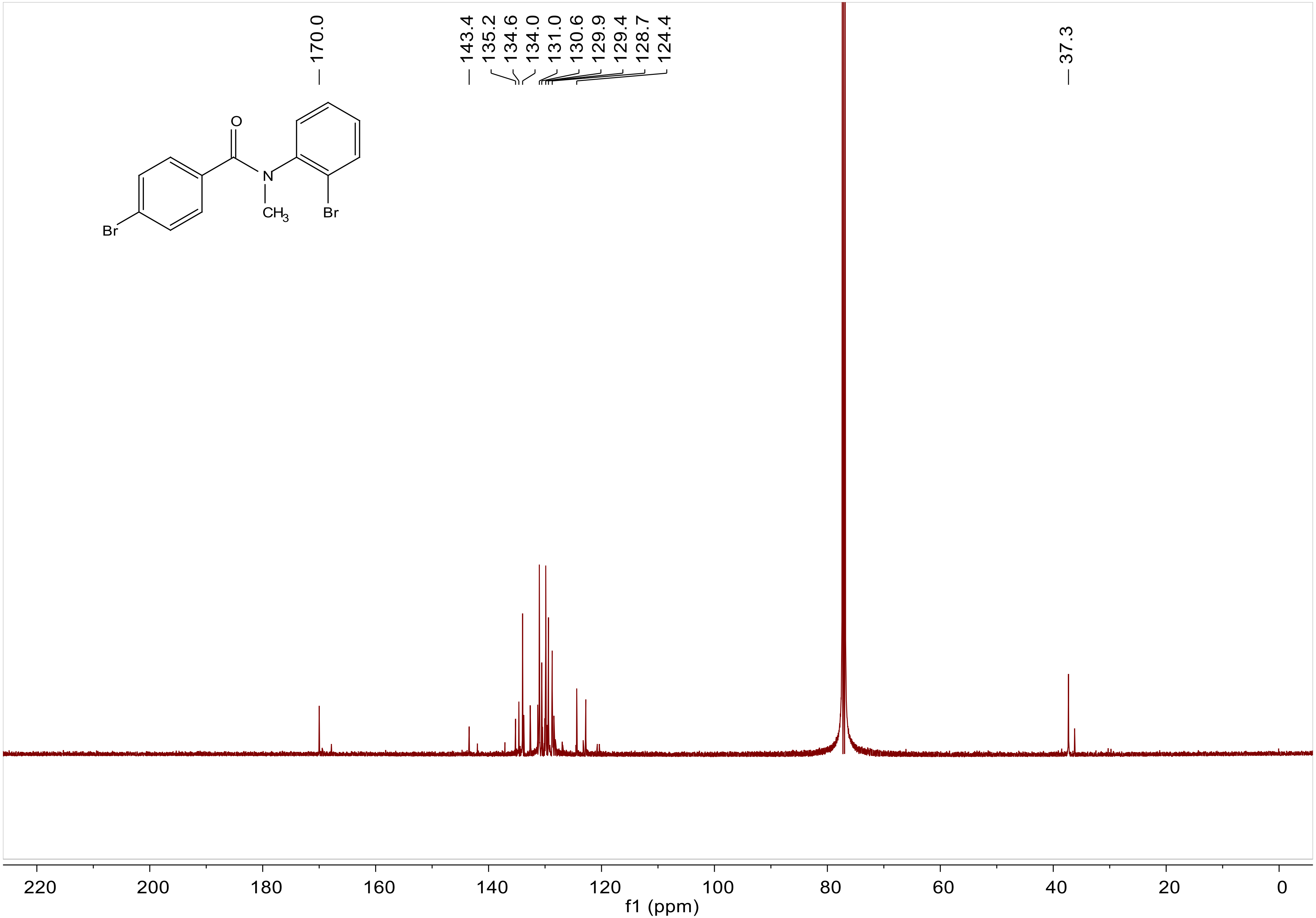
170.0
143.4
135.2
134.6
134.0
131.0
130.6
129.9
129.4
128.7
124.4
37.3
O
N
CH3
Br
Br
220
200
180
160
140
120
100
80
60
40
20
0
f1 (ppm)

NMR data of compound *N*-(2-bromophenyl)-4-chloro-*N*-methylbenzamide

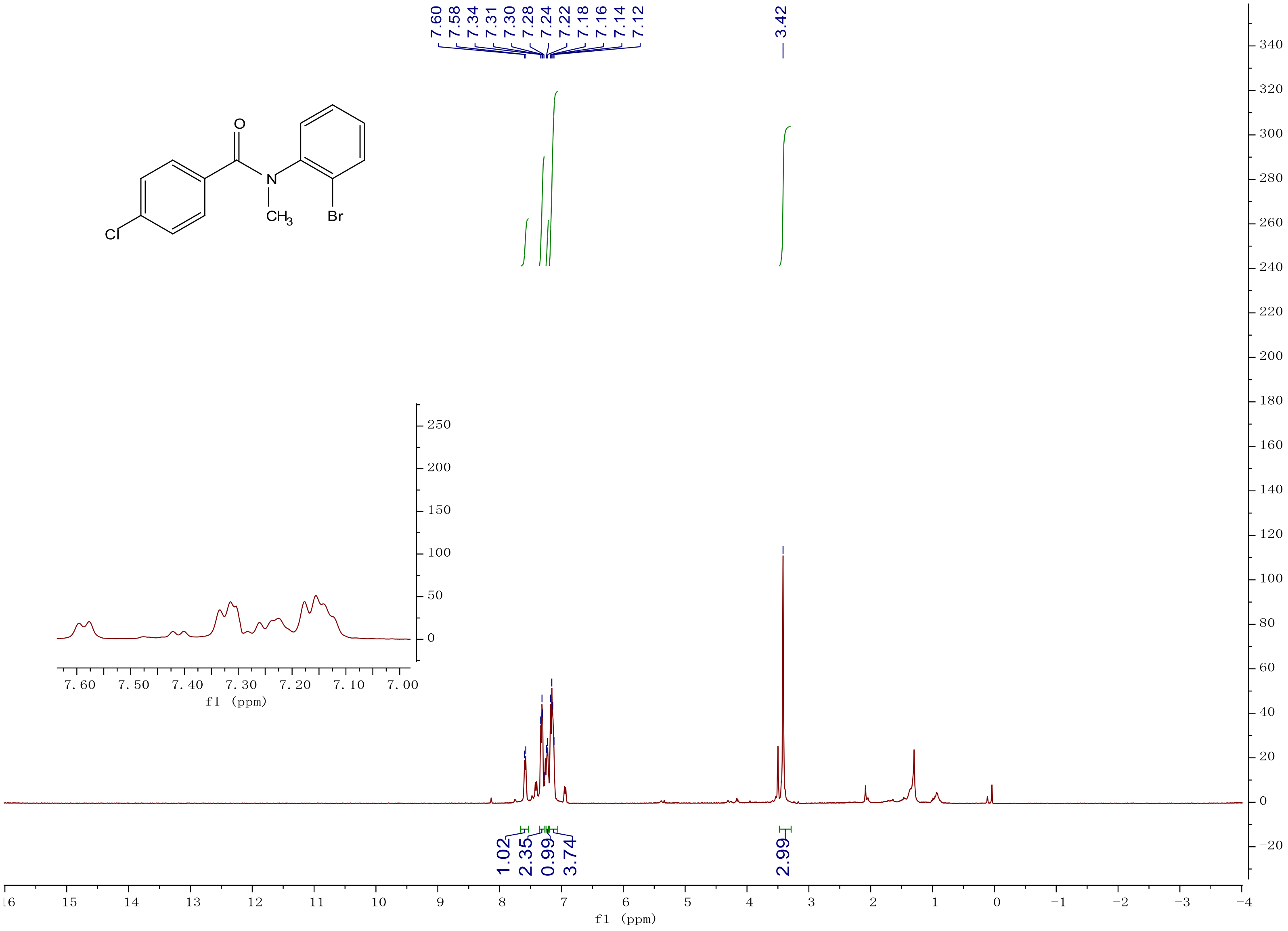

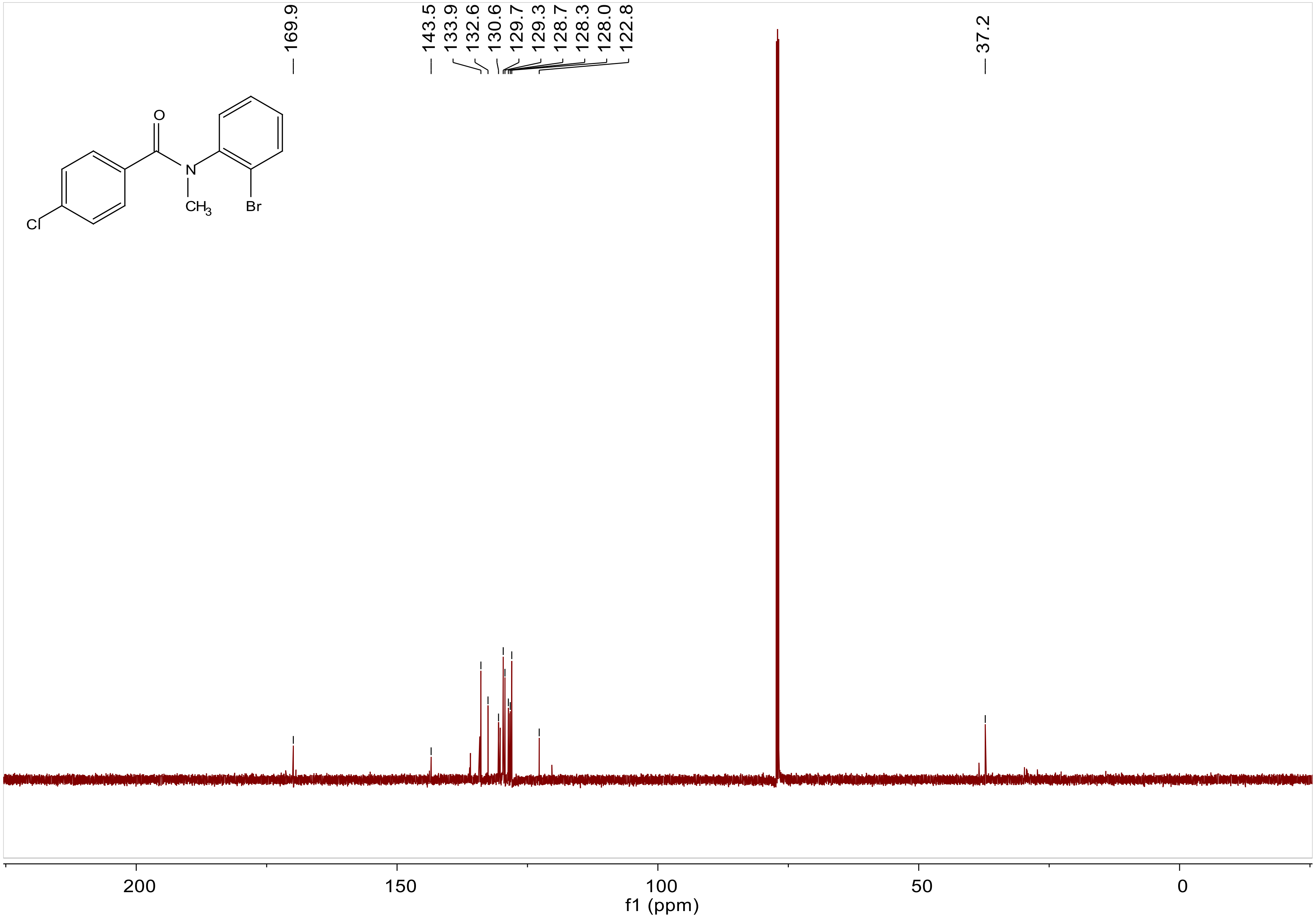

169.9
143.5
133.9
132.6
130.6
129.7
129.3
128.7
128.3
128.0
122.8
37.2
O
N
CH3
Br
Cl
200
150
100
50
0
f1 (ppm)

NMR data of compound 3-bromo-*N*-(2-bromophenyl)-*N*-methylbenzamide

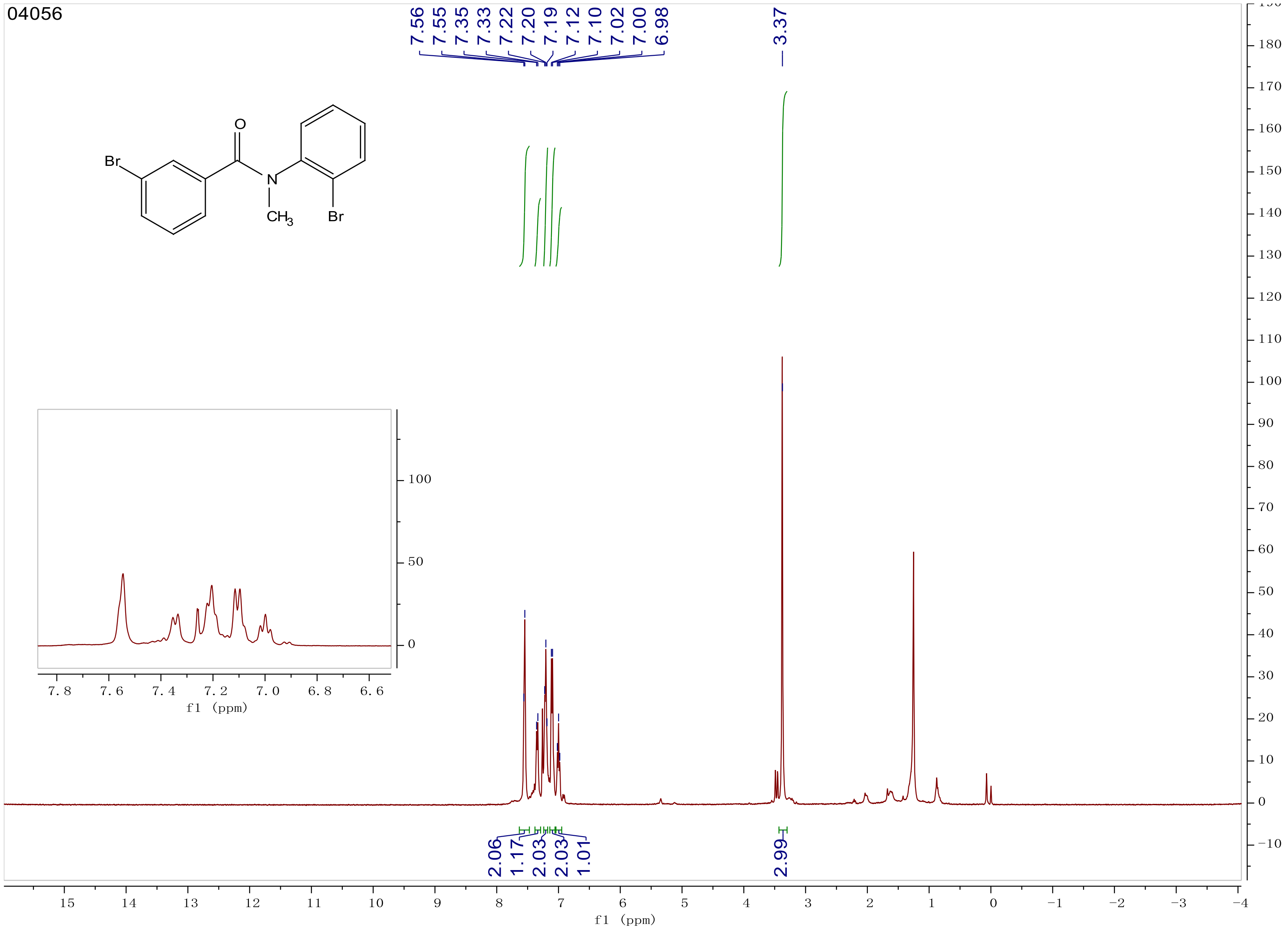

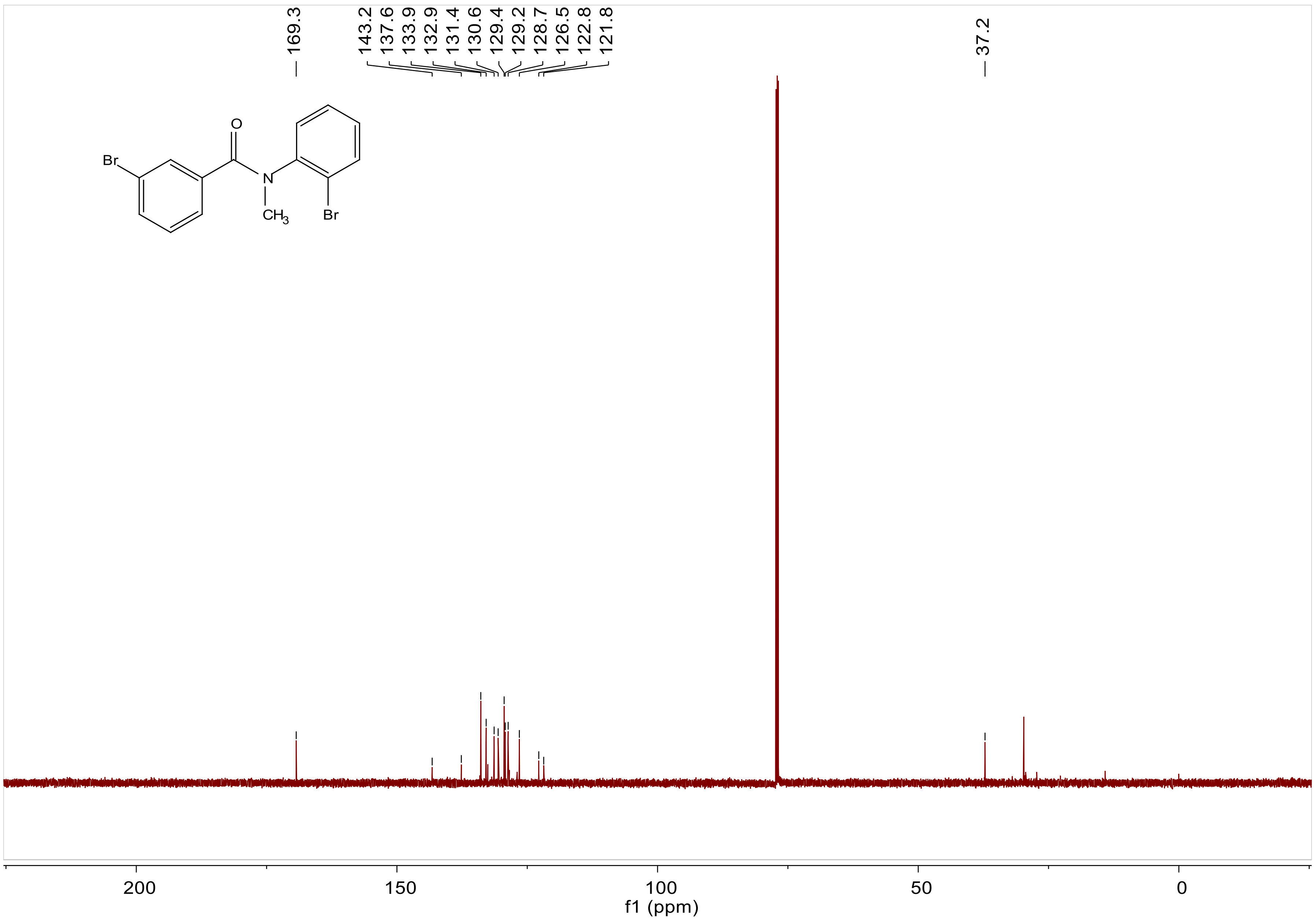
169.3
143.2
137.6
133.9
132.9
131.4
130.6
129.4
129.2
128.7
126.5
122.8
121.8
37.2
O
Br
N
CH3
Br
200
150
100
50
0
f1 (ppm)

NMR data of compound *N*-(2-bromophenyl)-3-fluoro-*N*-methylbenzamide

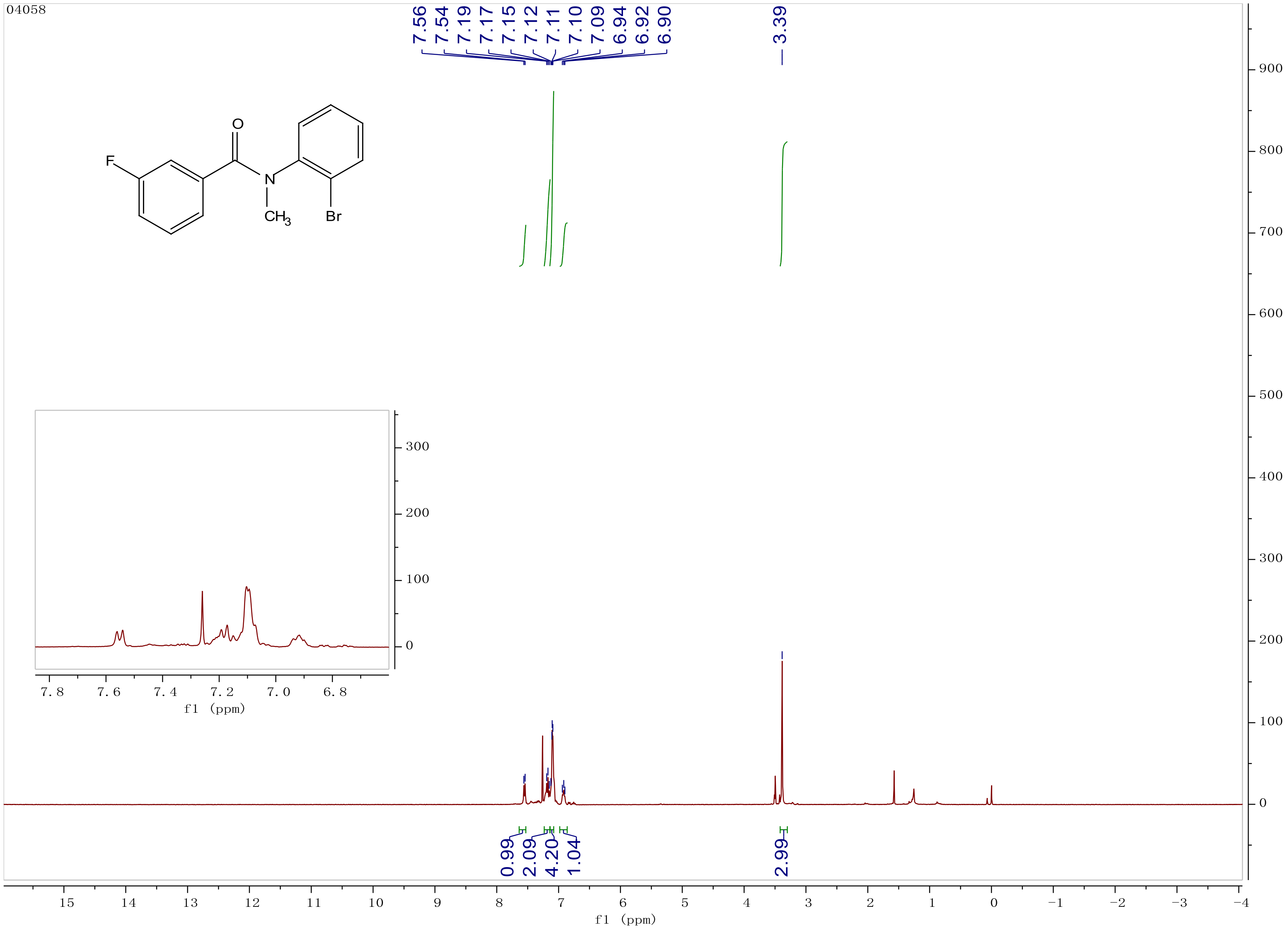

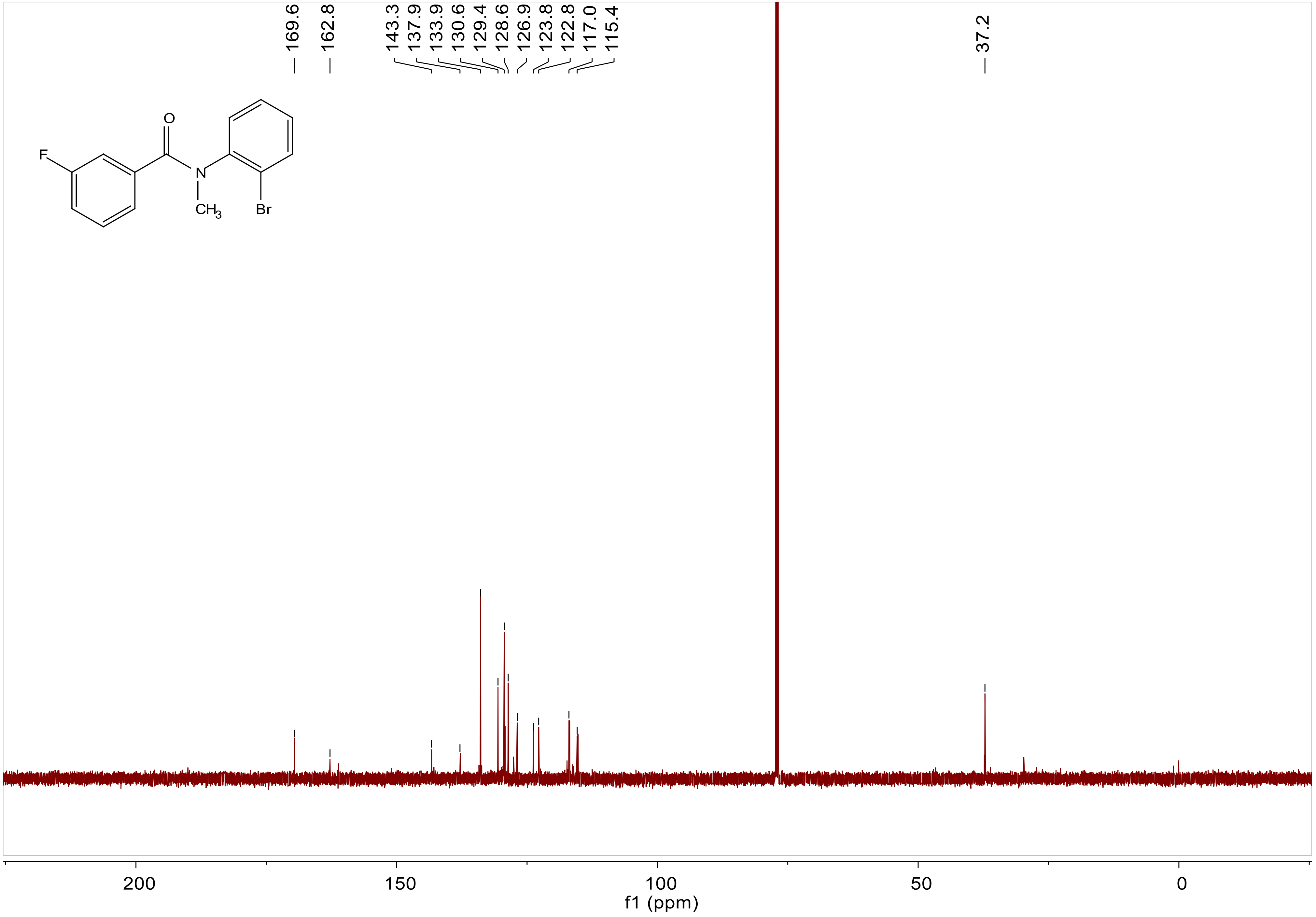

169.6
162.8
143.3
137.9
133.9
130.6
129.4
128.6
126.9
123.8
122.8
117.0
115.4
37.2
F
O
N
CH3
Br
200
150
100
50
0
f1 (ppm)

NMR data of compound *N*-(2-bromophenyl)-2-fluoro-*N*-methylbenzamide

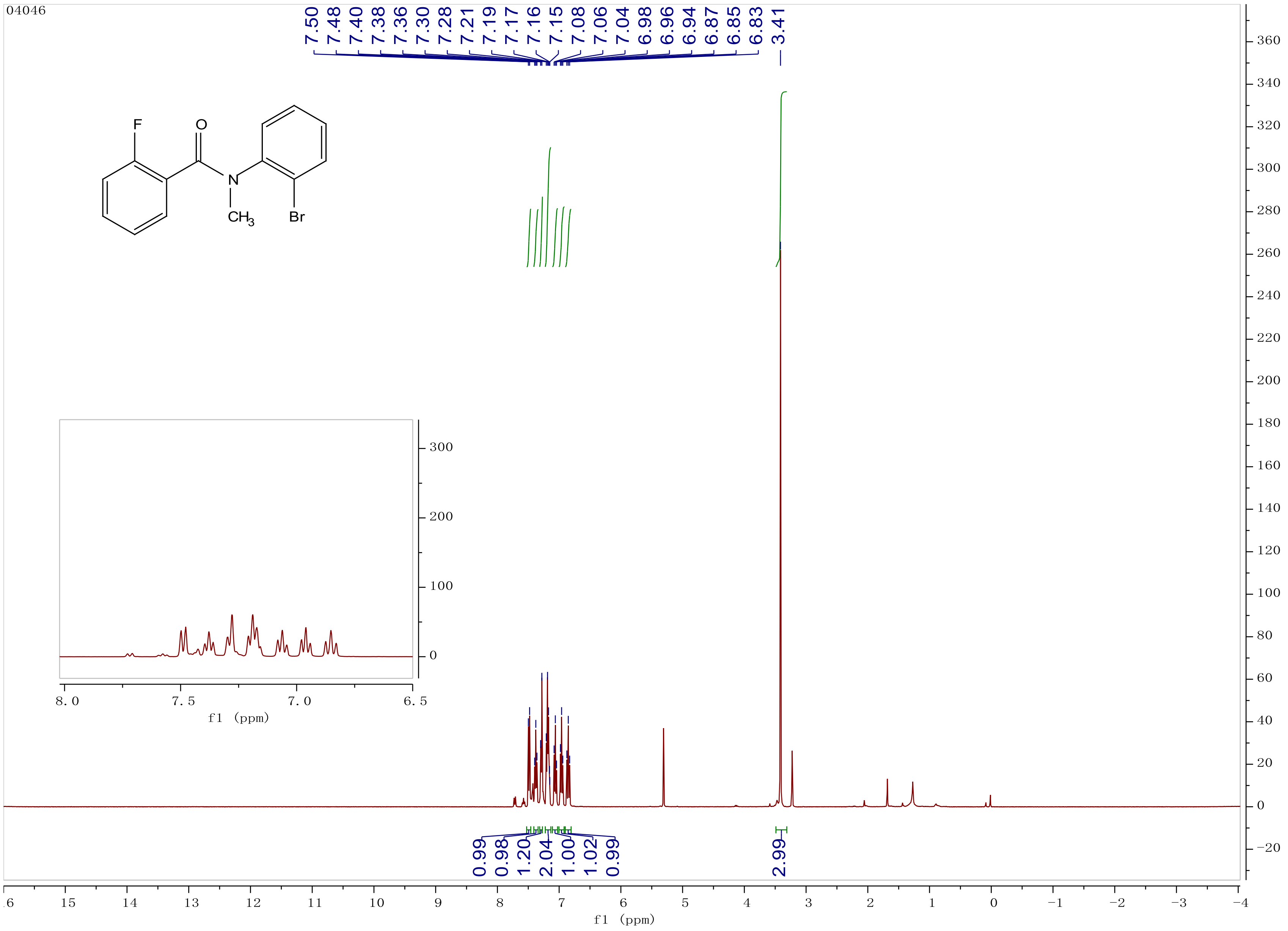

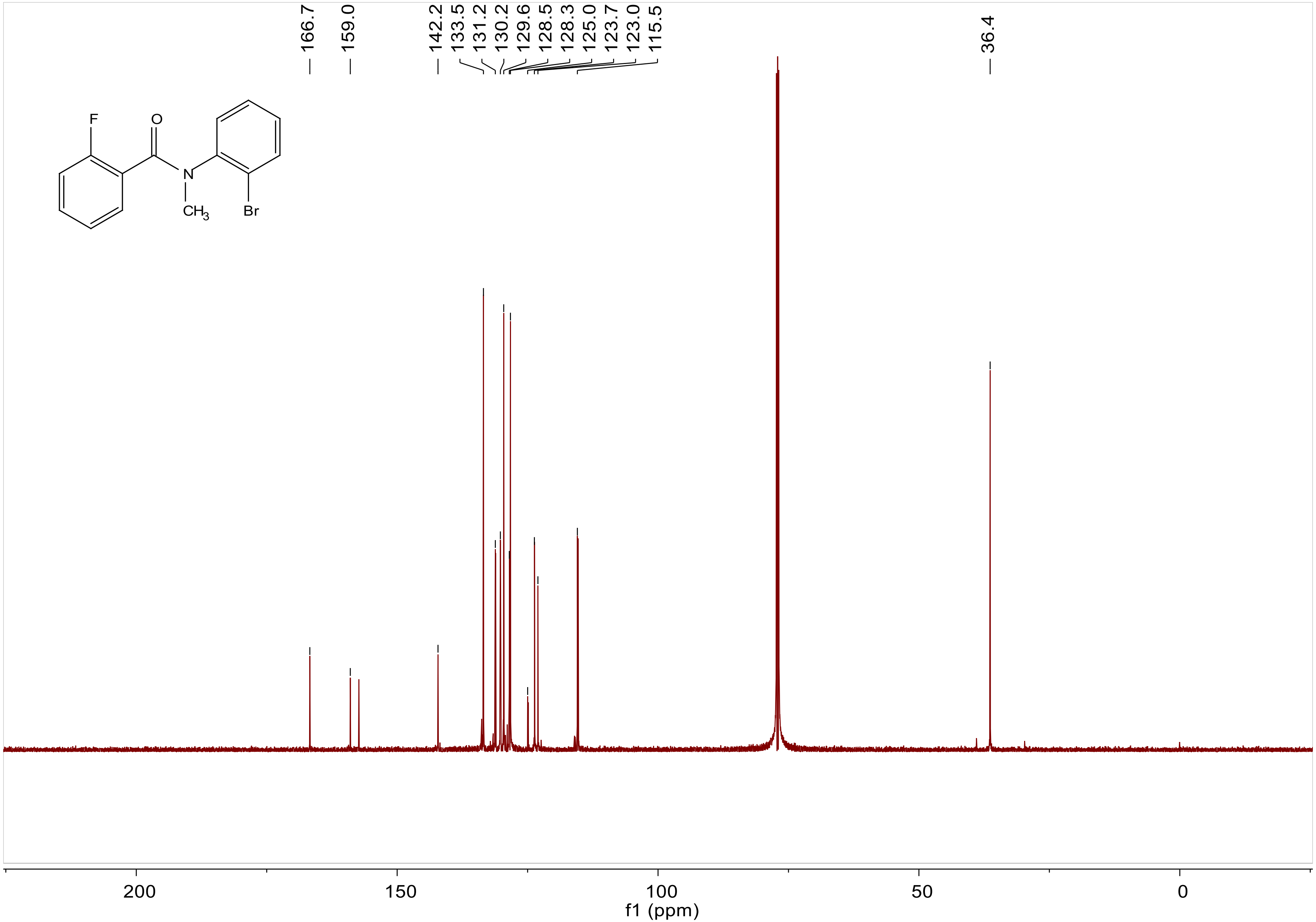
166.7
159.0
142.2
133.5
131.2
130.2
129.6
128.5
128.3
125.0
123.7
123.0
115.5
36.4
F
O
N
CH3
Br
200
150
100
50
0
f1 (ppm)

NMR data of compound *N*-(2-bromophenyl)-4-iodo-*N*-methylbenzamide

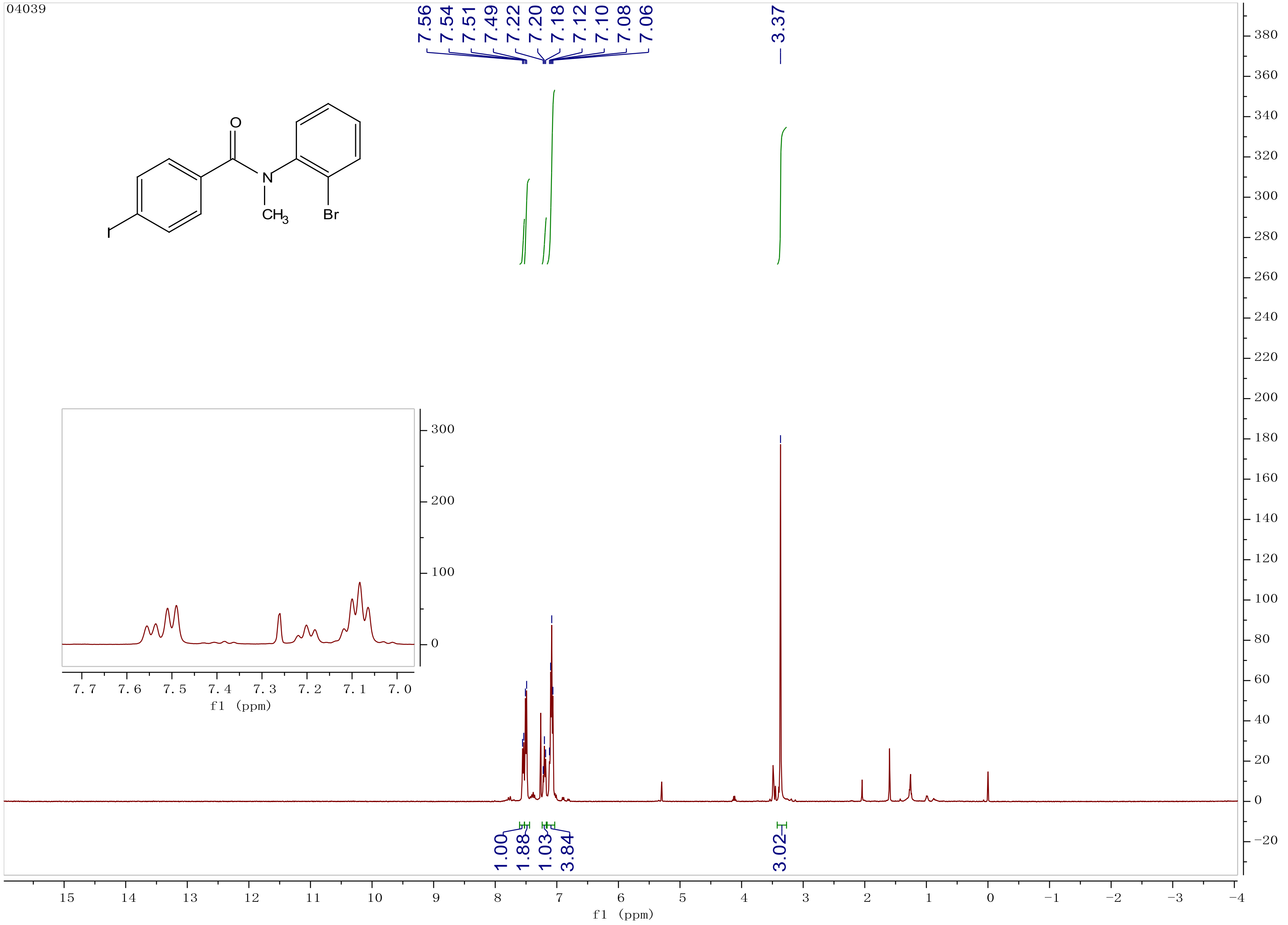

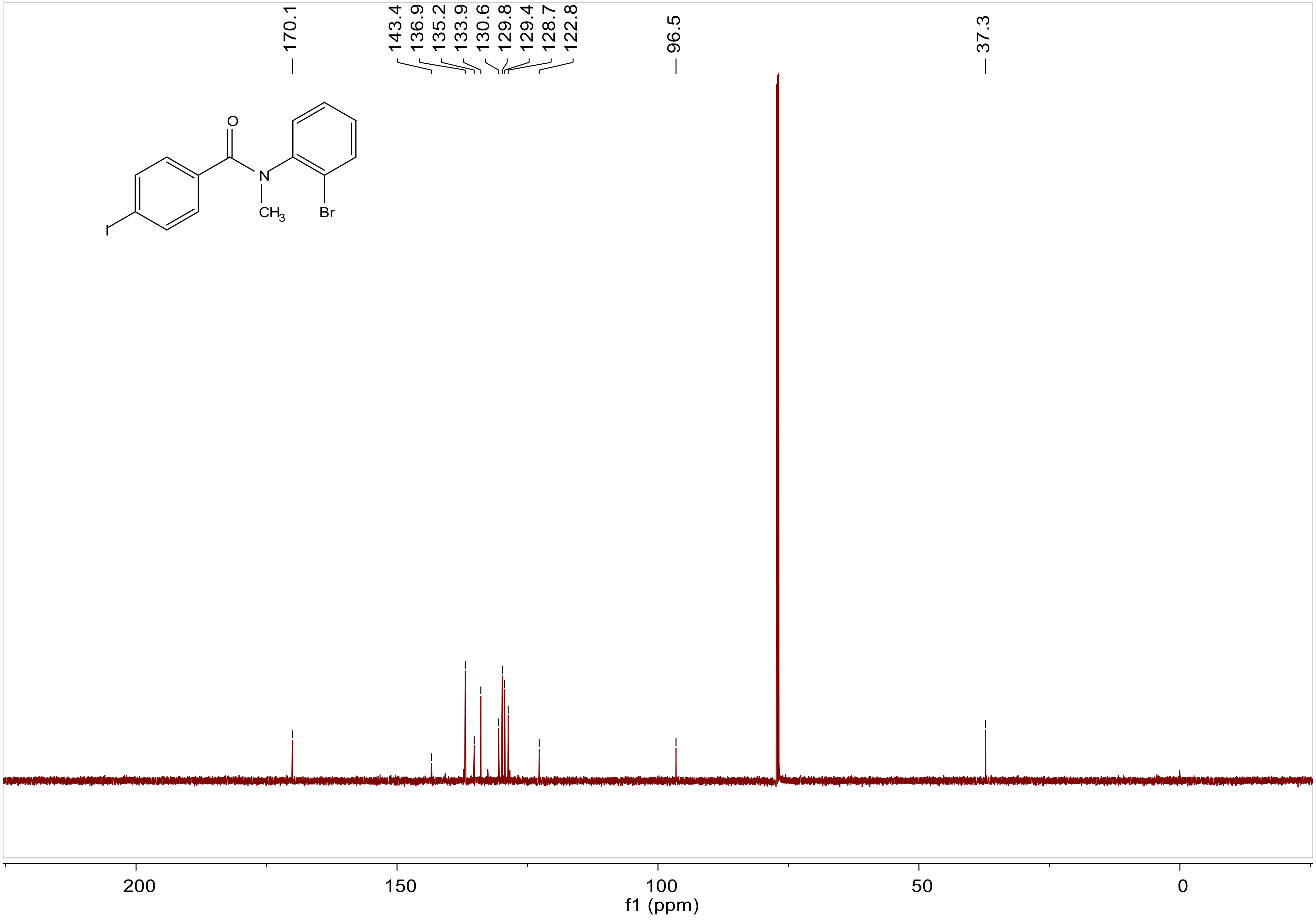
170.1
143.4
136.9
135.2
133.9
130.6
129.8
129.4
128.7
122.8
96.5
37.3
O
N
CH3
Br
I
200
150
100
50
0
f1 (ppm)

NMR data of compound *N*-(2-bromophenyl)-2-chloro-*N*-methylacetamide

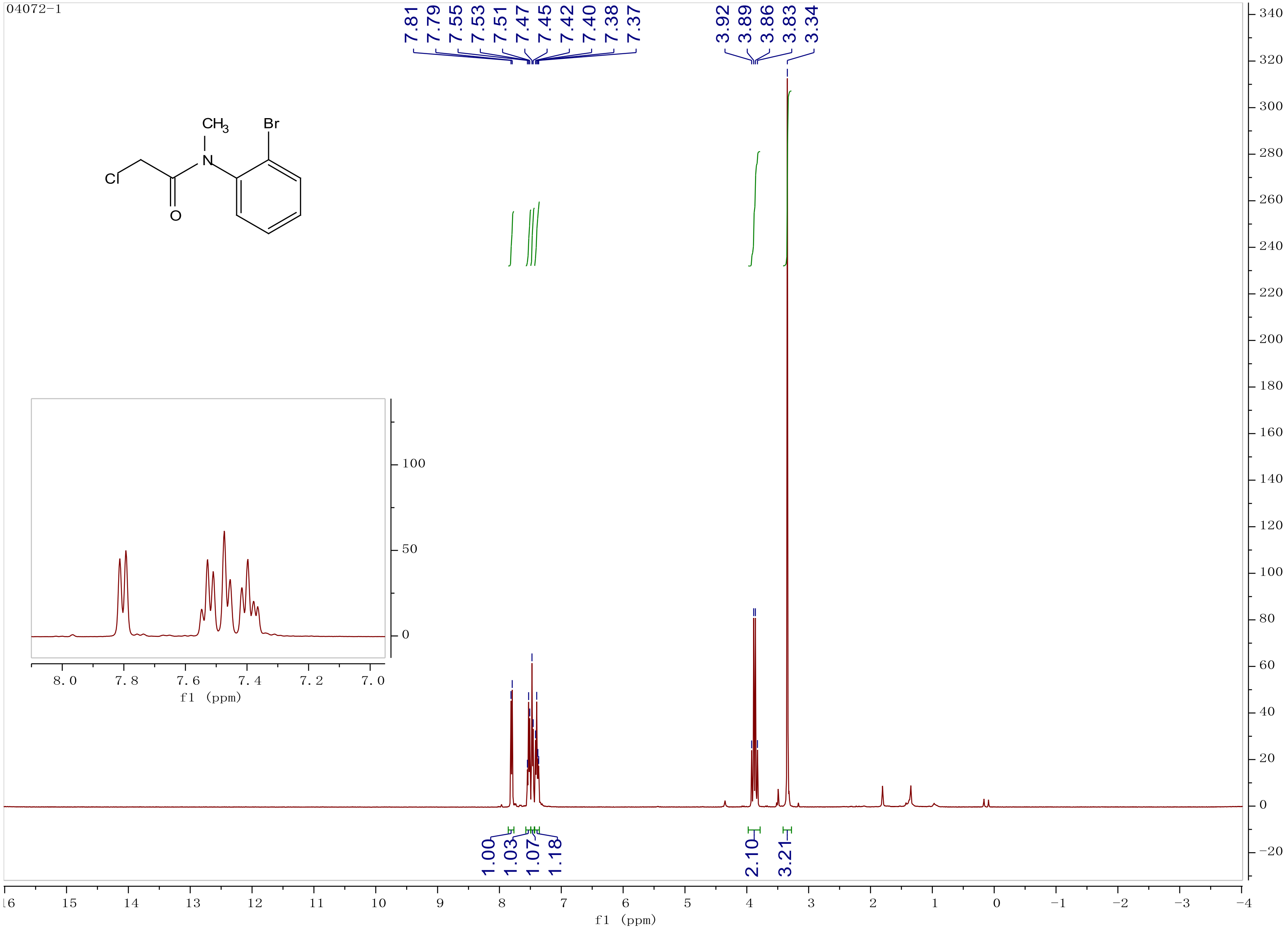

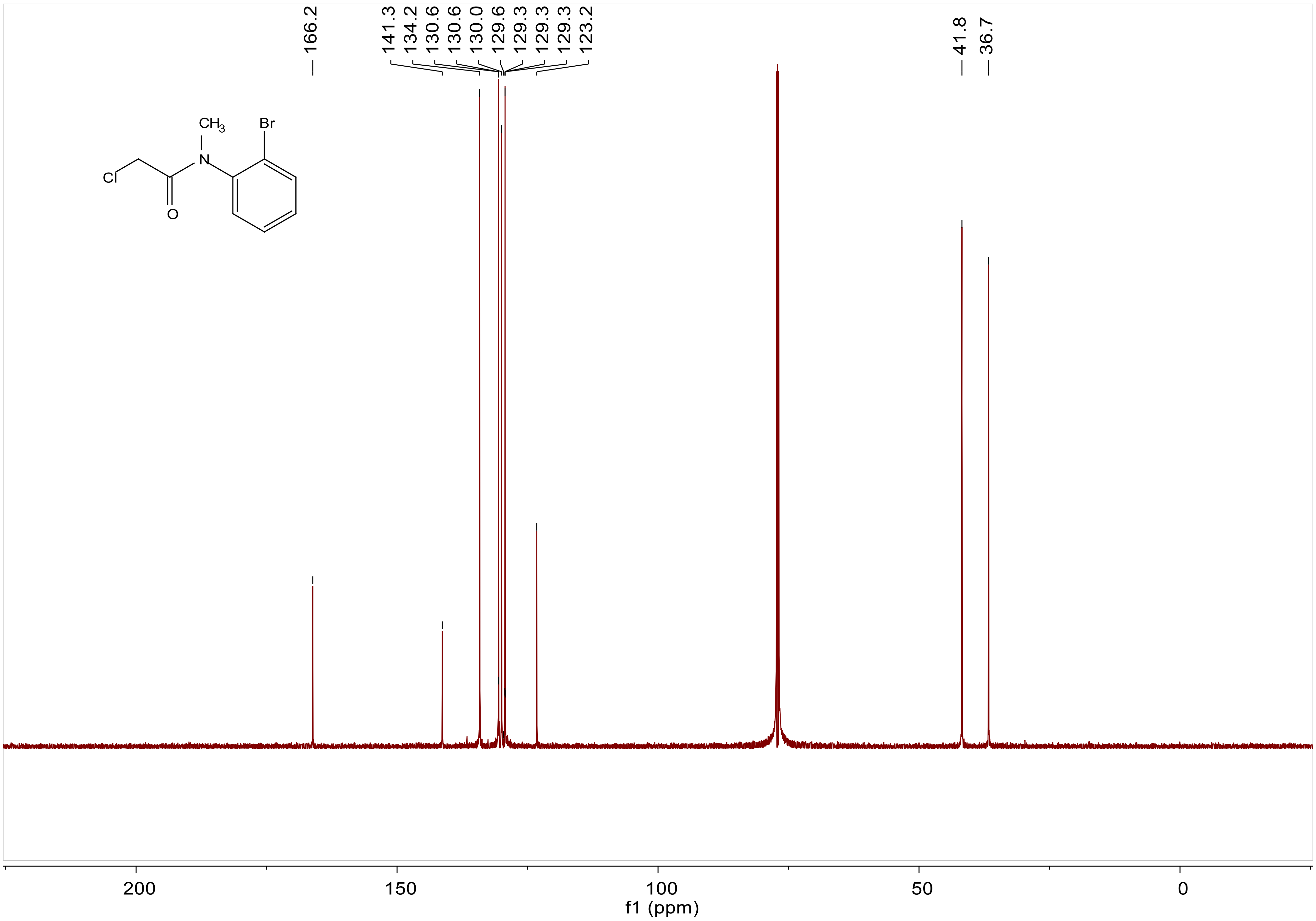
166.2
141.3
134.2
130.6
130.6
130.0
129.6
129.3
129.3
129.3
123.2
41.8
36.7
CH3
Br
Cl
N
O
200
150
100
50
0
f1 (ppm)

NMR data of compound *N*-(2-bromophenyl)-*N*-methyl-4-nitrobenzamide

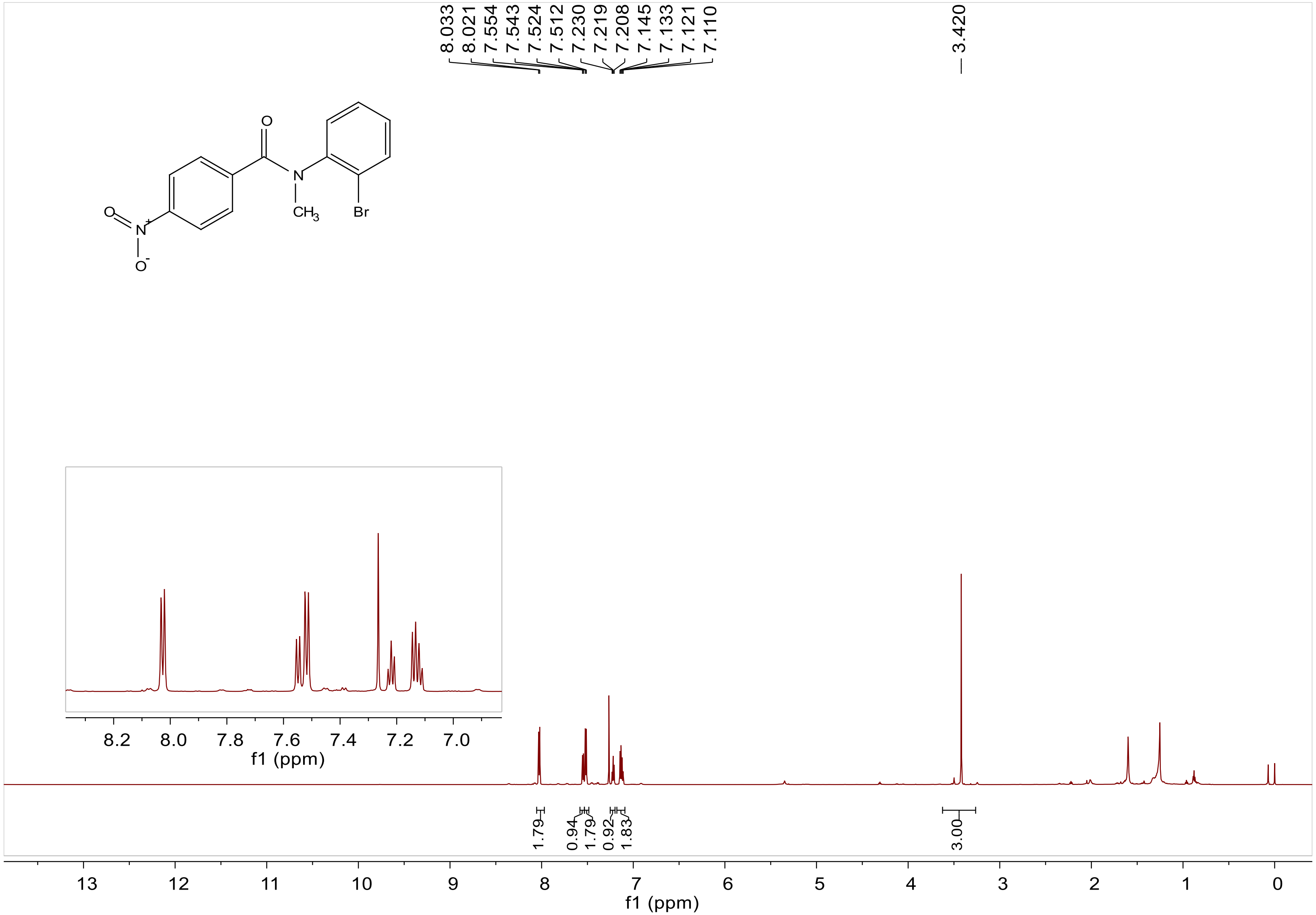

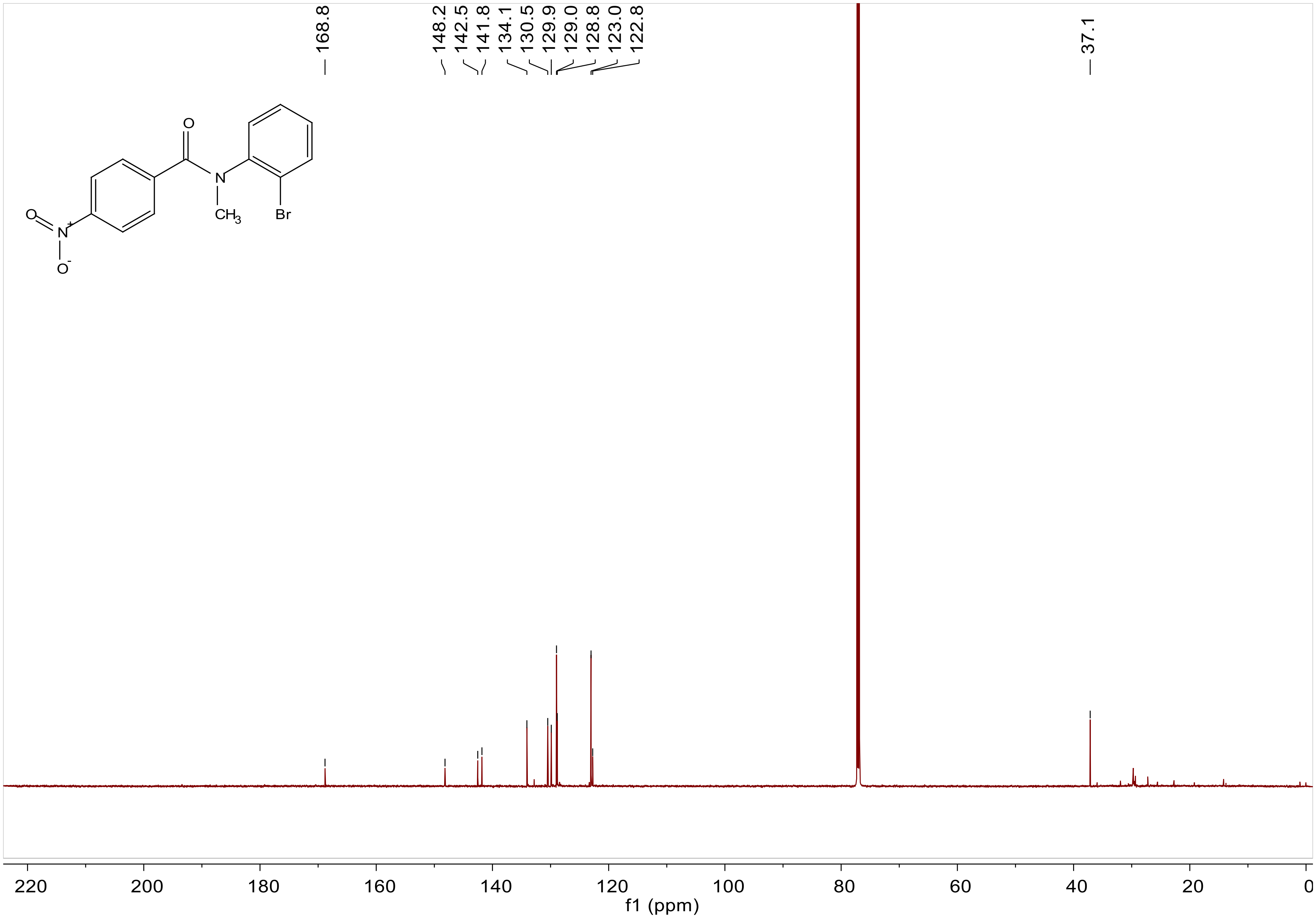
168.8
148.2
142.5
141.8
134.1
130.5
129.9
129.0
128.8
123.0
122.8
37.1
O
N
CH3
Br
O
N+
O-
220
200
180
160
140
120
100
80
60
40
20
0
f1 (ppm)

NMR data of compound *N*-(4-bromophenyl)-*N*-methylbenzamide

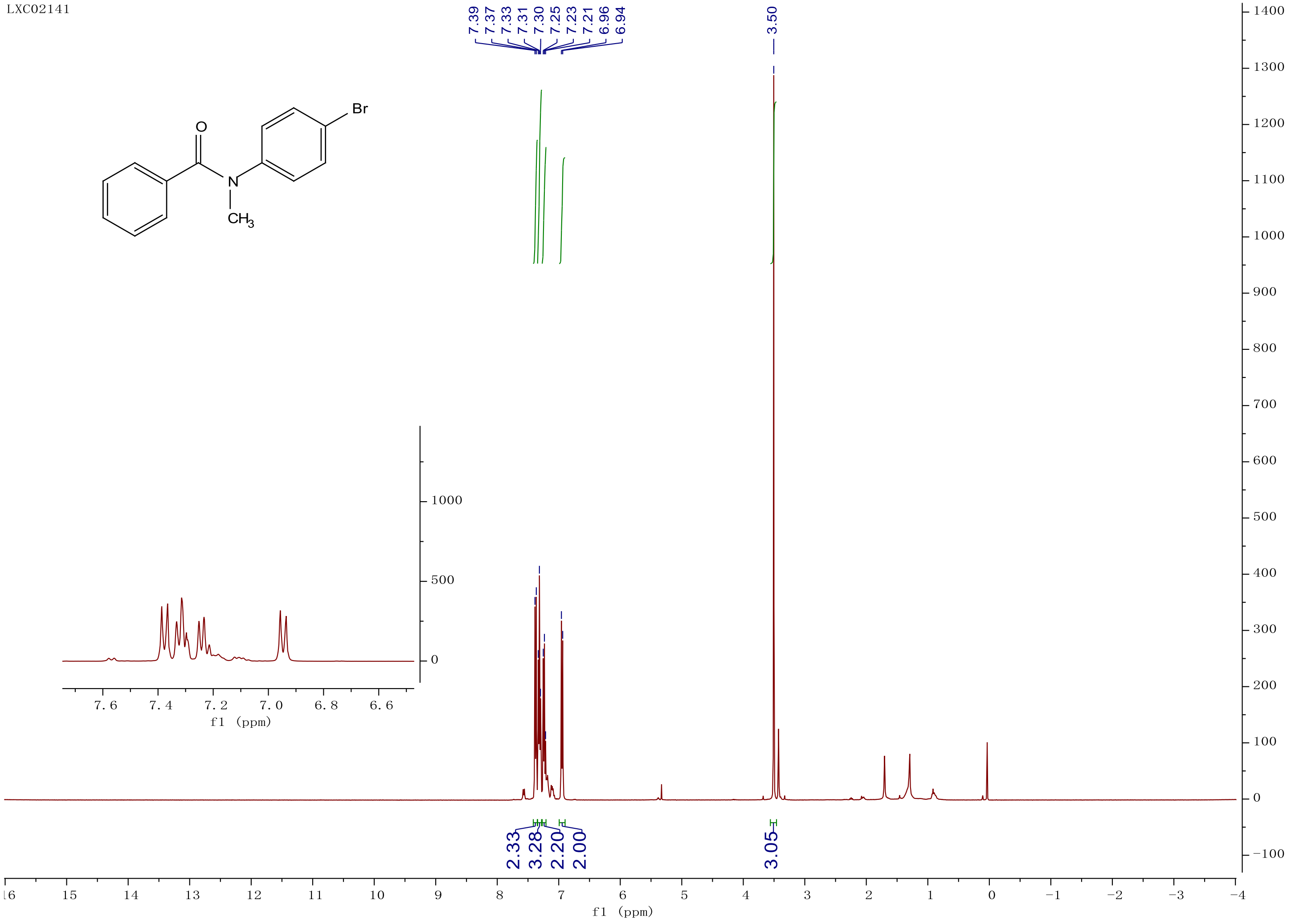

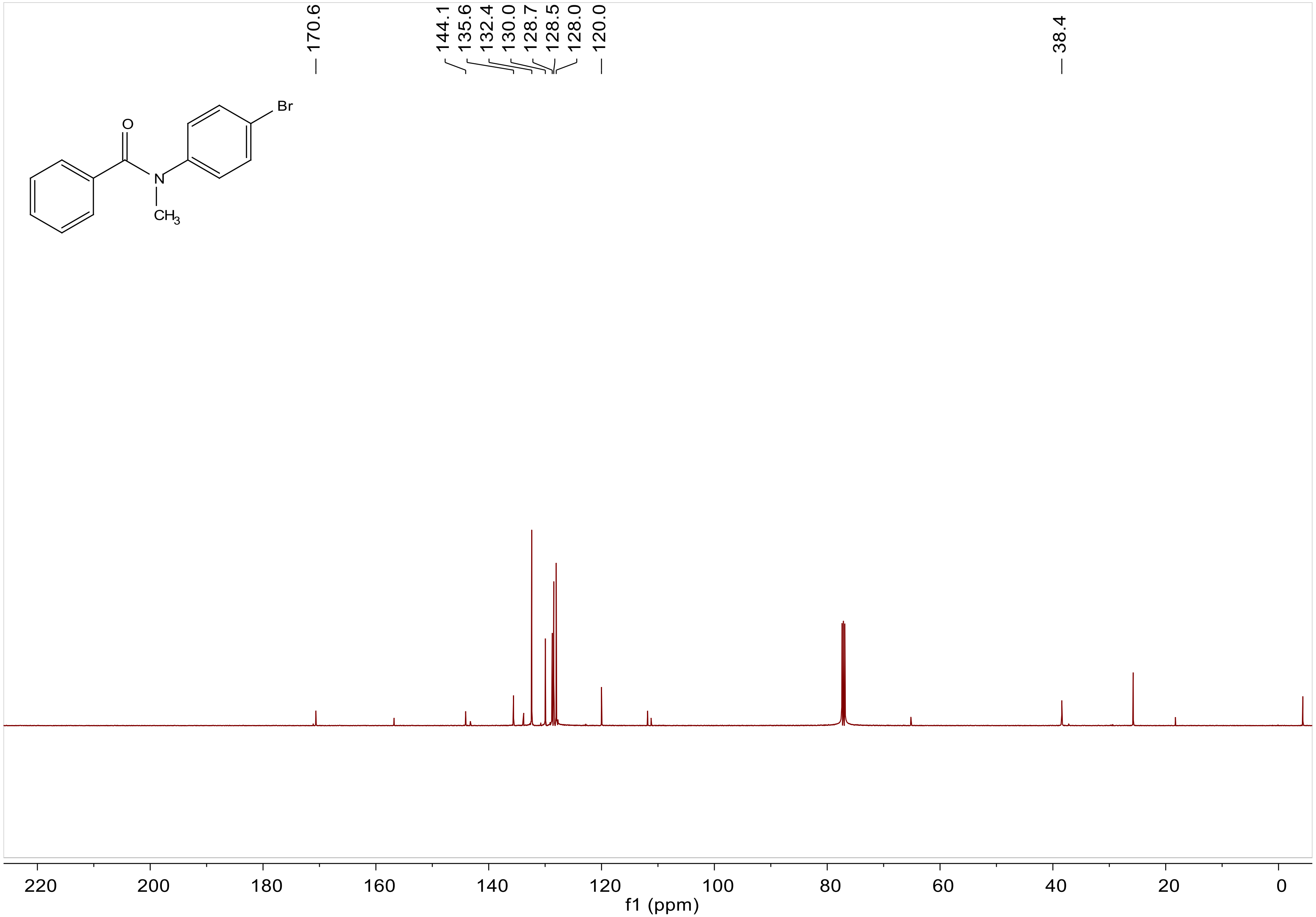
170.6
144.1
135.6
132.4
130.0
128.7
128.5
128.0
120.0
38.4
O
N
CH3
Br
220
200
180
160
140
120
100
80
60
40
20
0
f1 (ppm)

NMR data of compound *N*-(4-bromophenyl)-4-fluoro-*N*-methylbenzamide

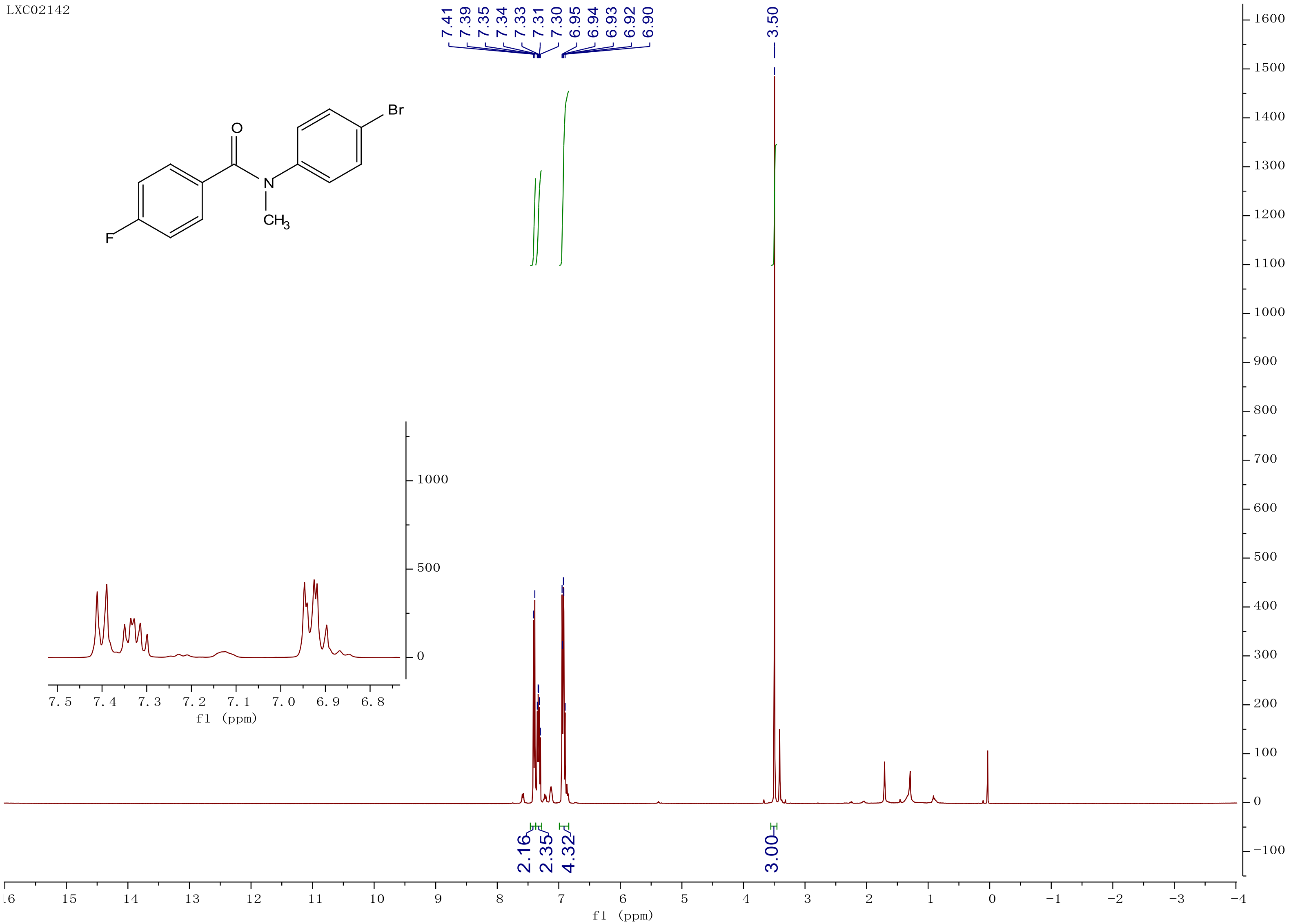

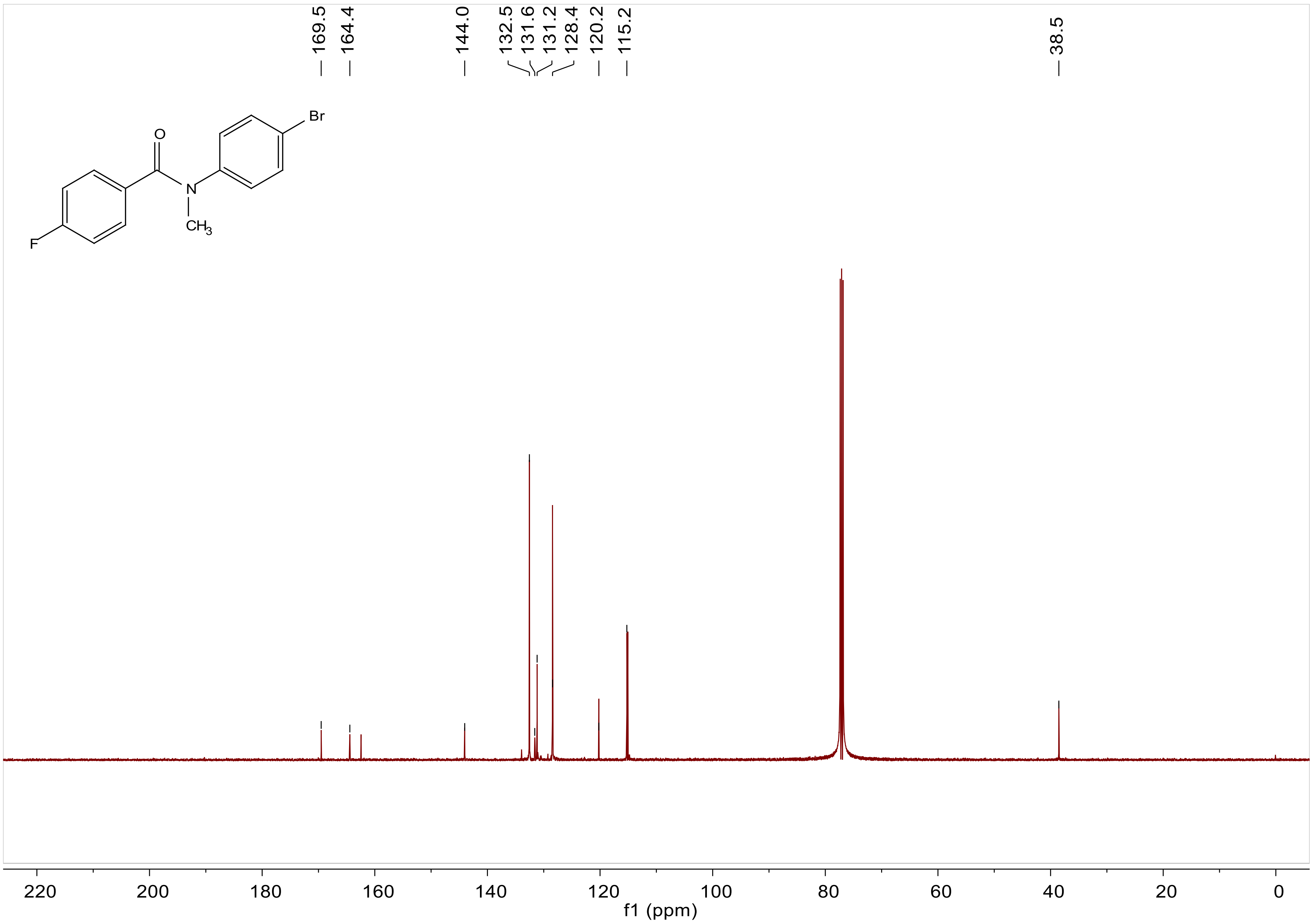

169.5
164.4
144.0
132.5
131.6
131.2
128.4
120.2
115.2
38.5
O
N
CH3
F
Br
220
200
180
160
140
120
100
80
60
40
20
0
f1 (ppm)

NMR data of *N*-(4-bromophenyl)-*N*-methyl-4-(trifluoromethyl)benzamide

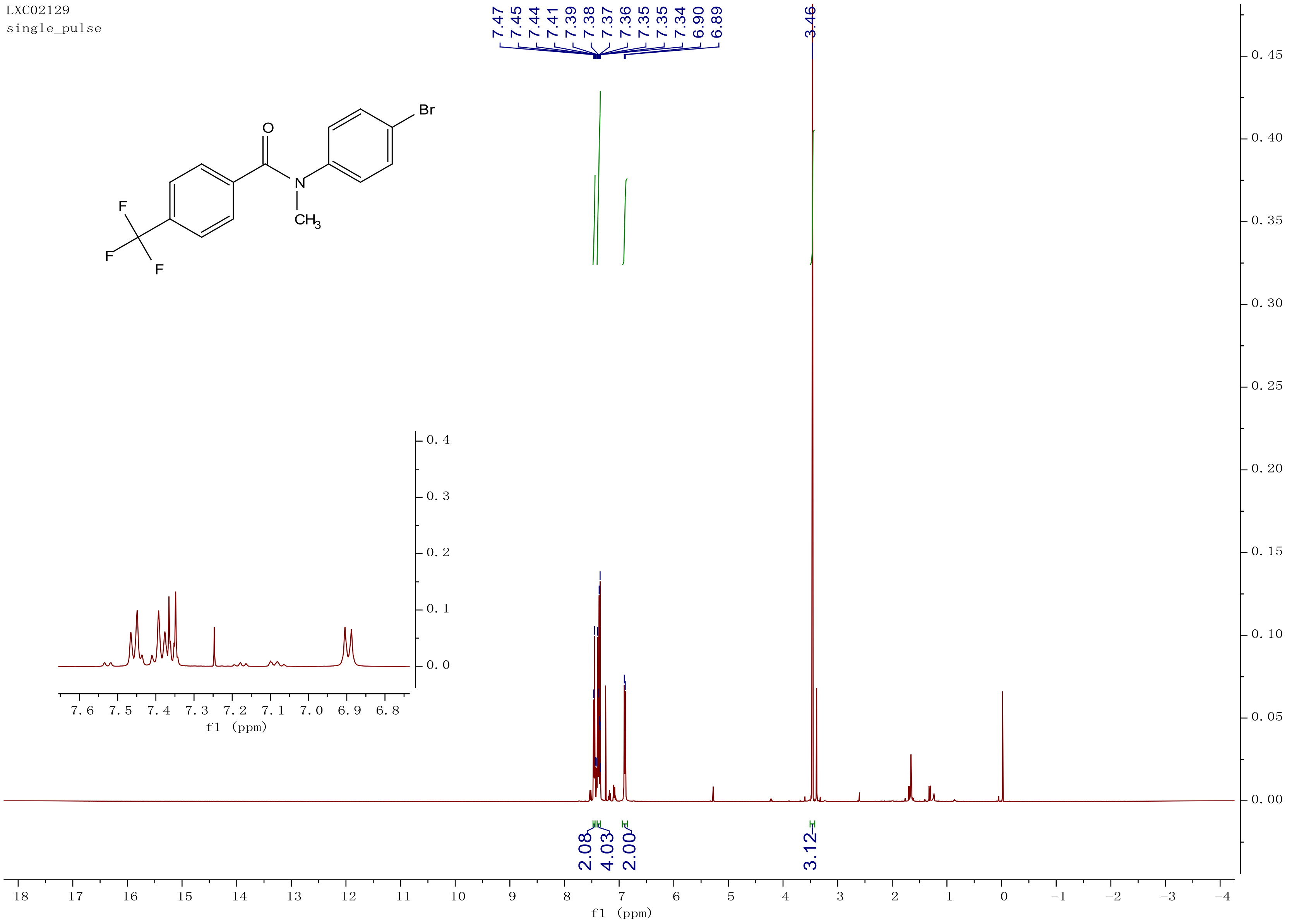

LXC02129
single pulse decoupled gated NOE

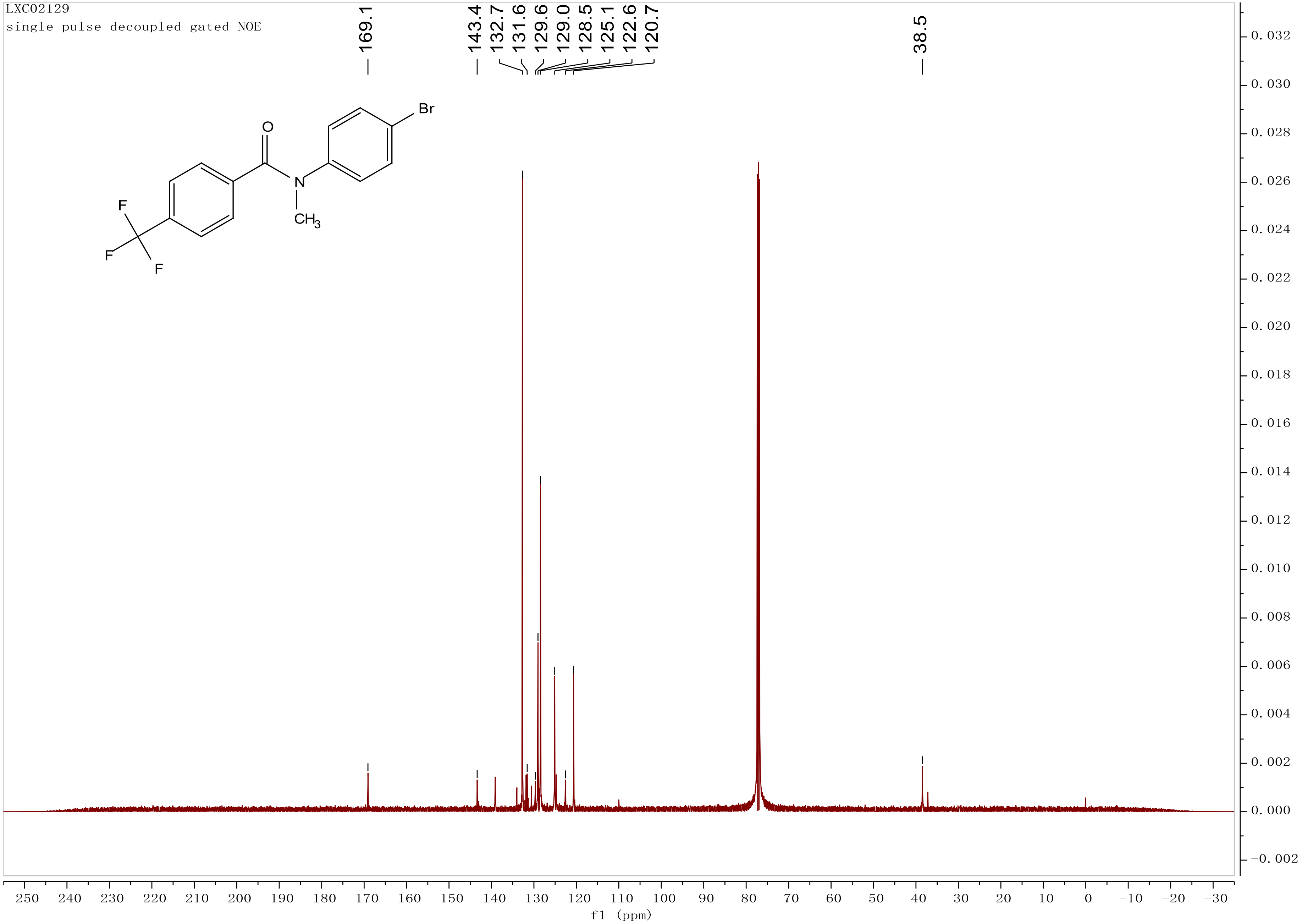

NMR data of compound 4-bromo-*N*-(4-bromophenyl)-*N*-methylbenzamide

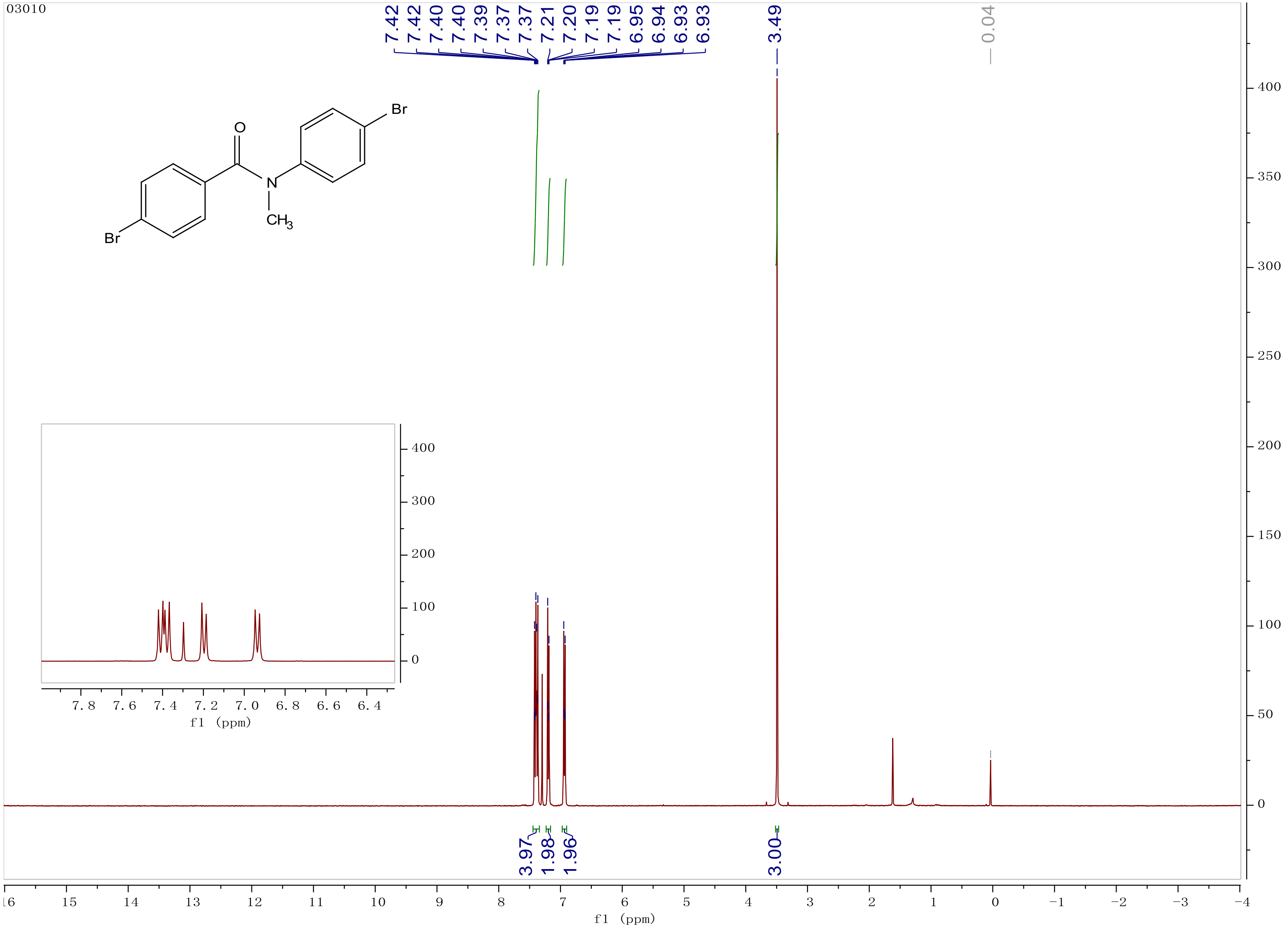

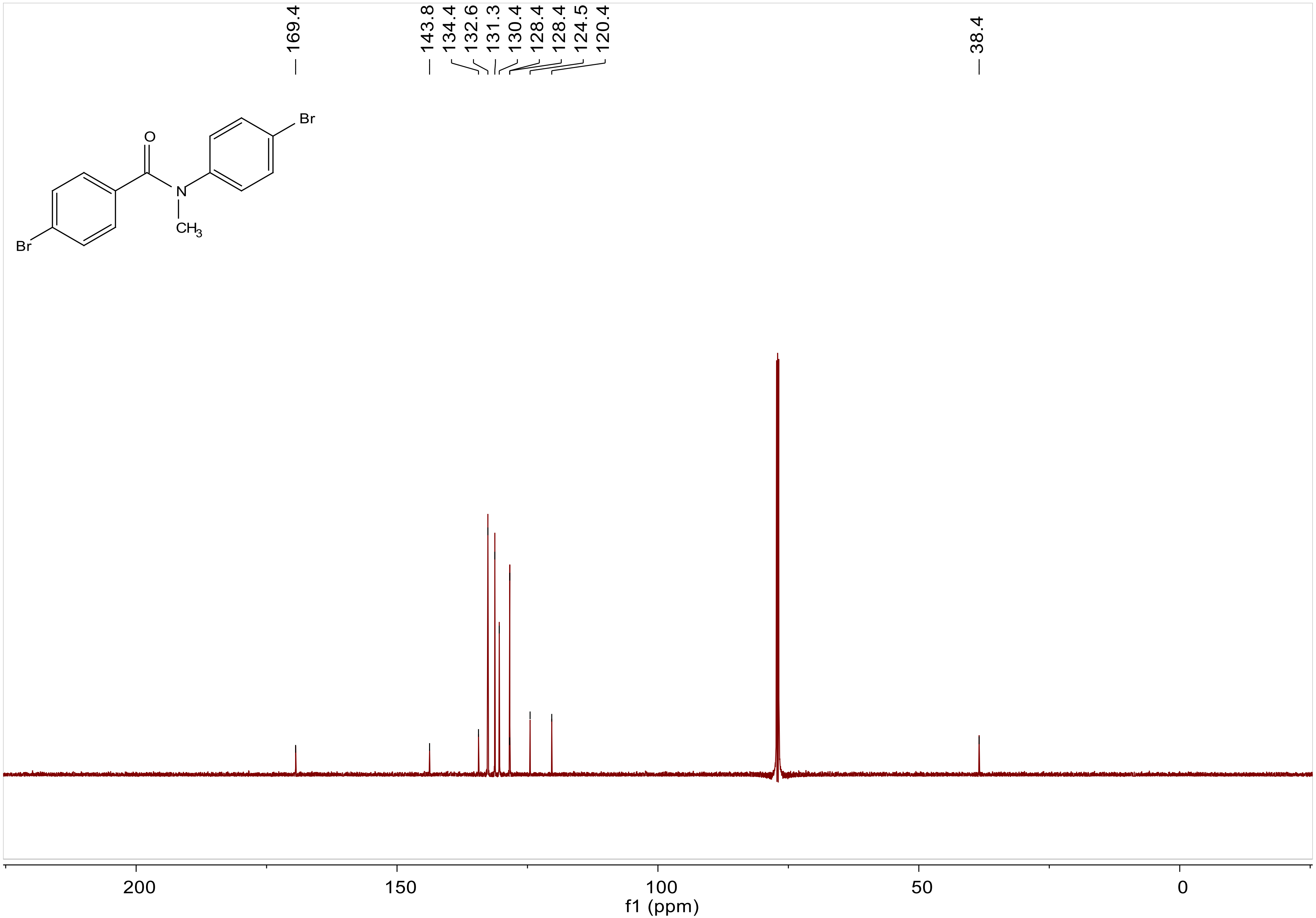

169.4
143.8
134.4
132.6
131.3
130.4
128.4
128.4
124.5
120.4
38.4
Br
O
N
CH3
Br
200
150
100
50
0
f1 (ppm)

NMR data of compound *N*-(4-bromophenyl)-4-chloro-*N*-methylbenzamide

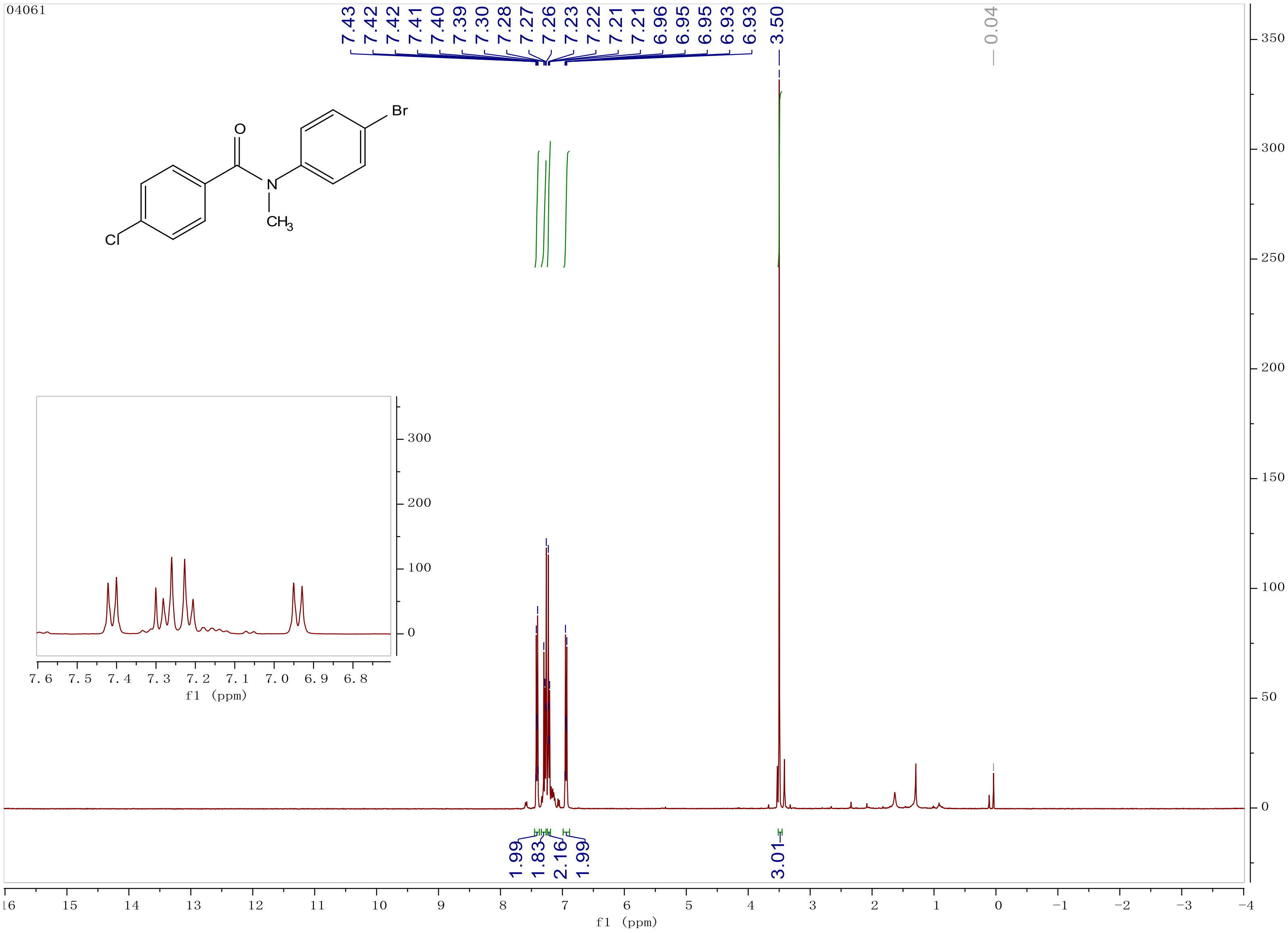

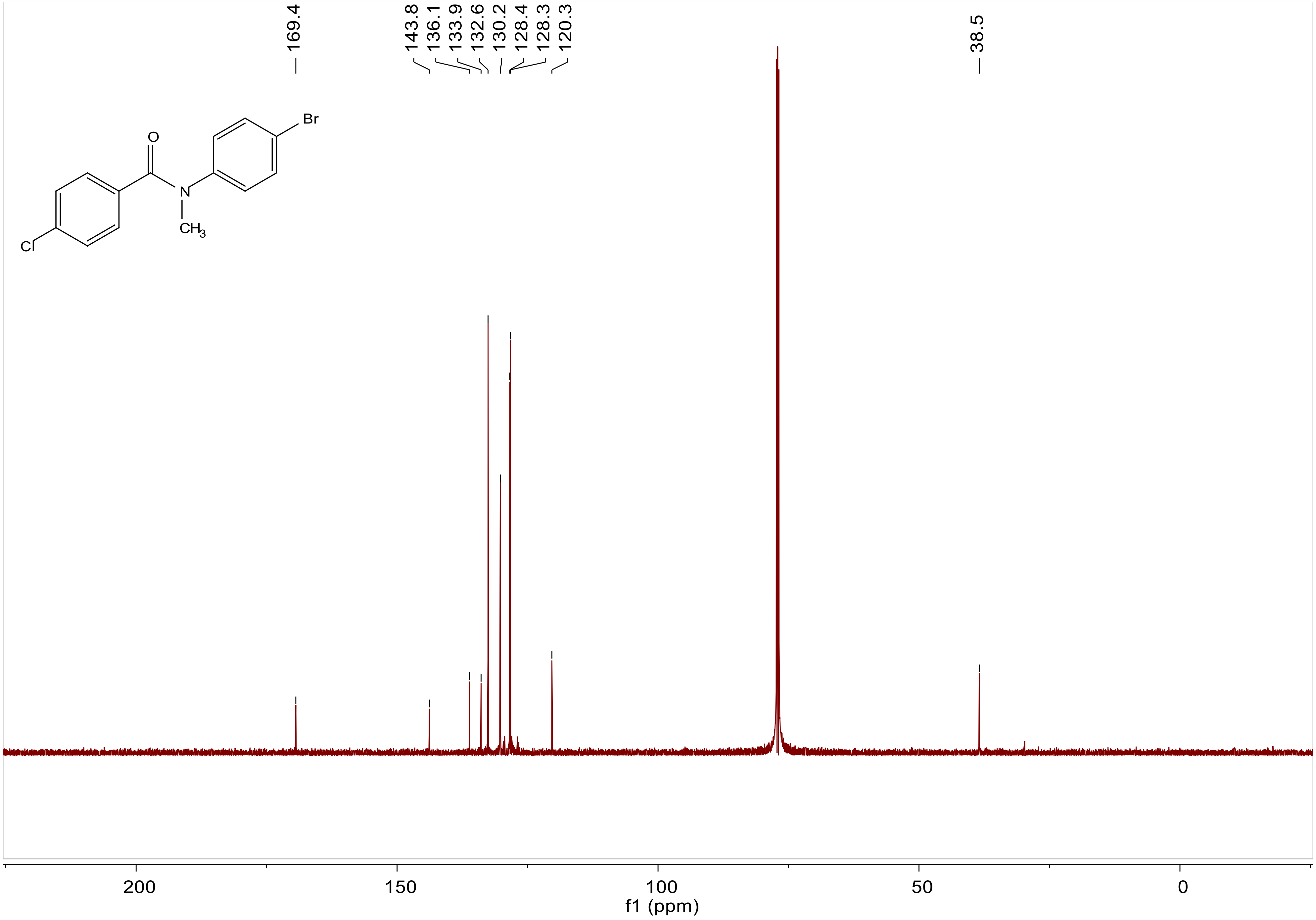
169.4
143.8
136.1
133.9
132.6
130.2
128.4
128.3
120.3
38.5
Br
O
N
CH3
Cl
200
150
100
50
0
f1 (ppm)

NMR data of compound 3-bromo-*N*-(4-bromophenyl)-*N*-methylbenzamide

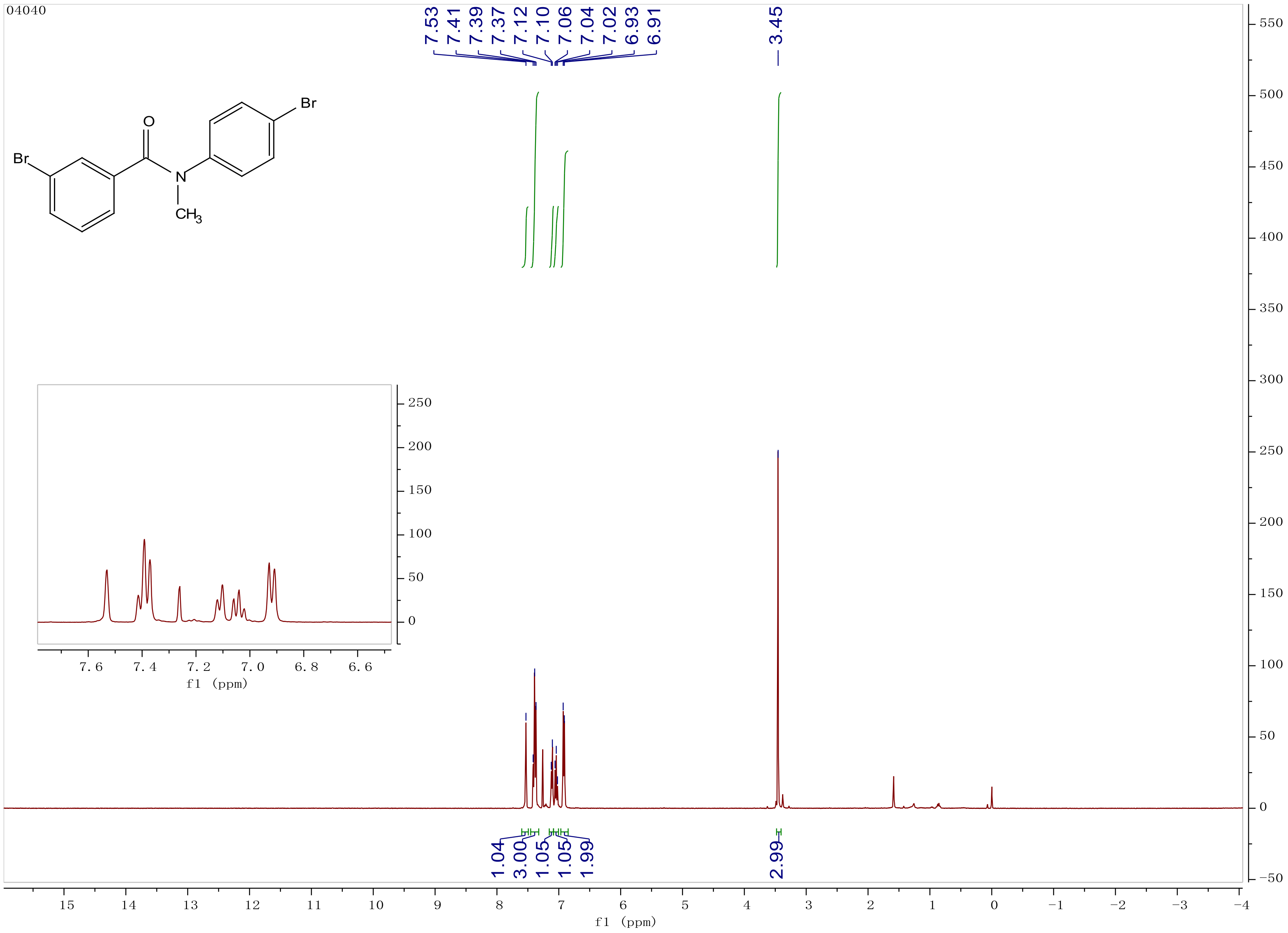

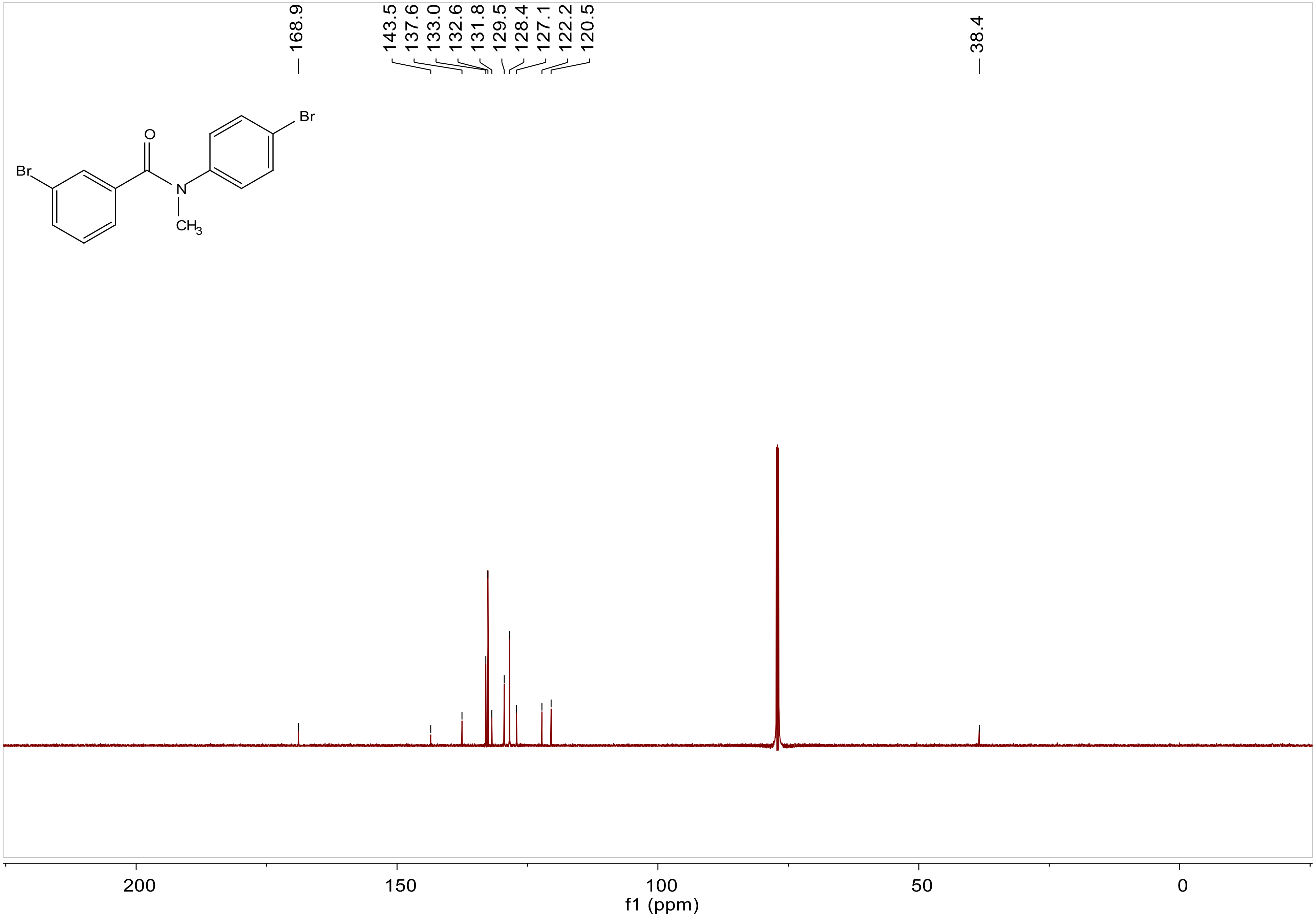
168.9
143.5
137.6
133.0
132.6
131.8
129.5
128.4
127.1
122.2
120.5
38.4
Br
O
Br
N
CH3
200
150
100
50
0
f1 (ppm)

NMR data of compound *N*-(4-bromophenyl)-3-fluoro-*N*-methylbenzamide

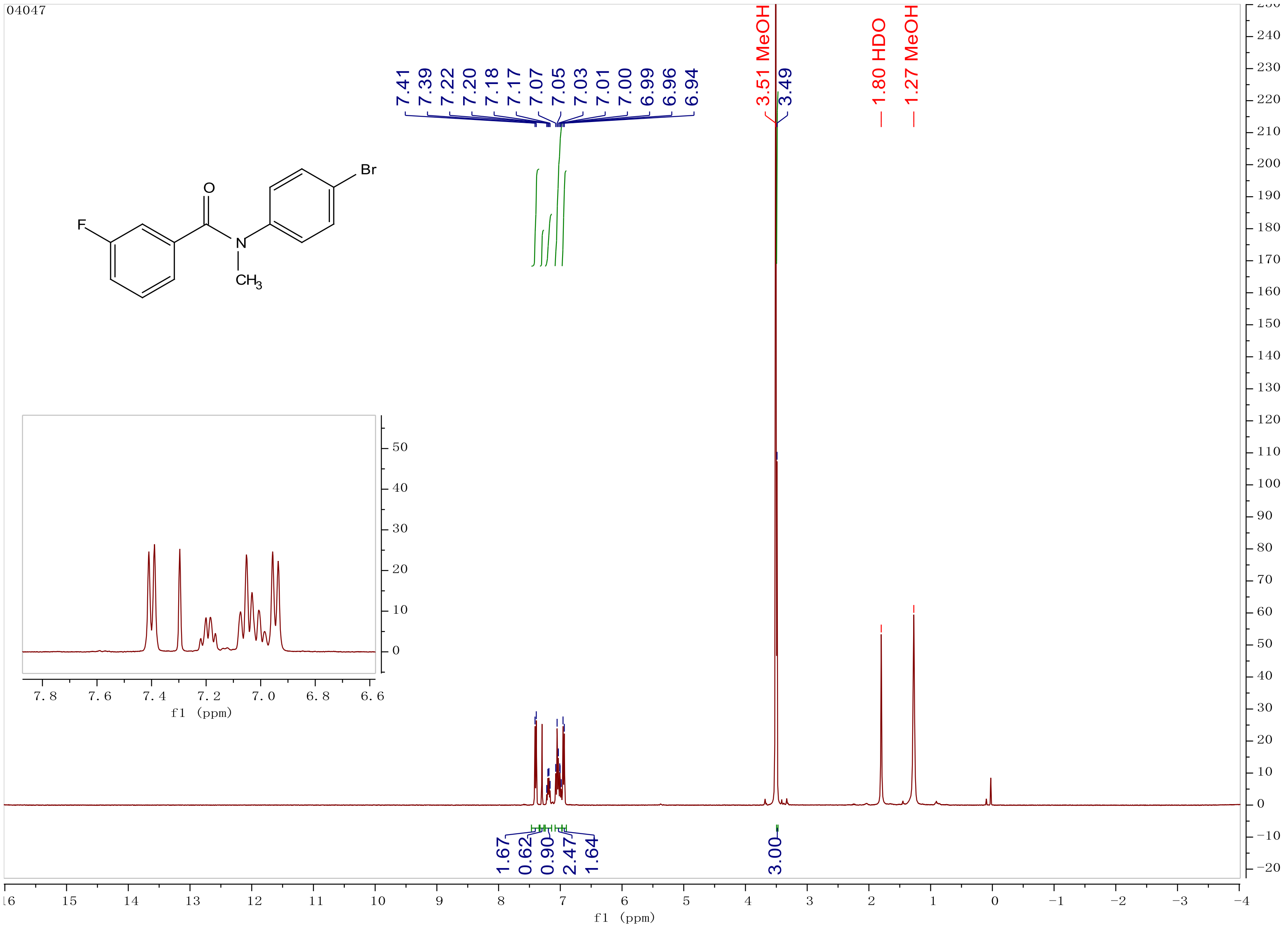

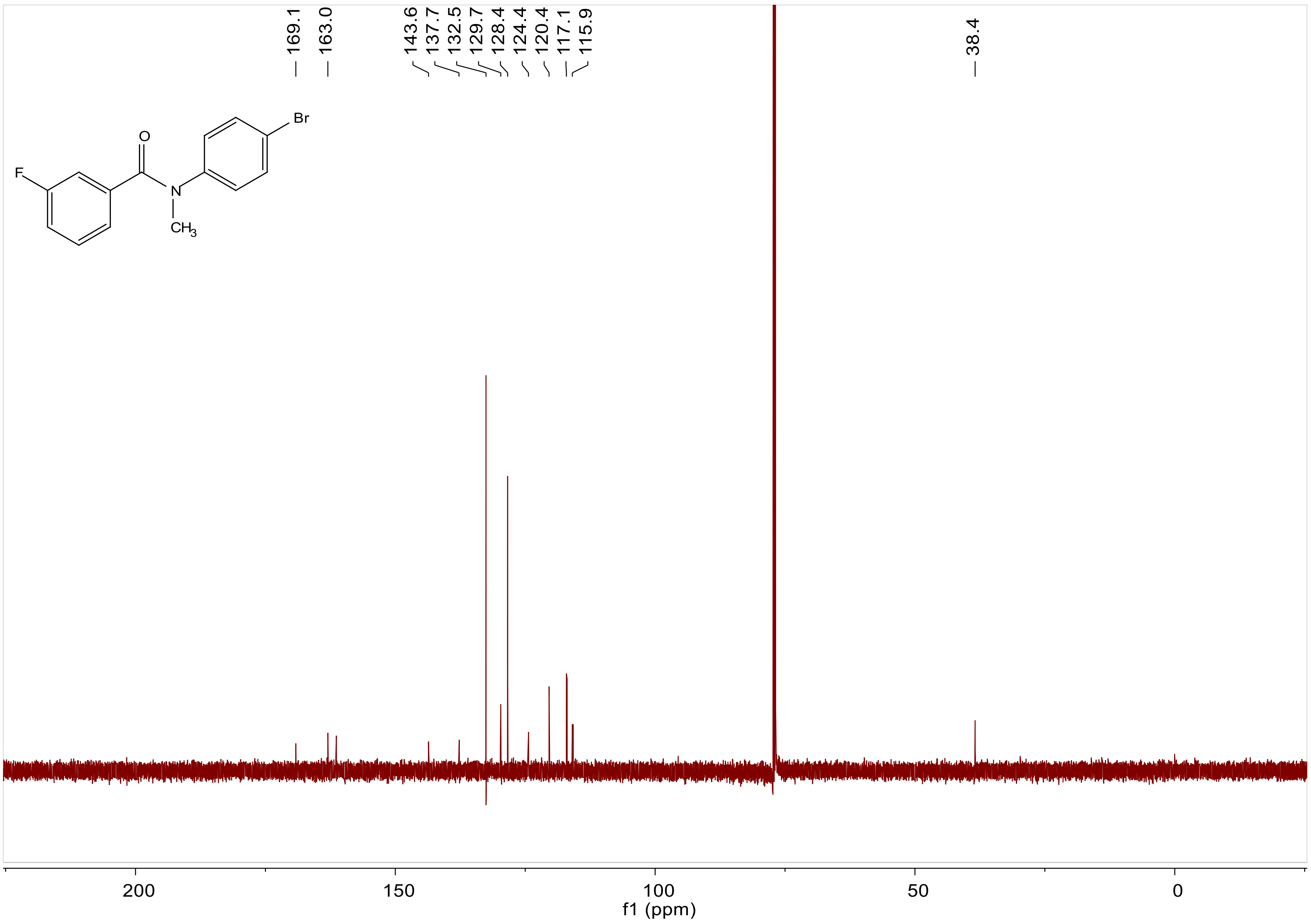
169.1
163.0
143.6
137.7
132.5
129.7
128.4
124.4
120.4
117.1
115.9
38.4
Br
O
F
N
CH3
200
150
100
50
0
f1 (ppm)

NMR data of compound *N*-(4-bromophenyl)-2-fluoro-*N*-methylbenzamide

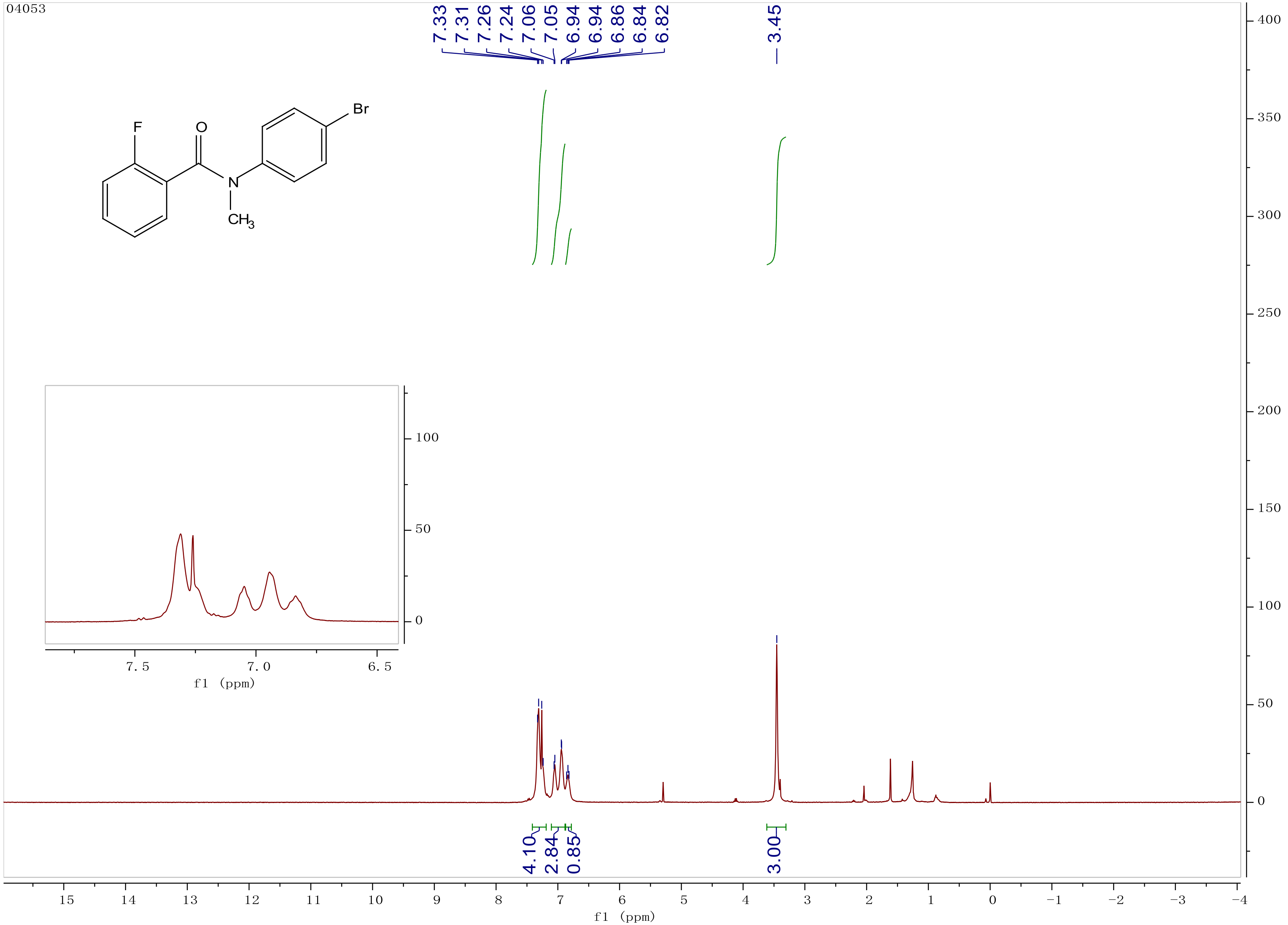

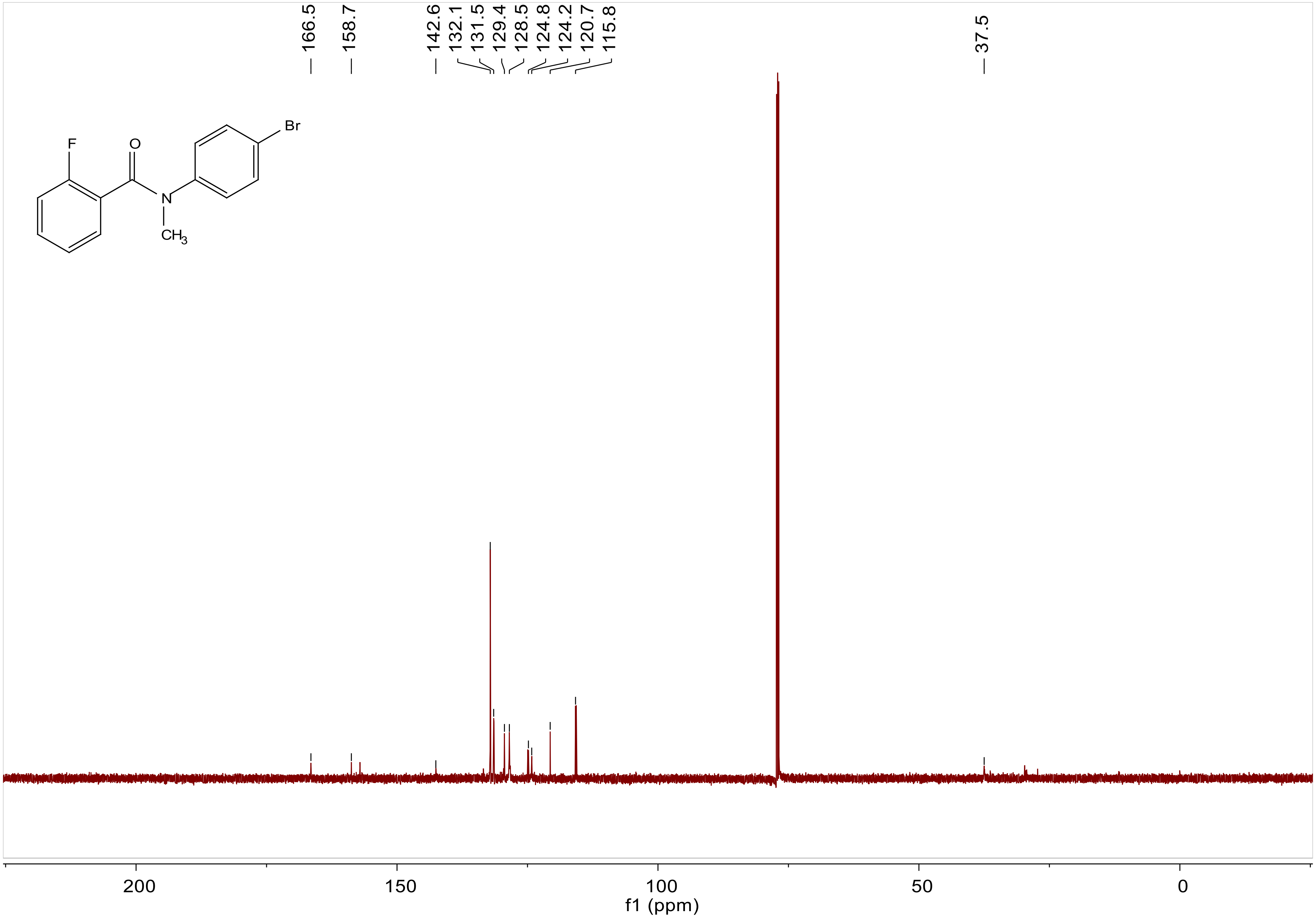
166.5
158.7
142.6
132.1
131.5
129.4
128.5
124.8
124.2
120.7
115.8
37.5
F
O
N
CH3
Br
200
150
100
50
0
f1 (ppm)

NMR data of compound *N*-(4-bromophenyl)-4-iodo-*N*-methylbenzamide

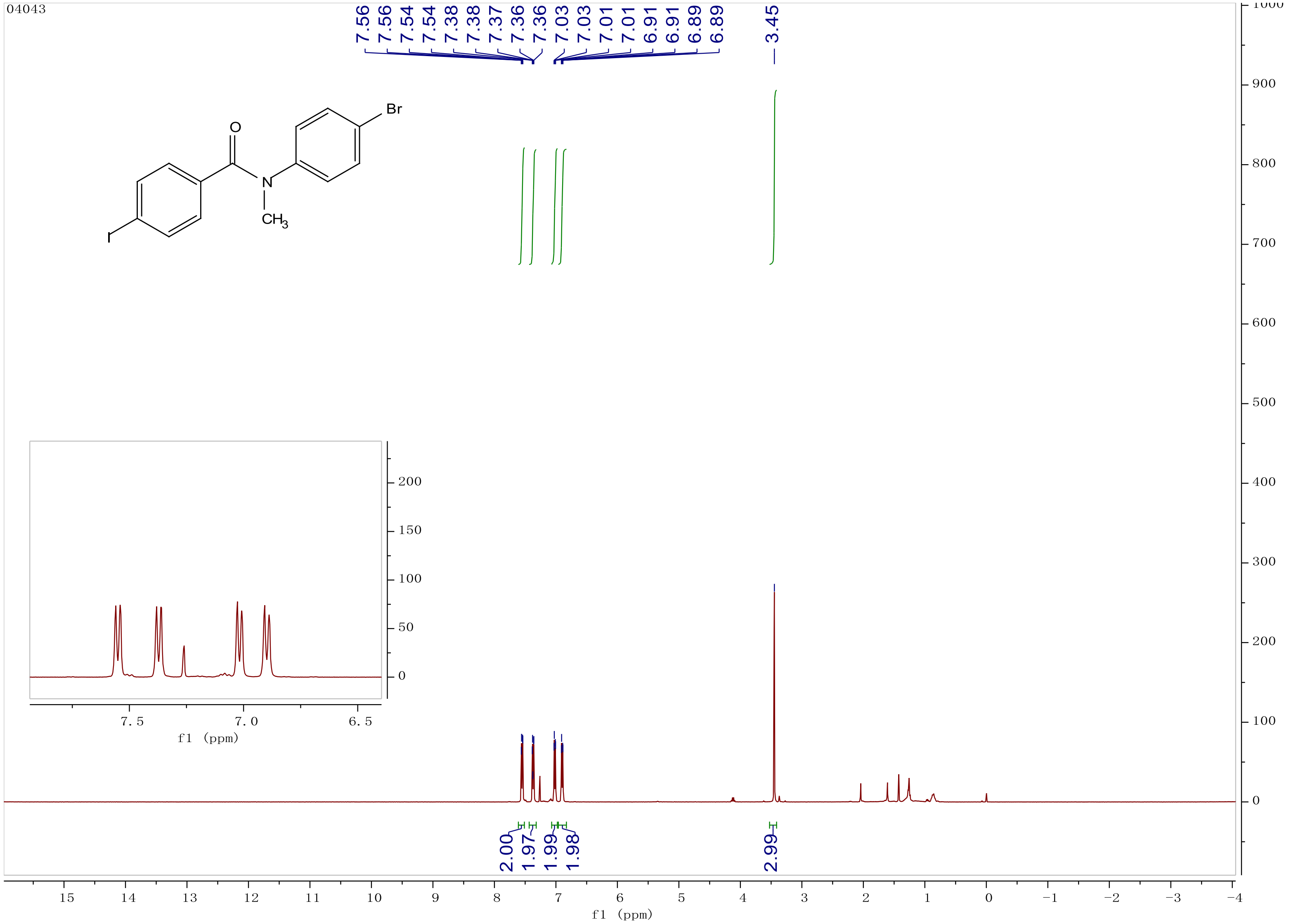

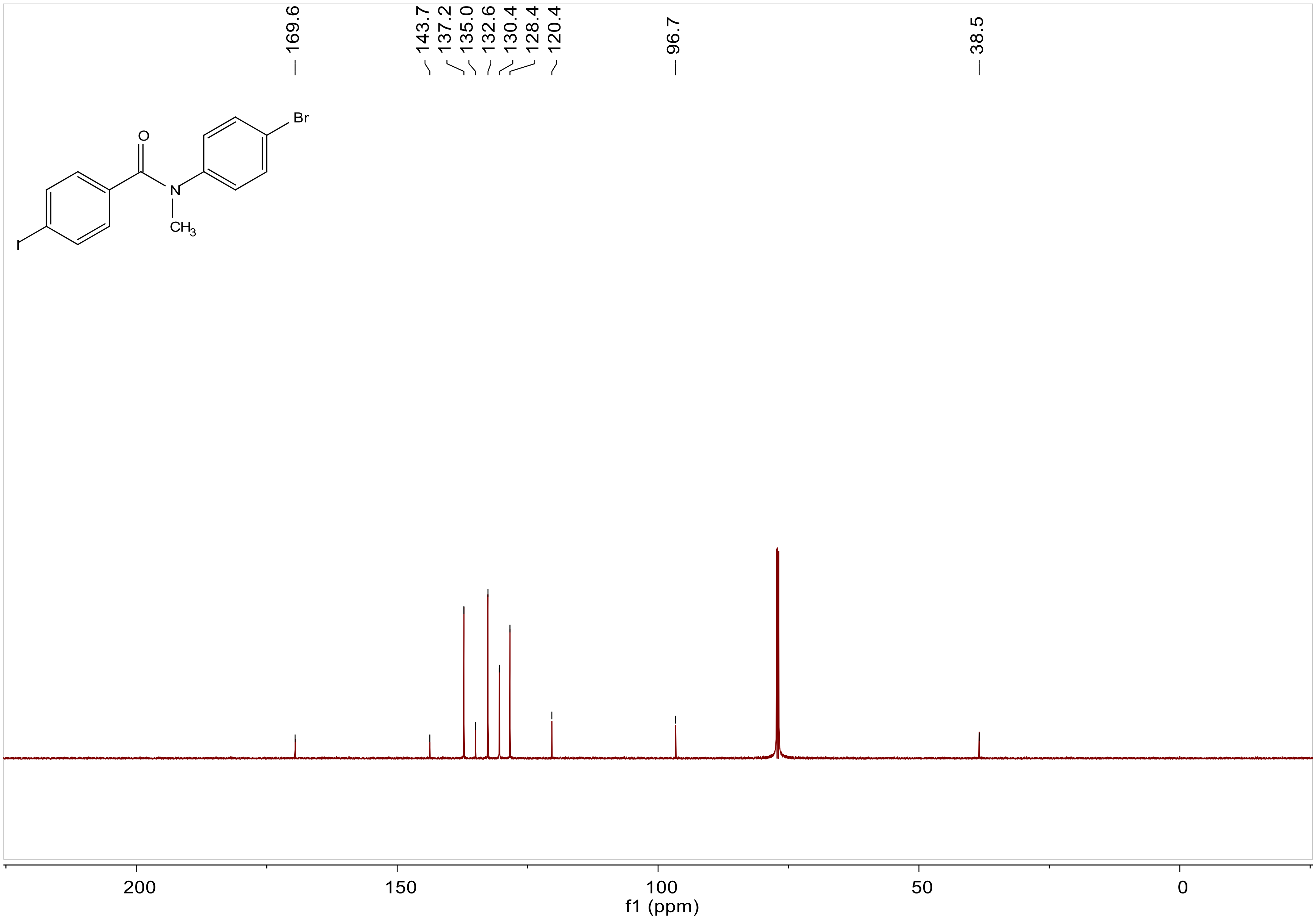
169.6
143.7
137.2
135.0
132.6
130.4
128.4
120.4
96.7
38.5
O
Br
N
CH3
I
200
150
100
50
0
f1 (ppm)

NMR data of compound *N*-(4-bromophenyl)-2-chloro-*N*-methylacetamide

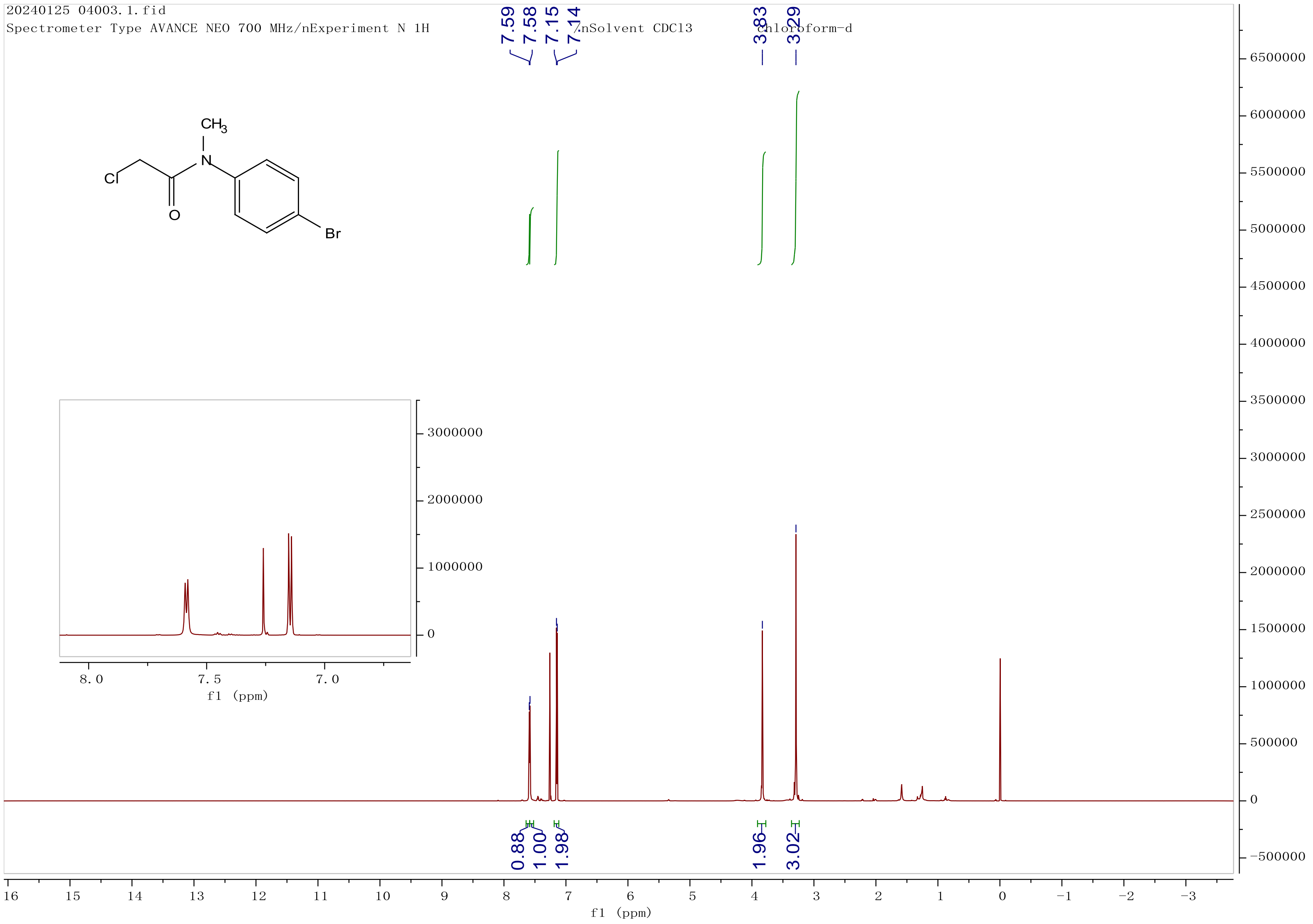

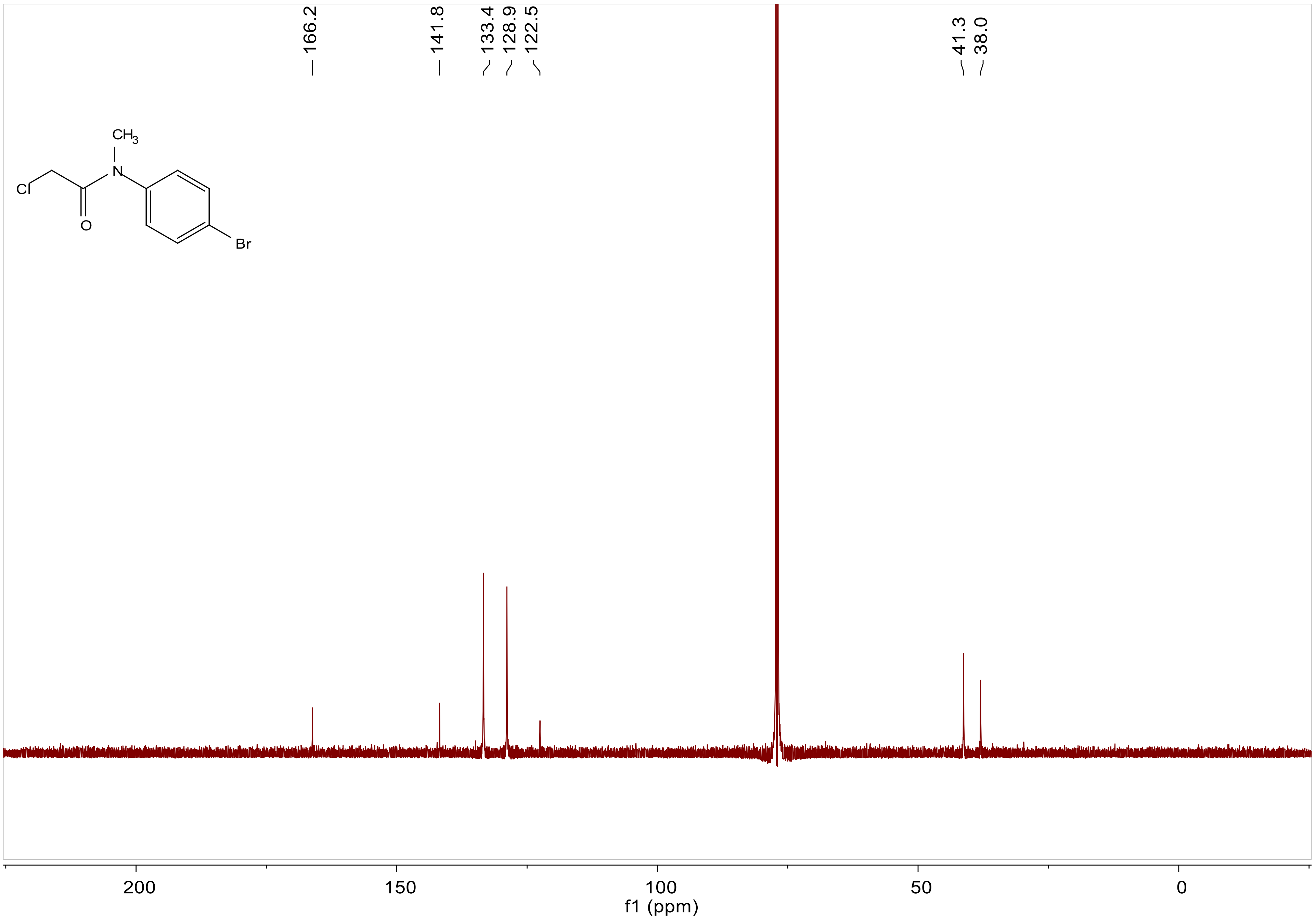

166.2
141.8
133.4
128.9
122.5
41.3
38.0
CH3
N
Cl
O
Br
200
150
100
50
0
f1 (ppm)

NMR data of compound *N*-(4-bromophenyl)-*N*-methyl-4-nitrobenzamide

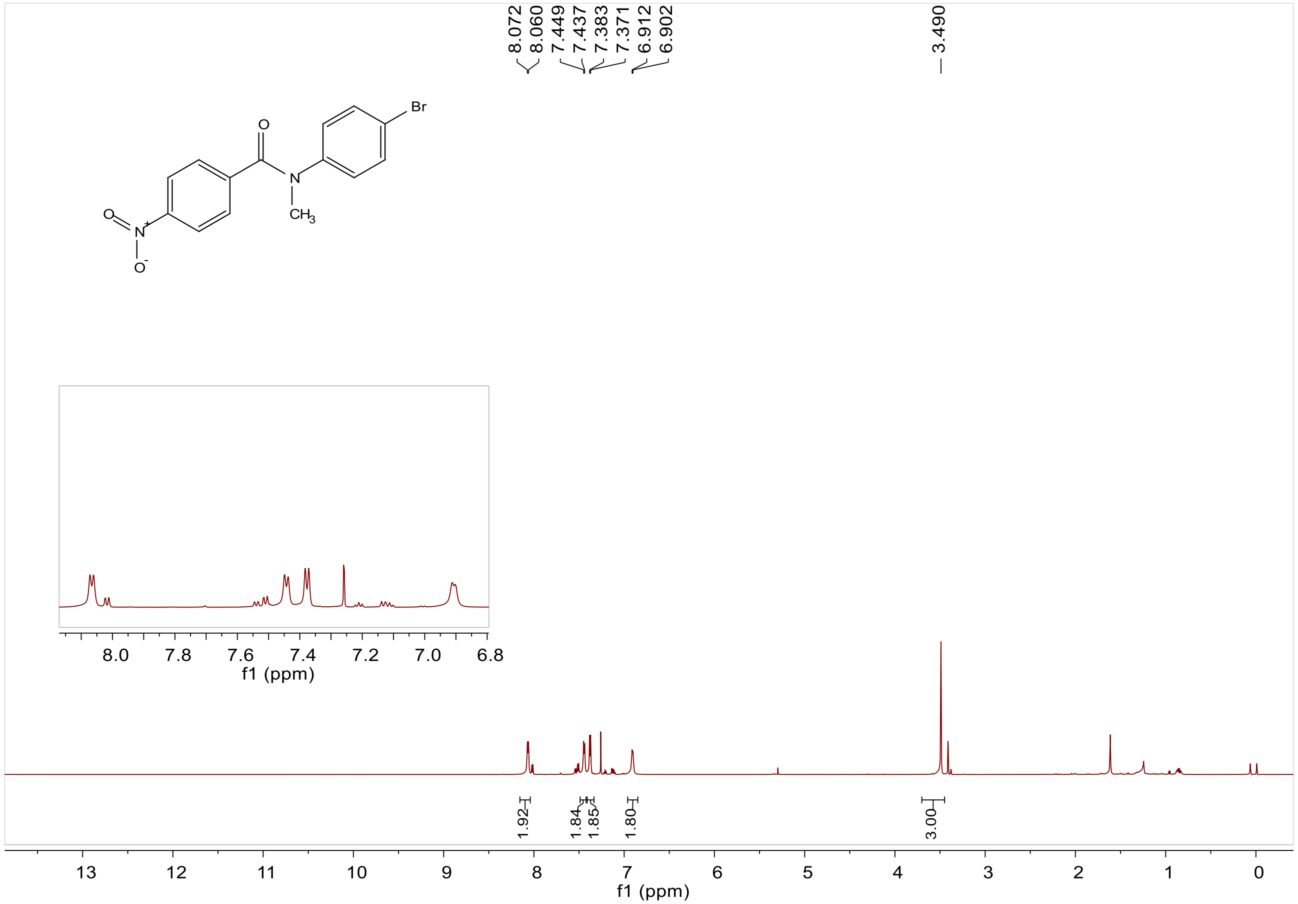

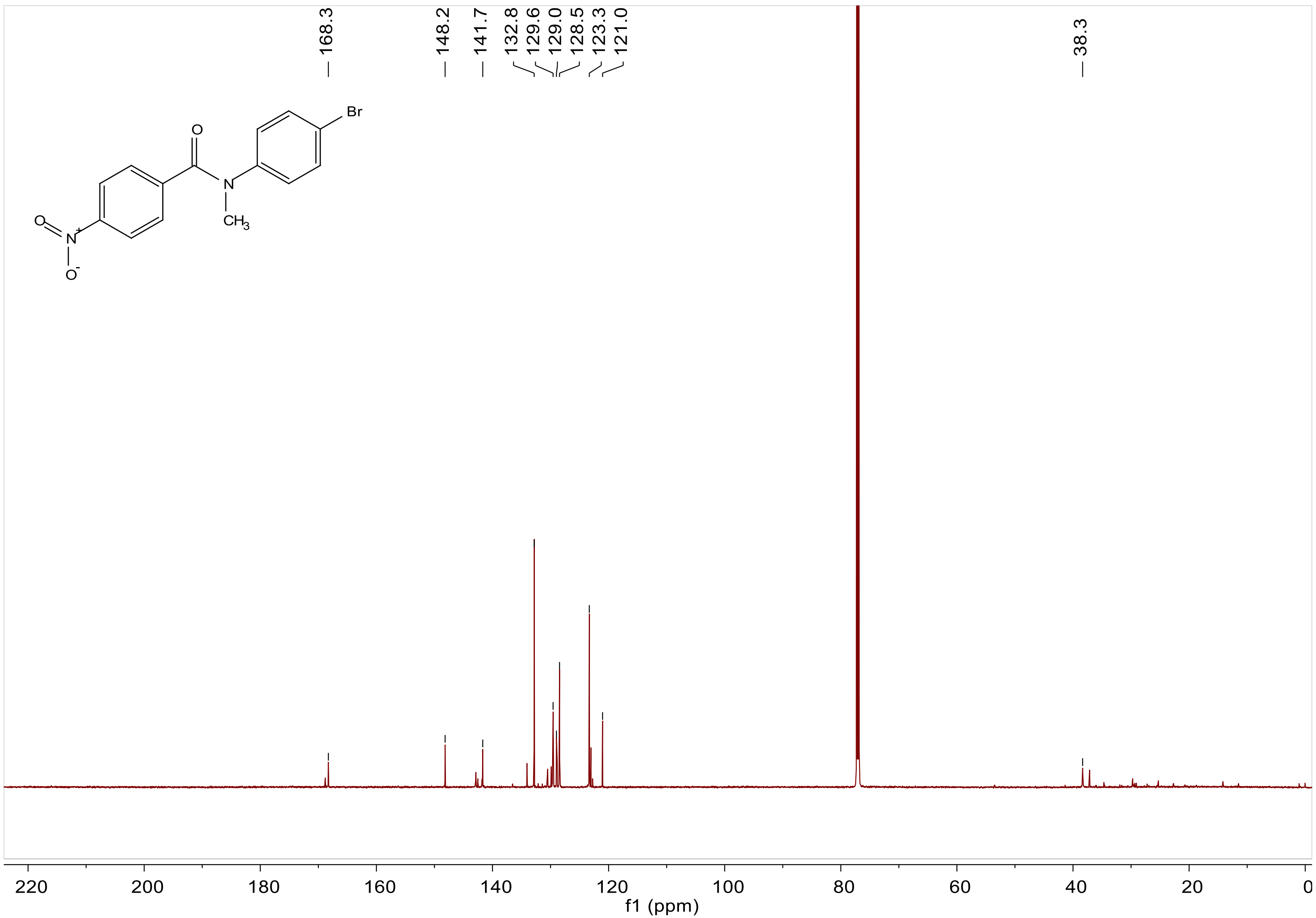

168.3
148.2
141.7
132.8
129.6
129.0
128.5
123.3
121.0
38.3
Br
O
N
CH3
O
N+
O-
220
200
180
160
140
120
100
80
60
40
20
0
f1 (ppm)

NMR data of compound 3-bromo-*N*-methyl-*N*-phenylbenzamide

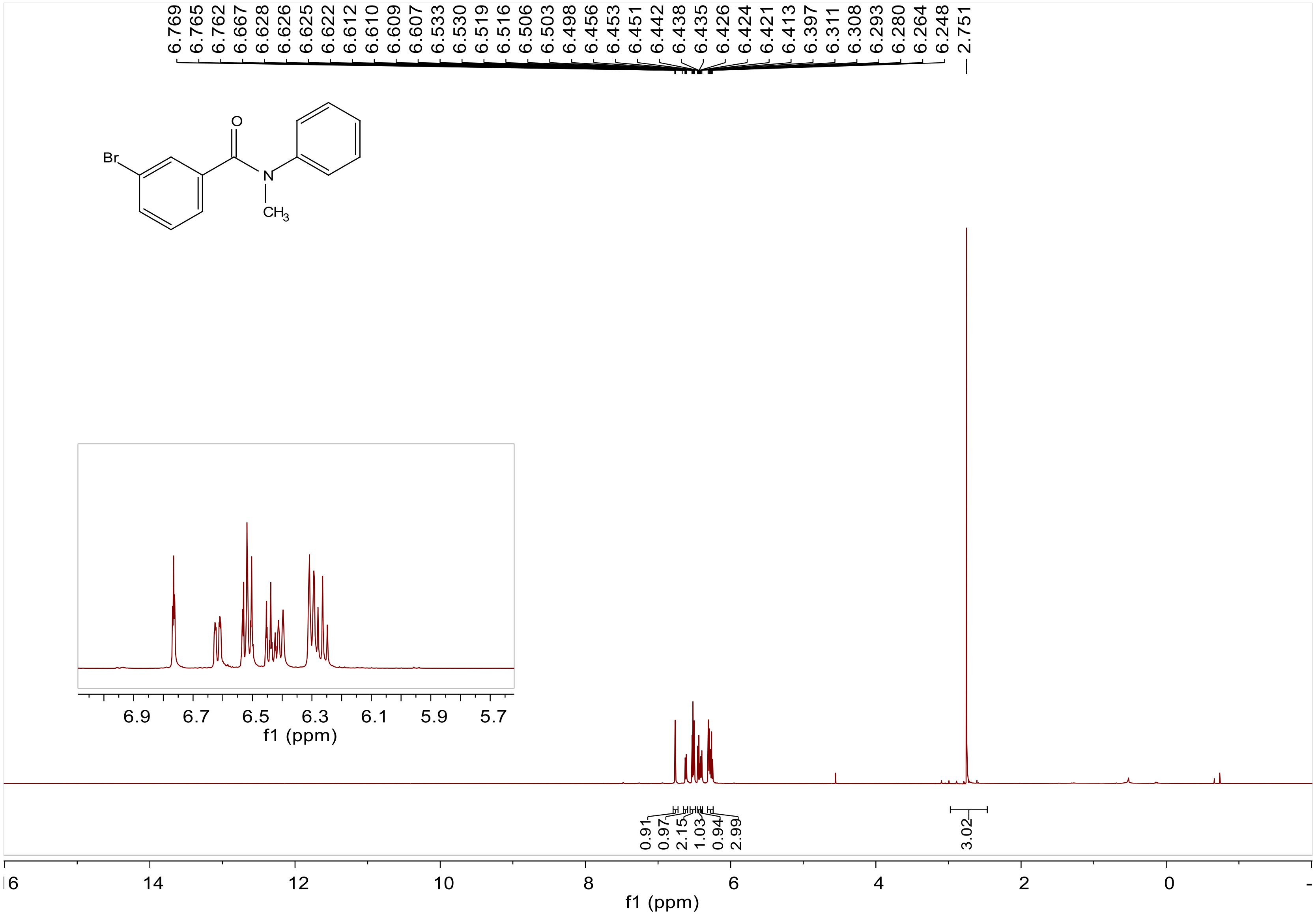

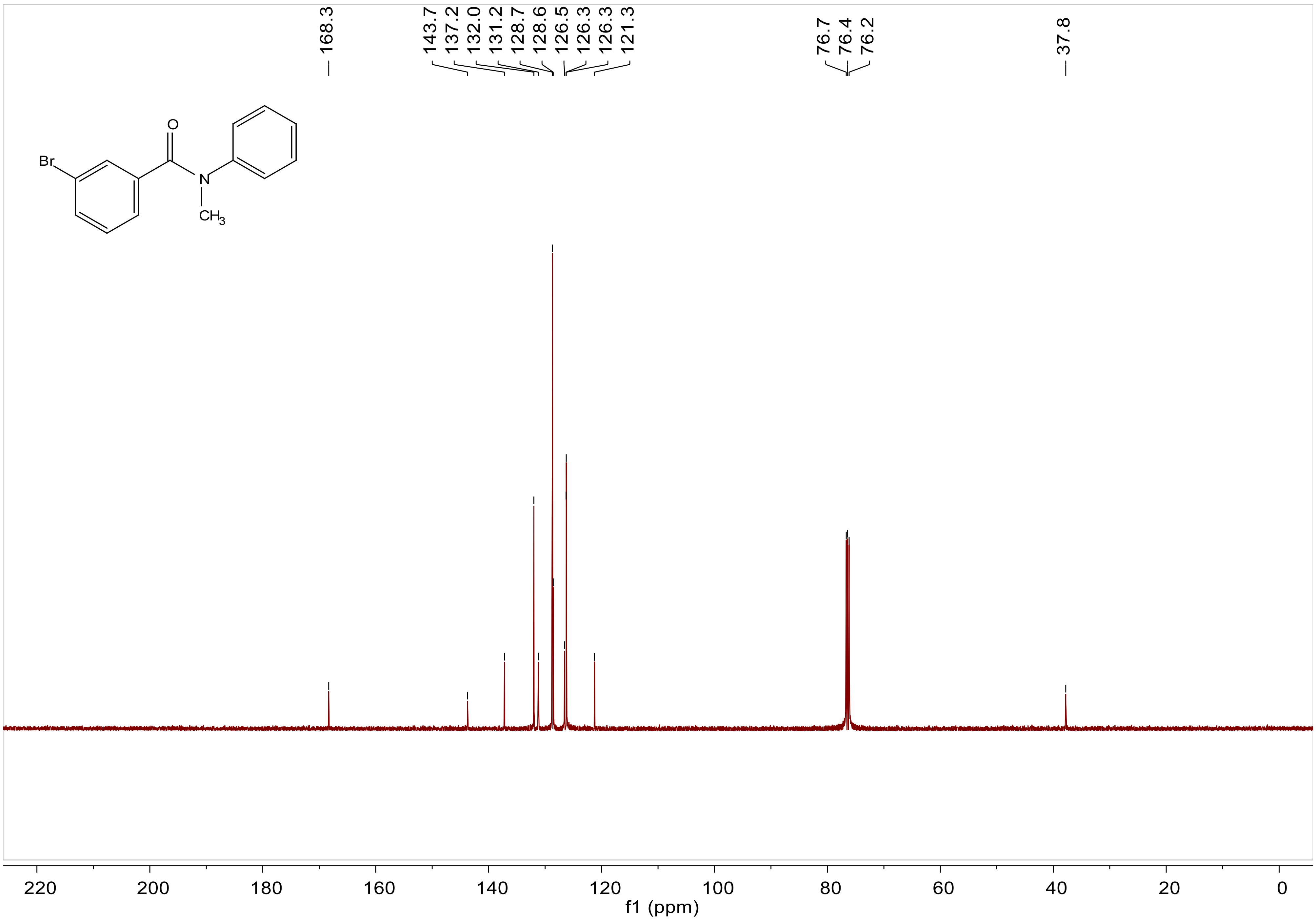
168.3
143.7
137.2
132.0
131.2
128.7
128.6
126.5
126.3
126.3
121.3
76.7
76.4
76.2
37.8
Br
O
N
CH3
220
200
180
160
140
120
100
80
60
40
20
0
f1 (ppm)

NMR data of compound 3-fluoro-*N*-methyl-*N*-phenylbenzamide

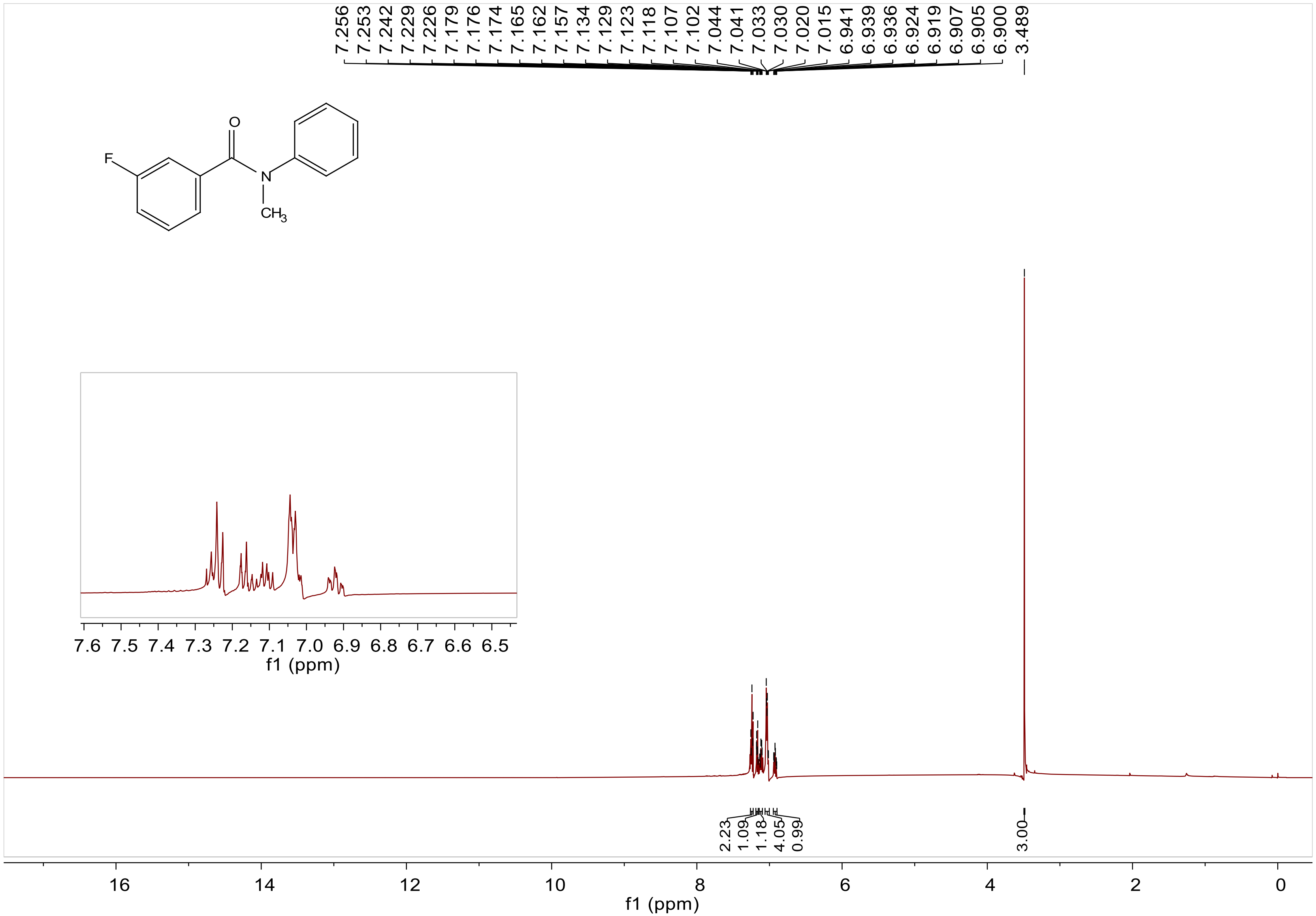

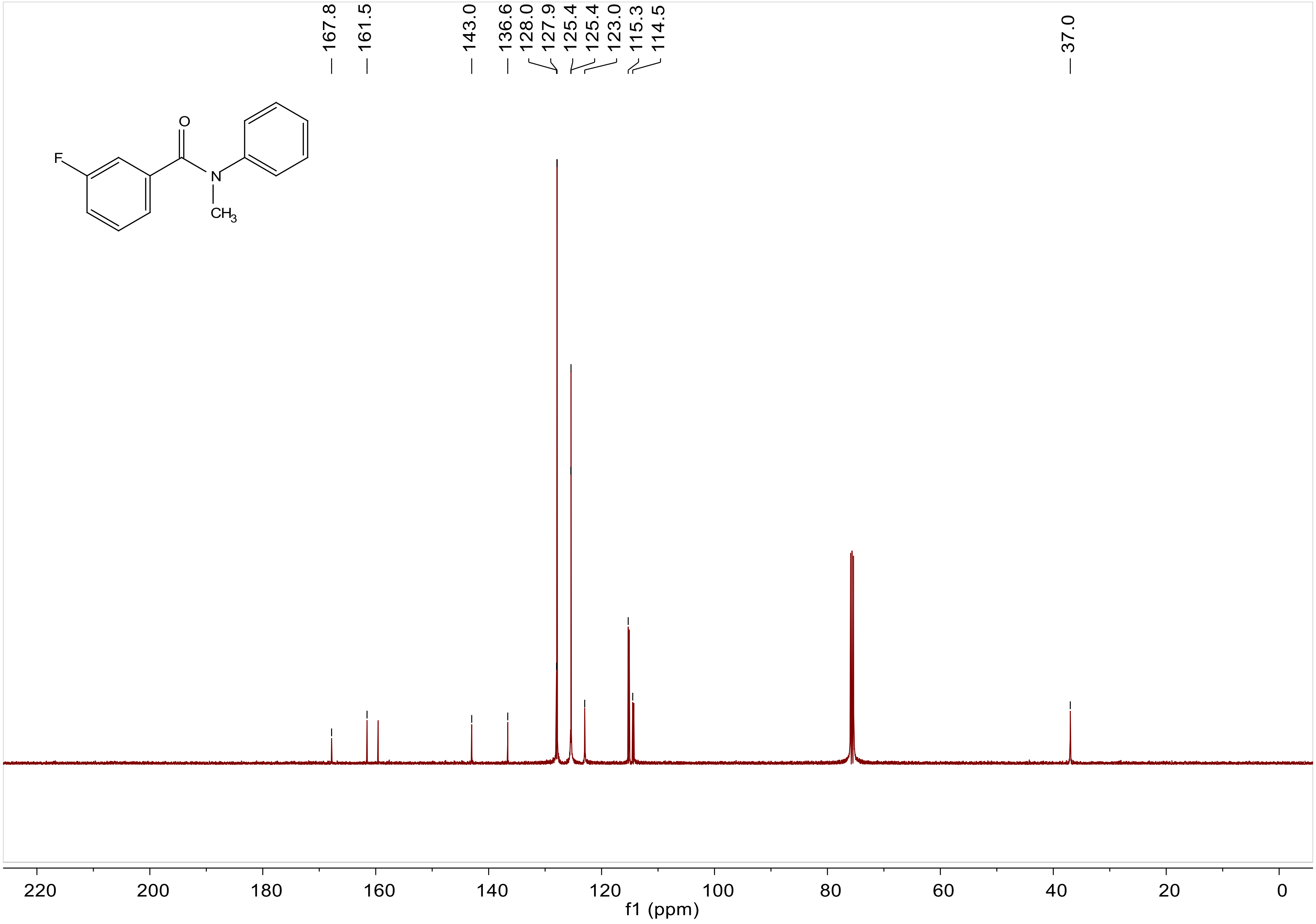

167.8
161.5
143.0
136.6
128.0
127.9
125.4
125.4
123.0
115.3
114.5
37.0
F
O
N
CH3
220
200
180
160
140
120
100
80
60
40
20
0
f1 (ppm)

NMR data of compound 2-fluoro-*N*-methyl-*N*-phenylbenzamide

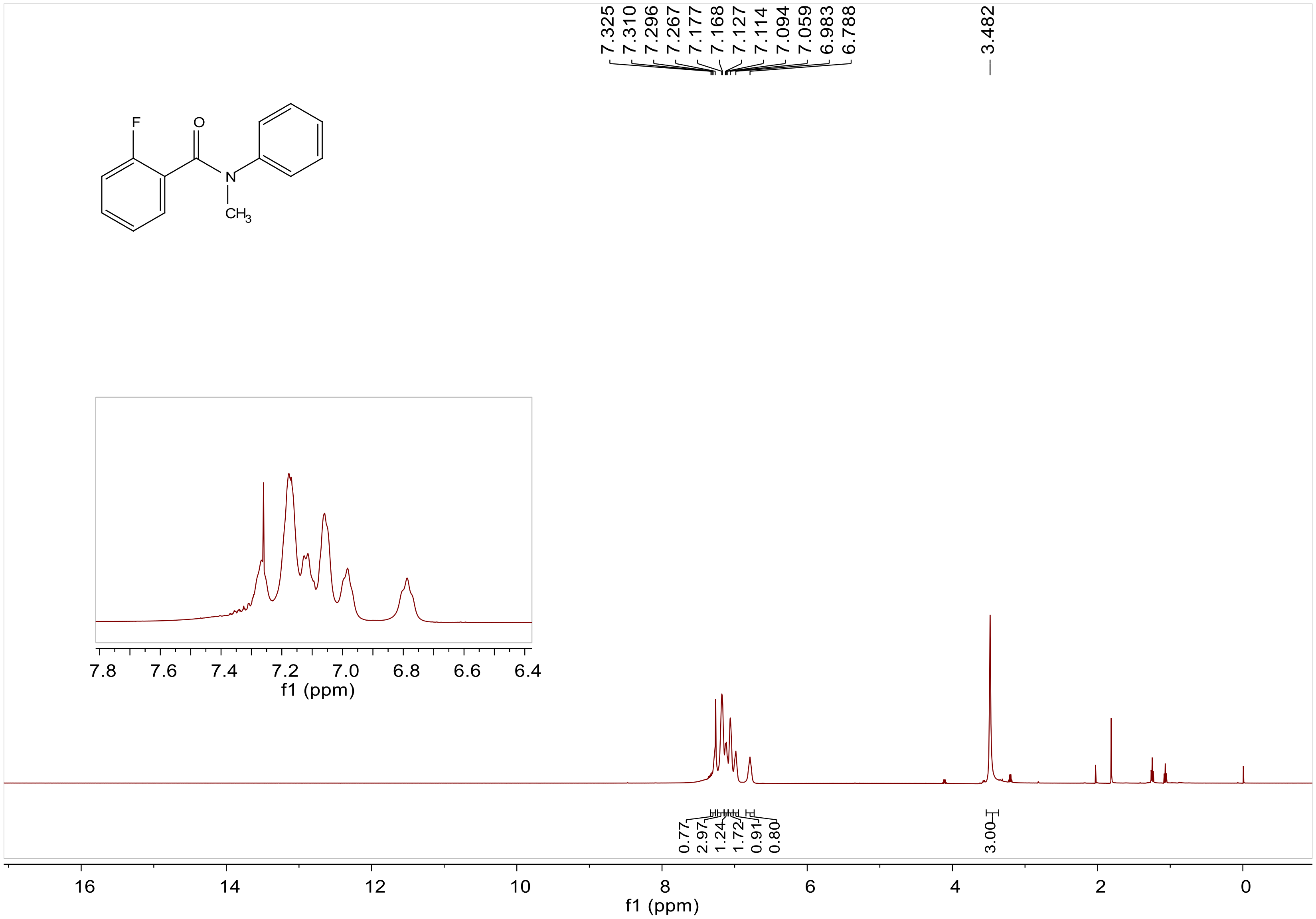

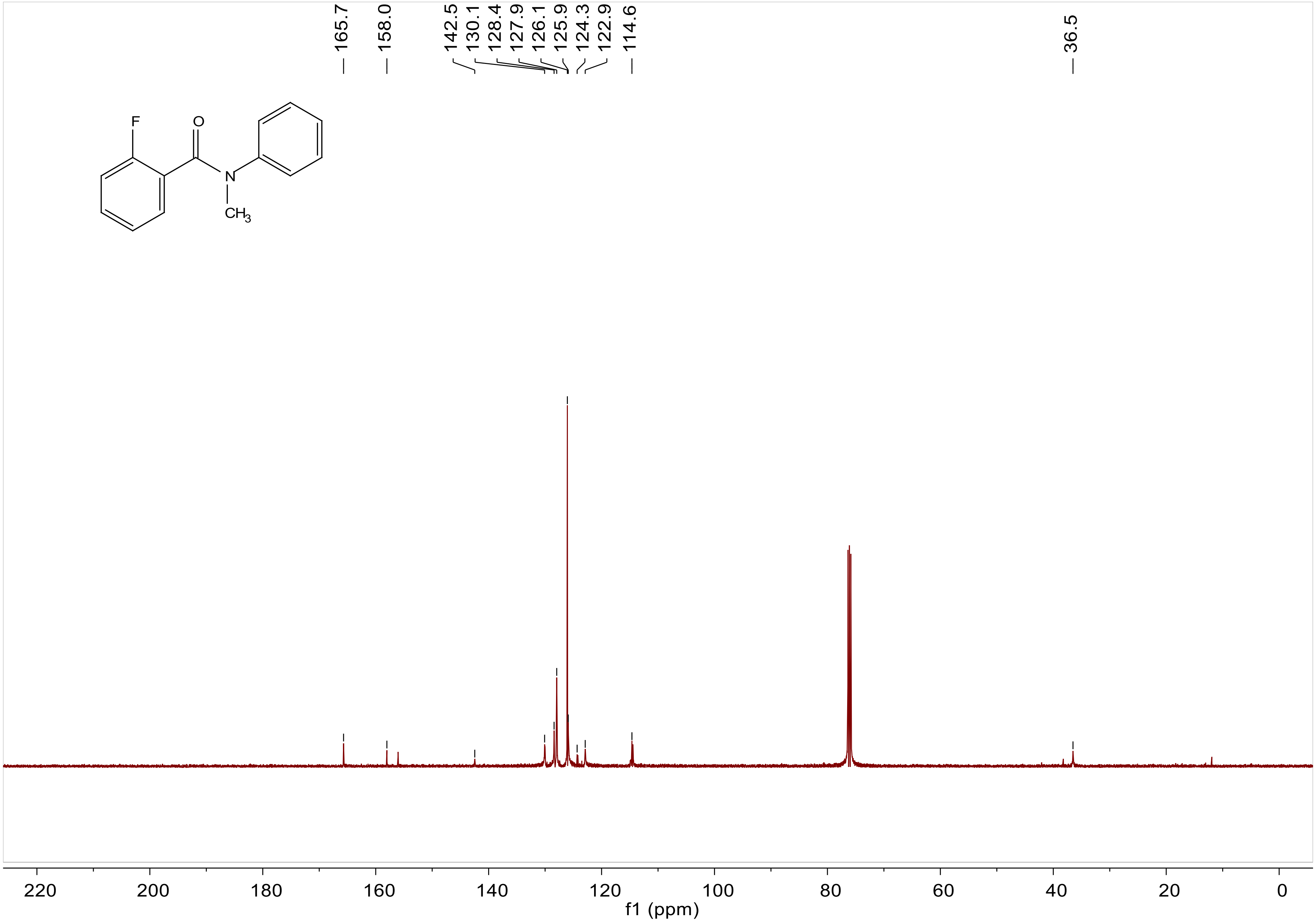

165.7
158.0
142.5
130.1
128.4
127.9
126.1
125.9
124.3
122.9
114.6
36.5
F
O
N
CH3
220
200
180
160
140
120
100
80
60
40
20
0
f1 (ppm)

NMR data of compound 4-iodo -*N*-methyl-*N*-phenylbenzamide

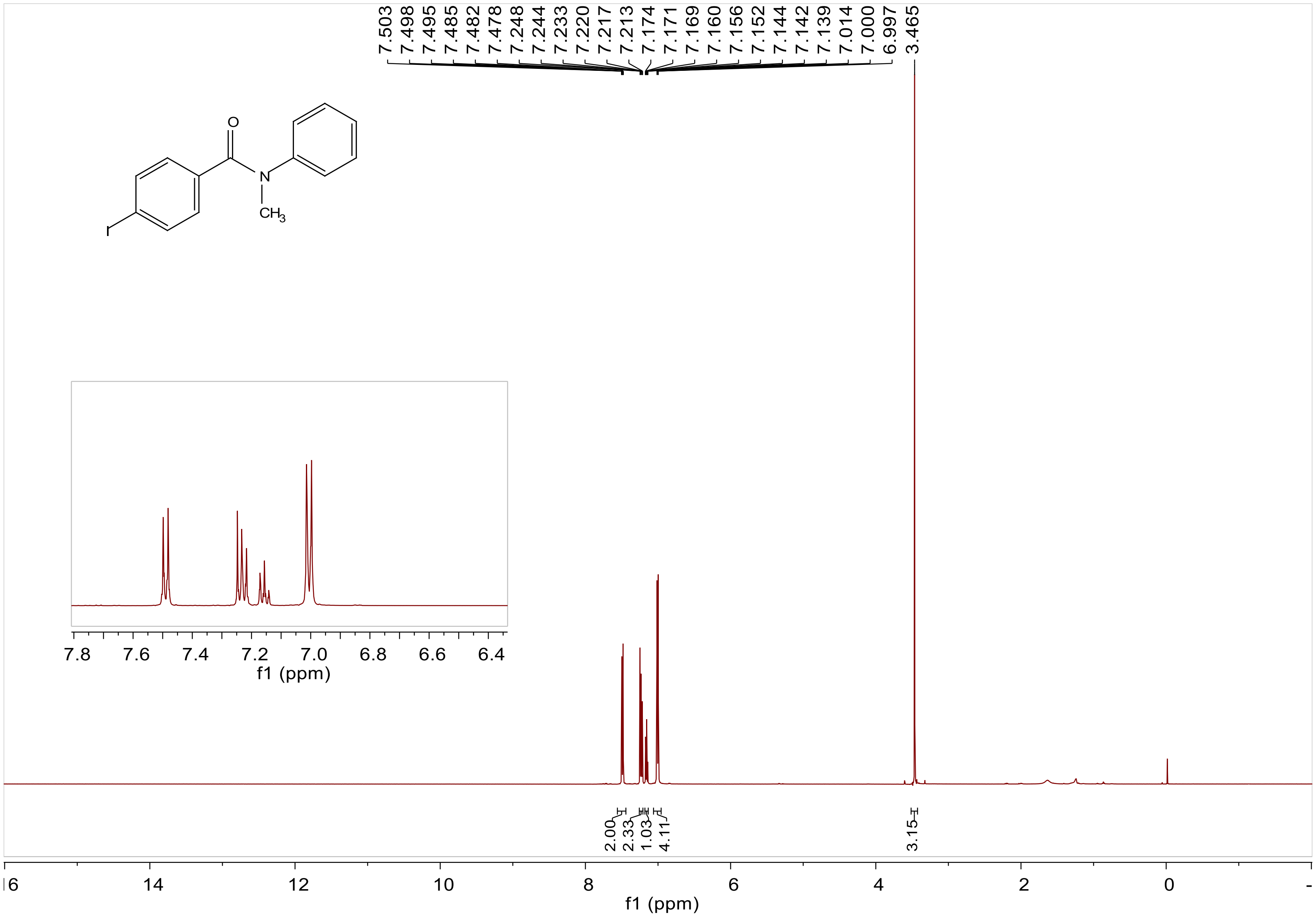

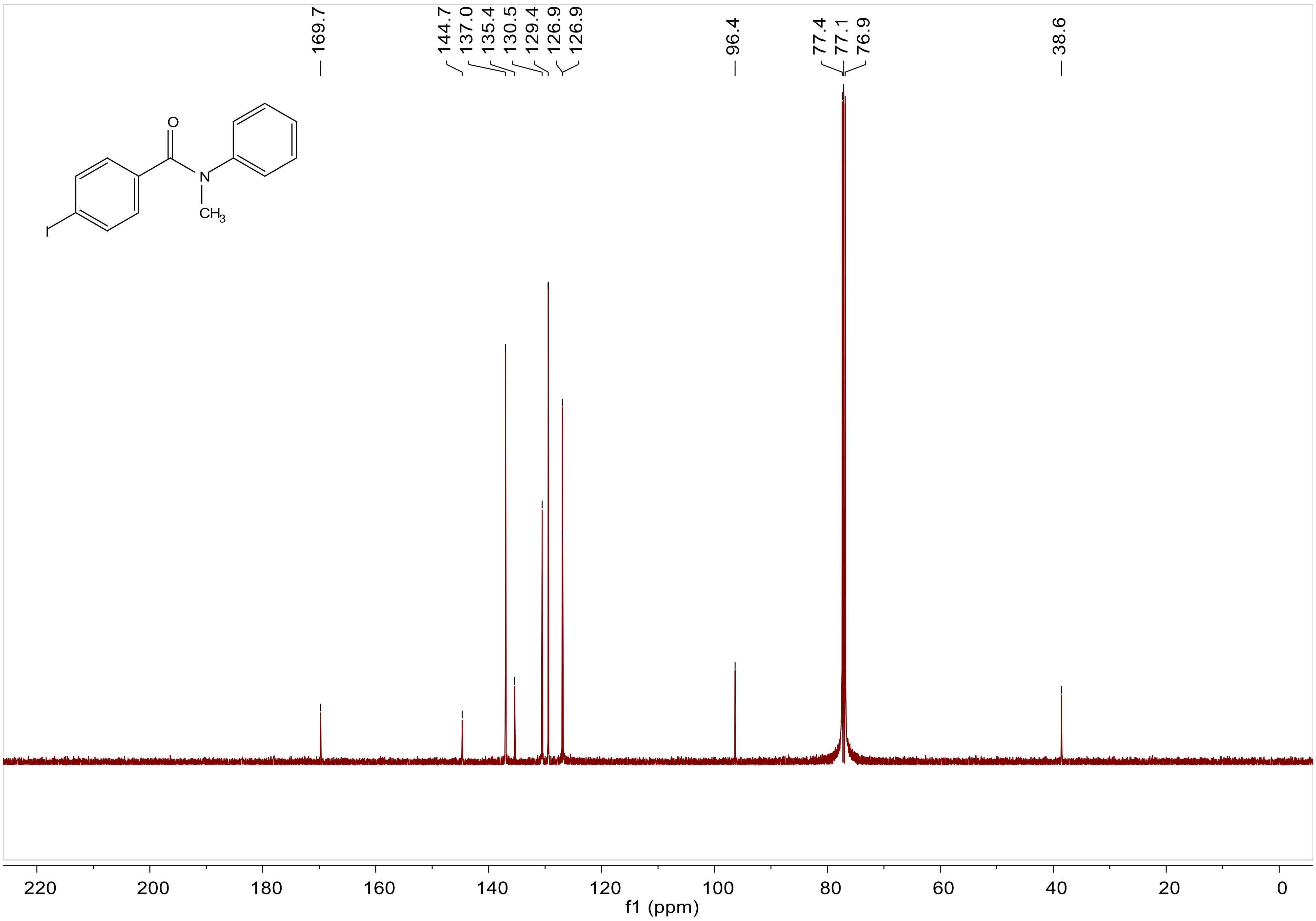

169.7
144.7
137.0
135.4
130.5
129.4
126.9
126.9
96.4
77.4
77.1
76.9
38.6
O
N
CH3
I
220
200
180
160
140
120
100
80
60
40
20
0
f1 (ppm)

NMR data of compound 4-chloro-*N*-methyl-*N*-phenylbenzamide

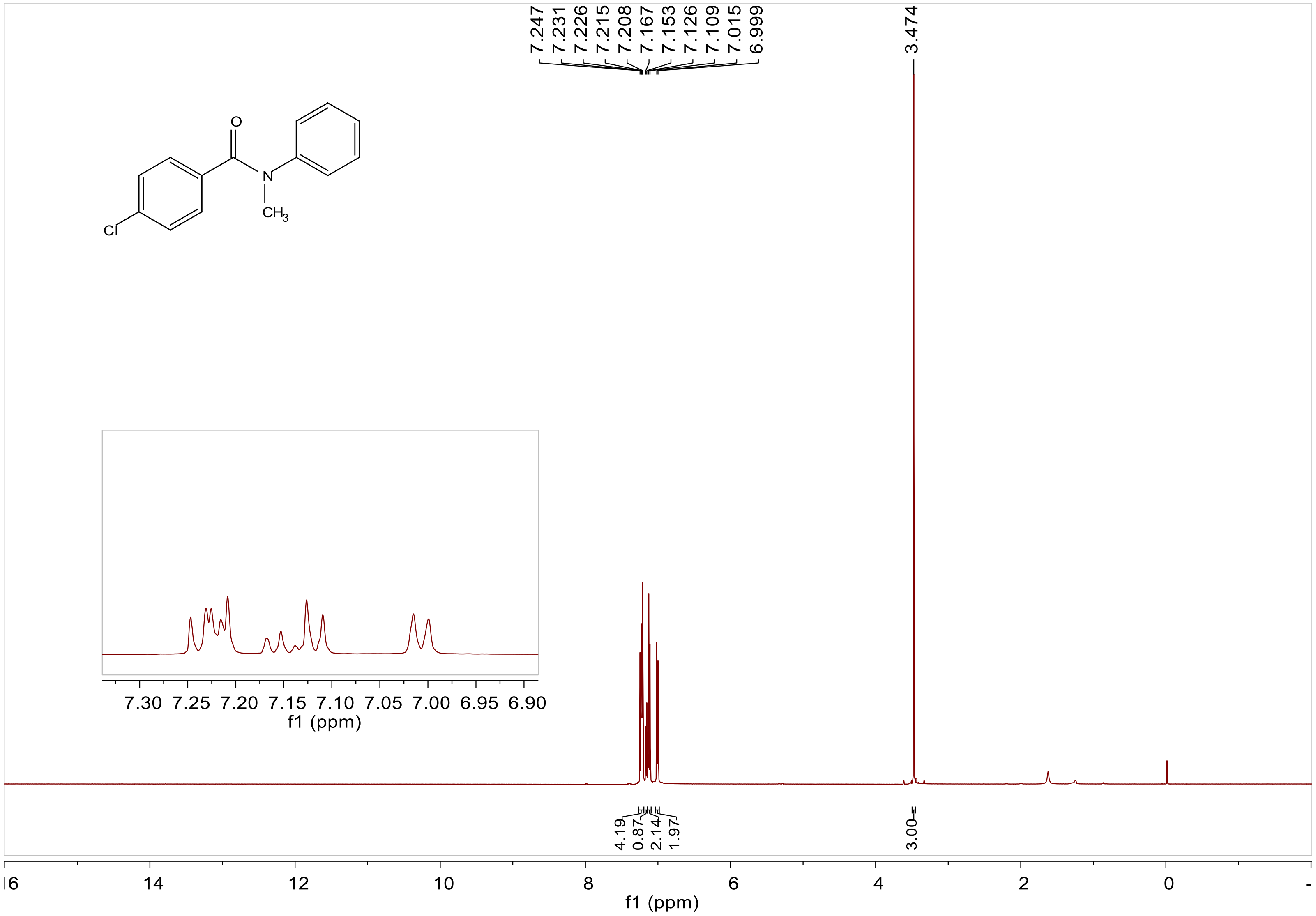

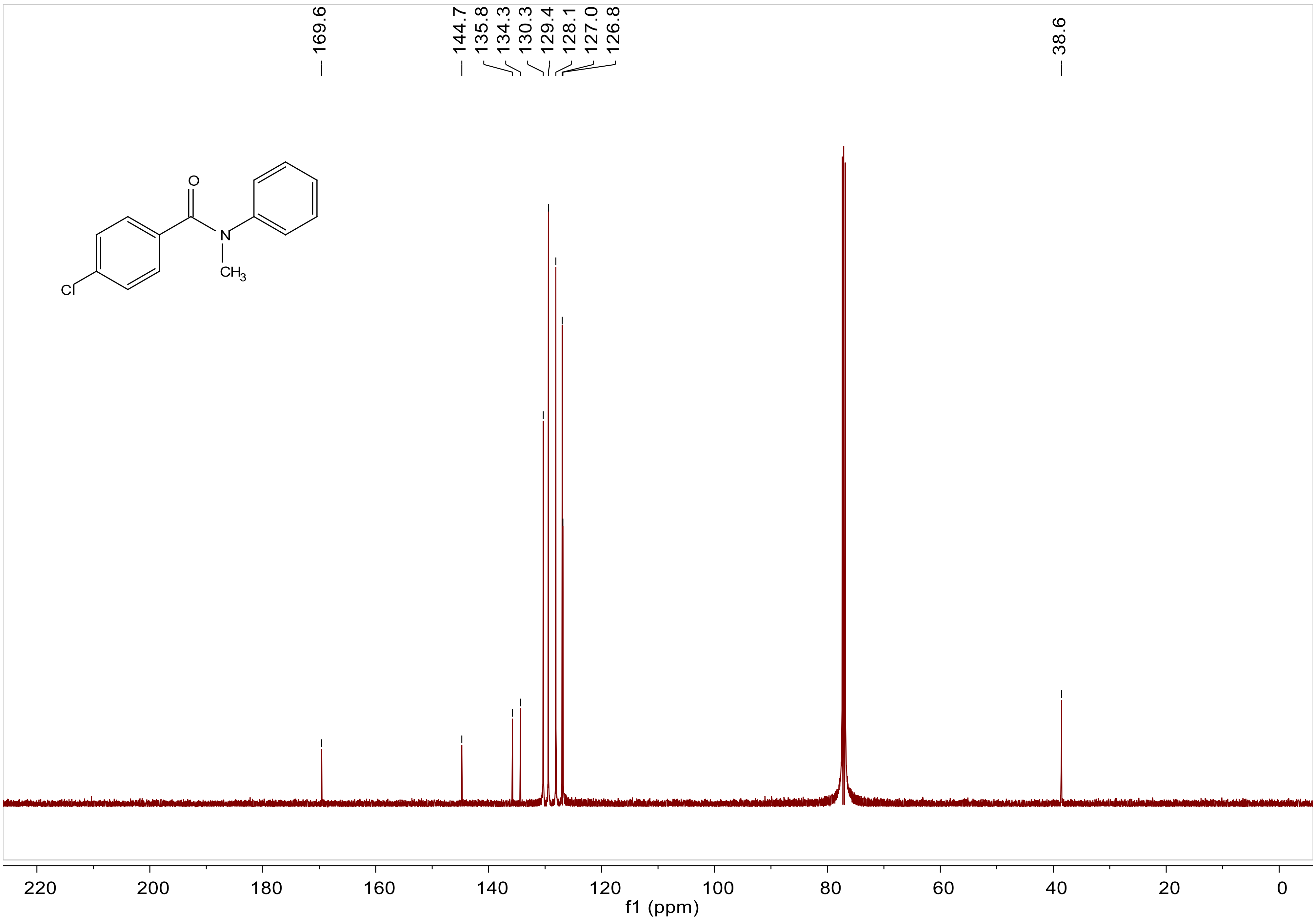
169.6
144.7
135.8
134.3
130.3
129.4
128.1
127.0
126.8
38.6
O
N
CH3
Cl
220
200
180
160
140
120
100
80
60
40
20
0
f1 (ppm)

NMR data of compound 4-fluoro-*N*-methyl-*N*-phenylbenzamide

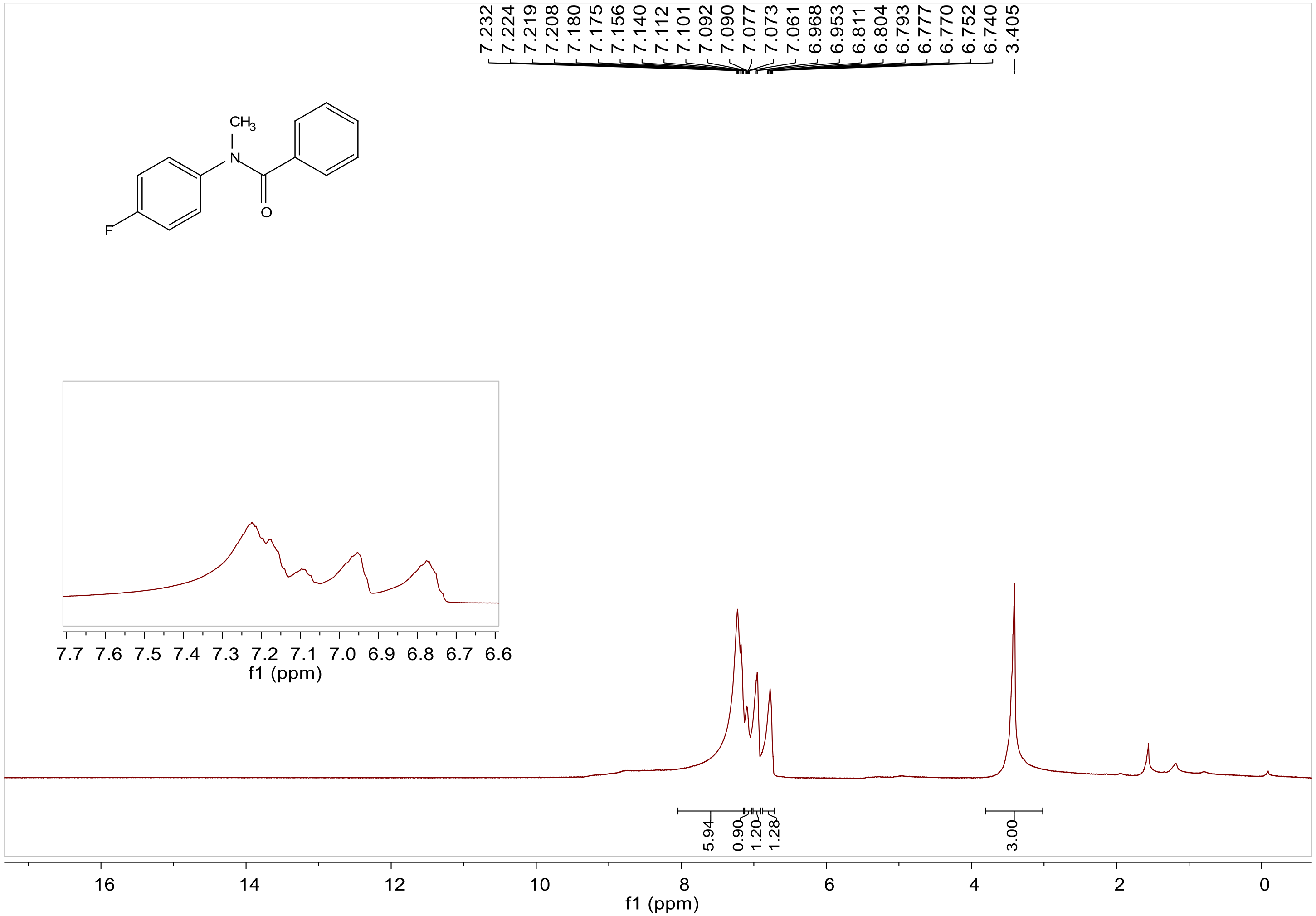

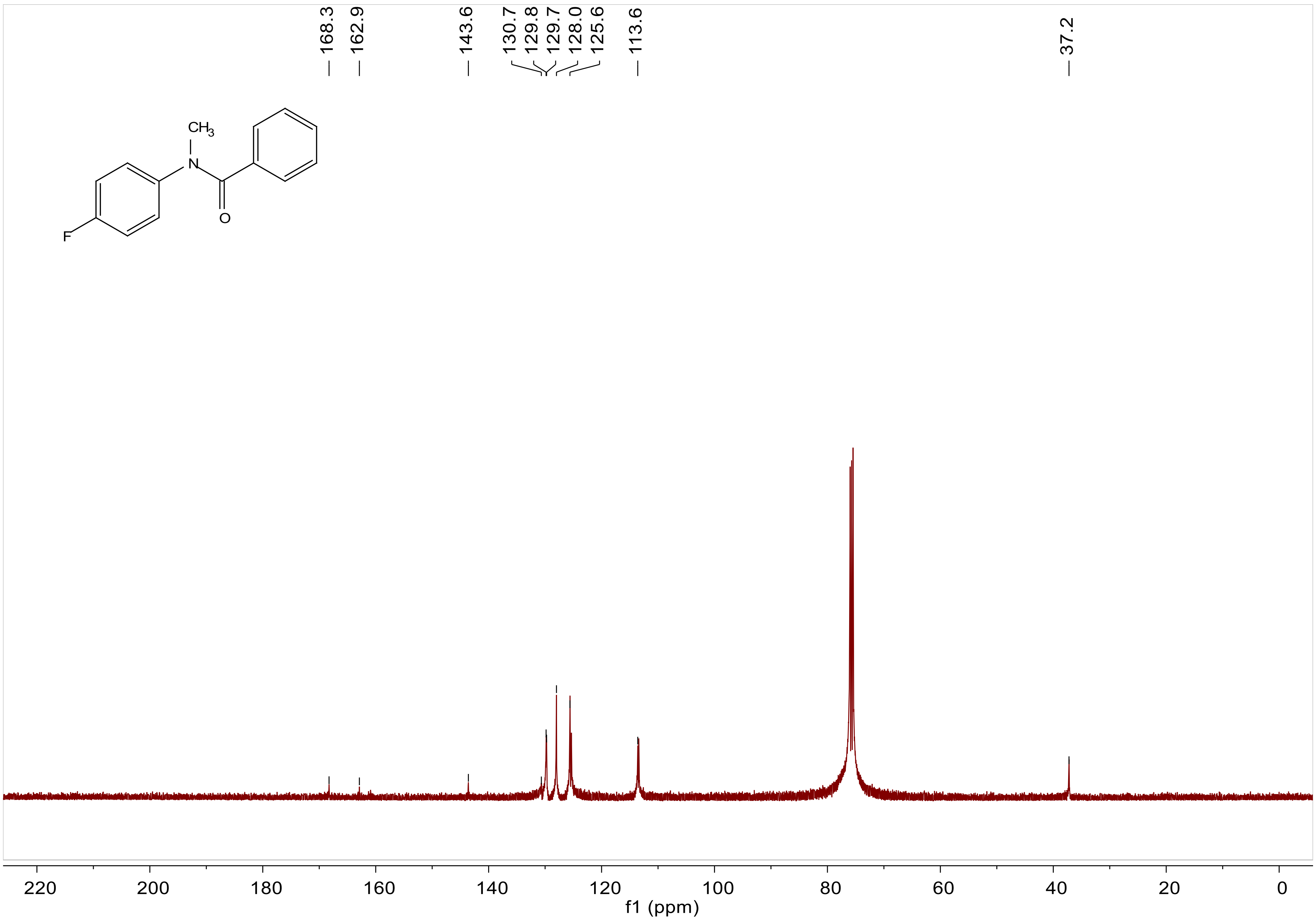
168.3
162.9
143.6
130.7
129.8
129.7
128.0
125.6
113.6
37.2
CH3
N
O
F
220
200
180
160
140
120
100
80
60
40
20
0
f1 (ppm)

NMR data of compound *N*-methyl-*N*-phenyl-4-(trifluoromethyl)benzamide

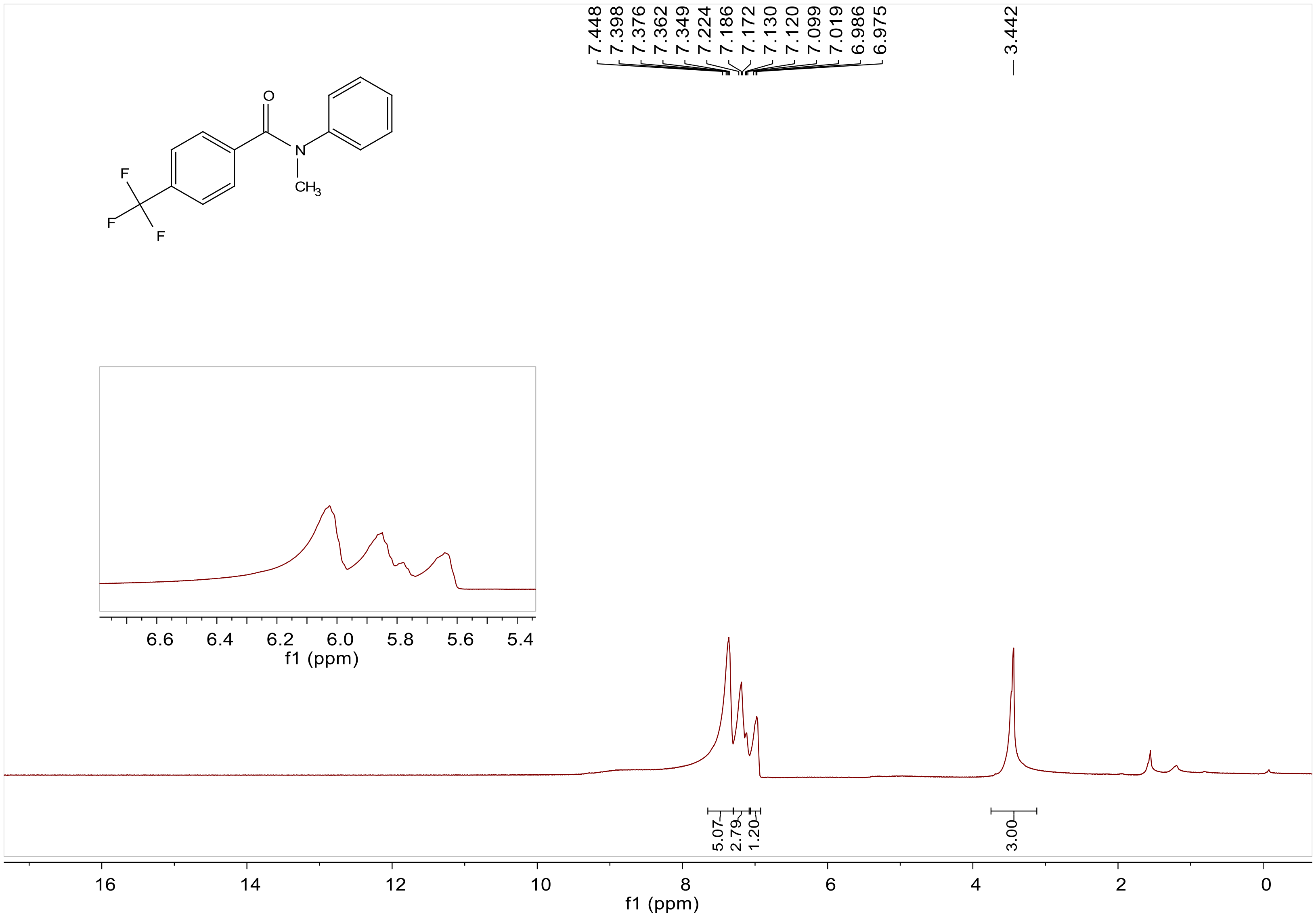

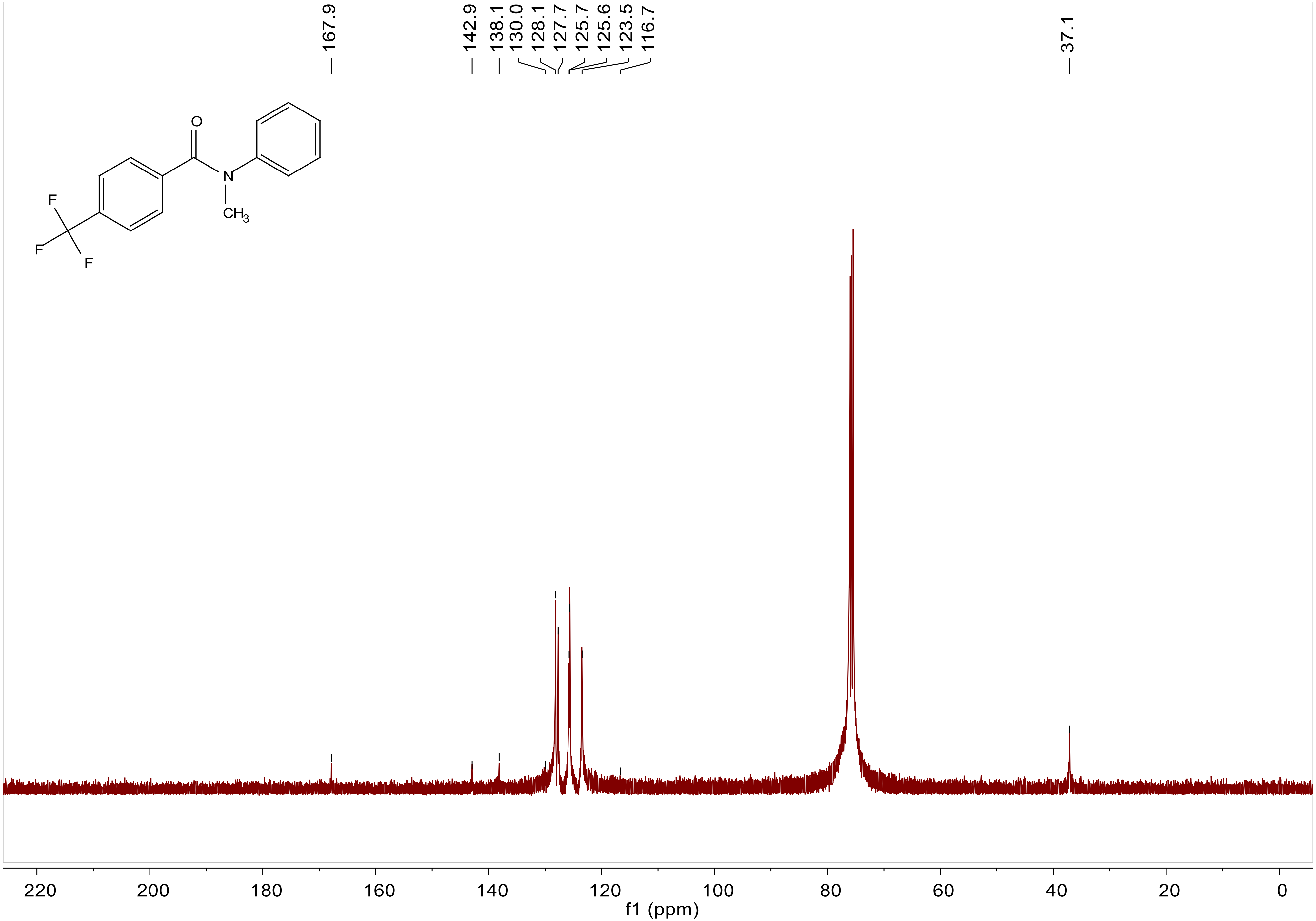

167.9
142.9
138.1
130.0
128.1
127.7
125.7
125.6
123.5
116.7
37.1
O
N
CH3
F
F
F
220
200
180
160
140
120
100
80
60
40
20
0
f1 (ppm)

NMR data of compound *N*-methyl-4-nitro-*N*-phenylbenzamide

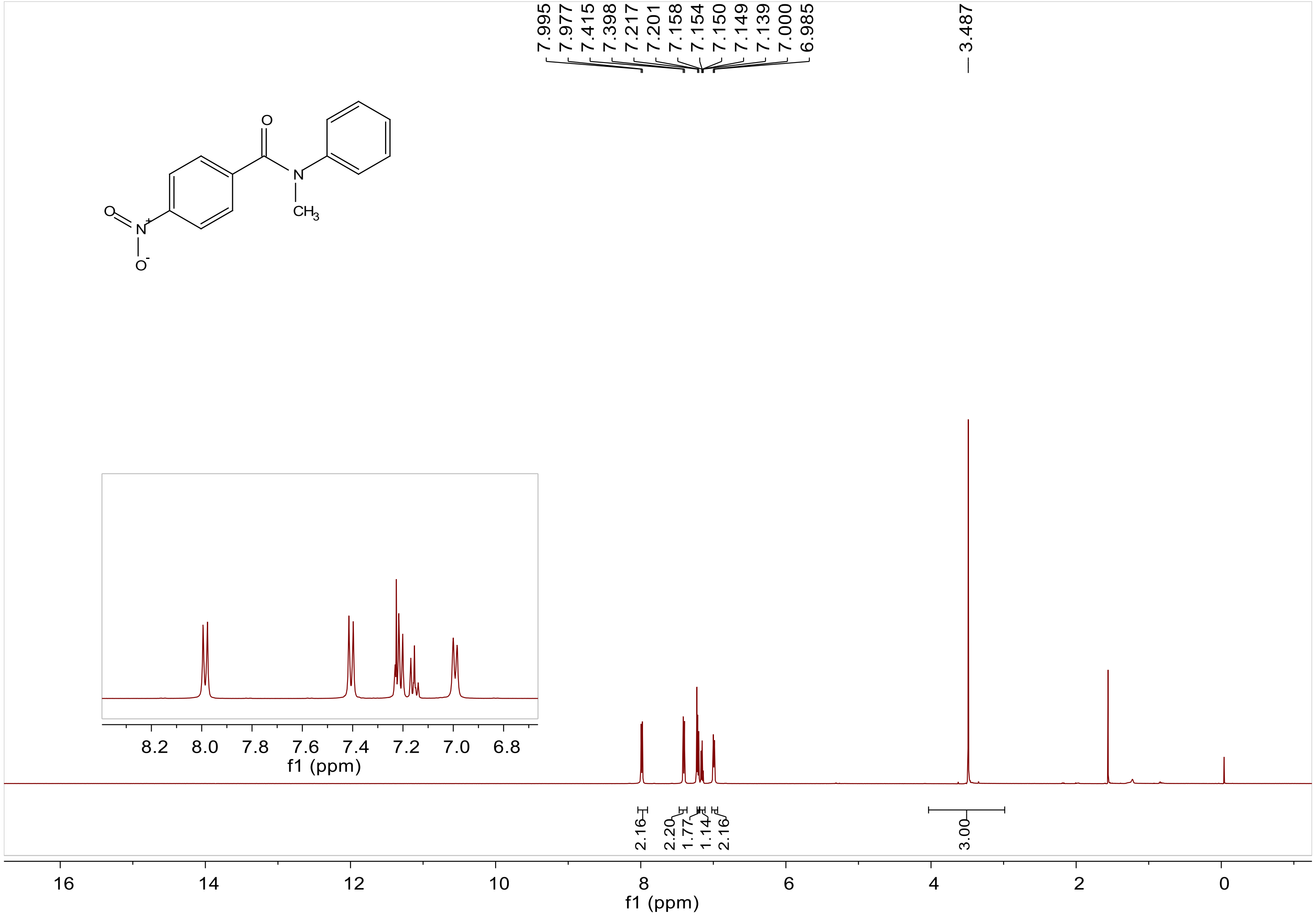

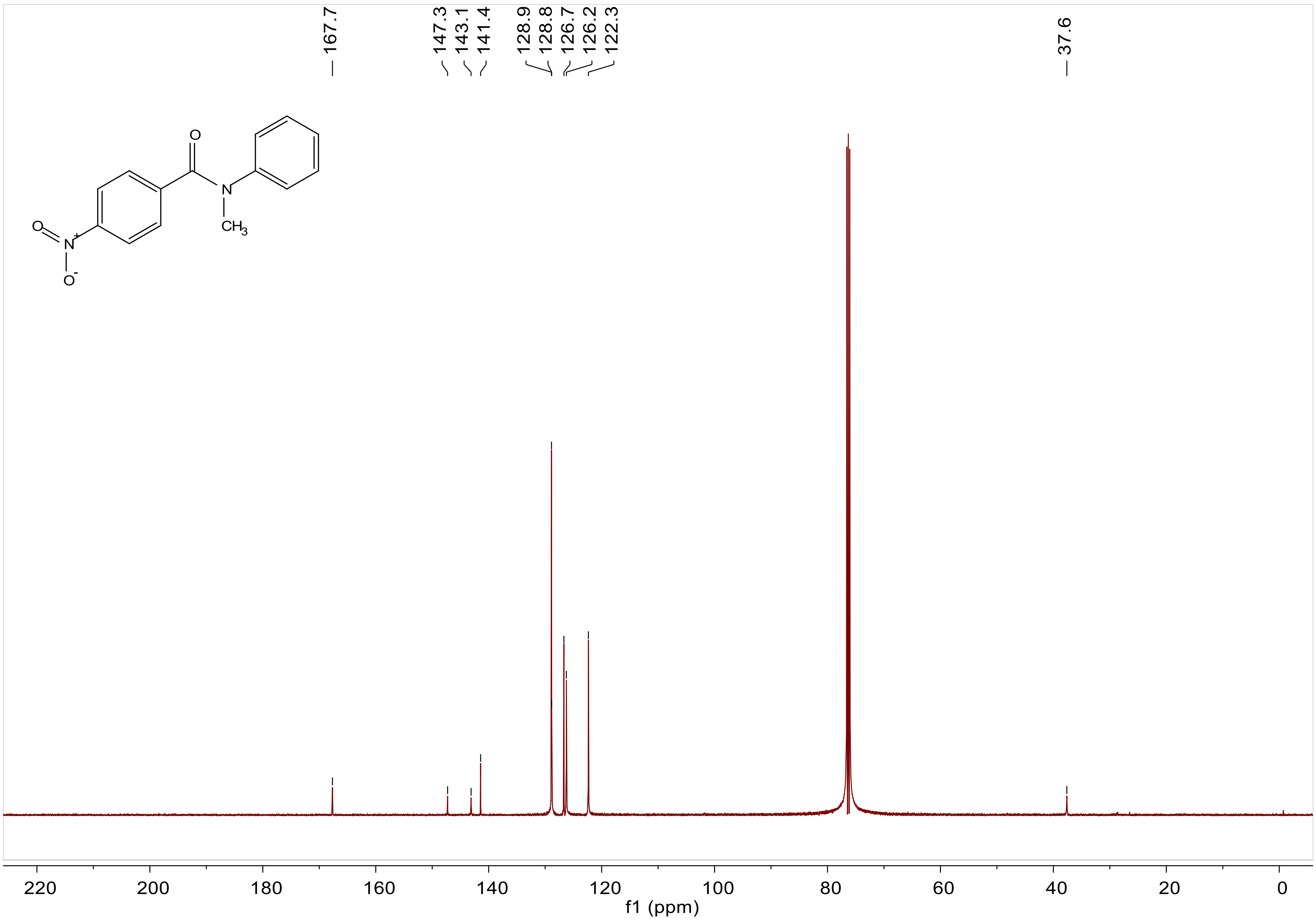

167.7
147.3
143.1
141.4
128.9
128.8
126.7
126.2
122.3
37.6
O
N
CH3
O
N+
O-
220
200
180
160
140
120
100
80
60
40
20
0
f1 (ppm)
